# Synthesis and Anti-Tumor Evaluation of Carboranyl BMS-202 Analogues—A Case of Carborane Not as Phenyl Ring Mimetic

**DOI:** 10.3390/molecules30244789

**Published:** 2025-12-16

**Authors:** Changxian Yuan, Chaofan Li, Chenyang Ma, Yuzhe Lin, Linyuan Wang, Guanxiang Hao, Yirong Zhang, Hongjing Li, Yuan Li, Yu Zhao, Nan Sun, Tiezheng Chen, Zhiguang Zhang, Dengfeng Cheng, Sinan Wang

**Affiliations:** 1 School of Biomedical Engineering & State Key Laboratory of Advanced Medical Materials and Devices, ShanghaiTech University, Shanghai 201210, China; yuanchx2022@shanghaitech.edu.cn (C.Y.); 1203295311@qq.com (C.L.); machy2023@shanghaitech.edu.cn (C.M.); linyzh12023@shanghaitech.edu.cn (Y.L.); wangly2024@shanghaitech.edu.cn (L.W.); haogx2022@shanghaitech.edu.cn (G.H.); zyr119826@126.com (Y.Z.); lihj2023@shanghaitech.edu.cn (H.L.); liyuan2024@shanghaitech.edu.cn (Y.L.); sunnan2023@shanghaitech.edu.cn (N.S.); chentzh2022@shanghaitech.edu.cn (T.C.); chengdf@shanghaitech.edu.cn (D.C.); 2 College of Chemical and Pharmaceutical Engineering, Hebei University of Science and Technology, Shijiazhuang 050018, China; 18801610599@163.com; 3 Shanghai Clinical Research and Trial Center, Shanghai 201210, China; 4 School of Life Science and Technology, ShanghaiTech University, Shanghai 201210, China; zhaoyu@alumni.shanghaitech.edu.cn; 5 Shanghai Institute for Advanced Immunochemical Studies, ShanghaiTech University, Shanghai 201210, China

**Keywords:** carborane, BMS-202, phenyl ring mimetic, antitumor activity

## Abstract

Carborane is considered a three-dimensional mimetic of phenyl rings in medicinal chemistry. BMS-202 is a potent PD-L1 inhibitor that can block the PD-L1/PD-1 interaction and restore the immune response to cancer cells. Herein, we replaced the terminal phenyl group of BMS-202 with carborane and prepared its carboranyl BMS-202 analogues. The results showed a loss of PD-L1 binding affinity due to the bulky size of carborane, suggesting that carborane cannot serve as a phenyl ring mimetic in certain cases. Docking study demonstrated that the narrow binding pocket of PD-L1 could not hold the bulky carborane, resulting in loss of its activity. Compounds **1a** and **1b** exhibited anti-proliferative activities on a broad scope of cancer cell lines. Further studies indicate that compound **1a** can induce cell apoptosis and lead to G1 cell cycle phase arrest. The boron biodistribution study of compound **1a** revealed that the brain/blood uptake ratio was 0.60 ± 0.08, exhibiting a good blood-brain penetration capability.

## 1. Introduction

**Figure 1:**
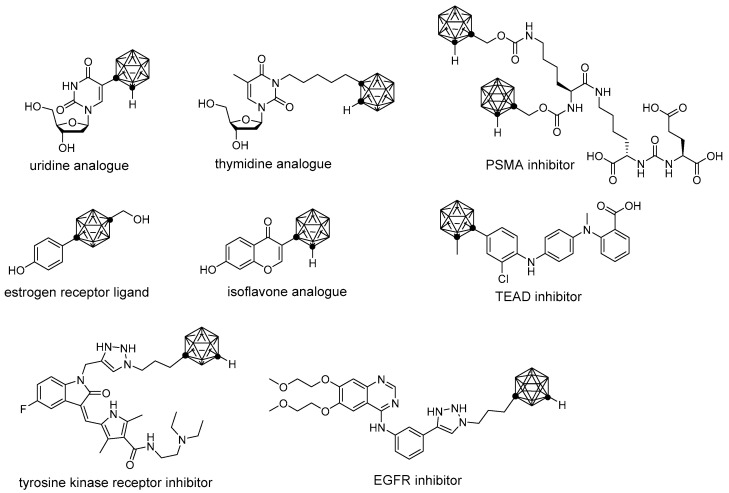
Representative carborane-containing compounds for BNCT or as biological active molecules.

Carboranes are icosahedral boron clusters containing ten BH and two CH vertices [1,2]. Due to their high boron content, they are widely applied as boron delivery agents in boron-neutron capture therapy (BNCT) [3,4,5,6,7,8]. Recently, carborane has gained increasingly attention as a pharmacophore for medicinal chemistry due to its unique features including high hydrophobicity, spherical shape with a volume comparable to a rotational phenyl ring, dihydrogen bond, and resistance toward conventional enzymatic degradation (Figure 1) [9,10,11,12,13,14,15,16,17].

The strategy of replacing a phenyl ring with carborane for drug design is attractive. Phenyl rings are versatile in pharmaceuticals, providing broad compound libraries for this strategy [18,19,20,21,22]. In addition, carborane is bigger and is more hydrophobic than a phenyl ring, which potentially increases the hydrophobic interaction between the ligand and its target. Carborane clusters also exhibit three-dimensional (3D) aromaticity which was proved by both experimental and theoretical studies [23]. Therefore, carborane is usually considered a mimic for aromatic rings in medicinal chemistry [24,25,26]. So far, several studies have used this strategy to modify drugs such as aspirin, flurbiprofen or indomethacin [27,28,29,30].

BMS-202 is the first nonpeptidic small-molecule PD-L1 inhibitor disclosed by Bristol-Myers Squibb [31]. X-ray structural study revealed that BMS-202 could bind to the dimeric PD-L1 at a hydrophobic pocket, induce PD-L1 internalization and block the PD-1/PD-L1 interaction [32,33,34]. These could eventually restore the anti-tumor activities of cytotoxic T cells and enhance their ability to kill cancer cells [35]. So far, a series of small-molecule PD-L1 inhibitors has been developed, most of which contain the diphenyl moiety [36,37,38].

Our group has developed a series of carborane-containing molecules as transcriptional enhanced associate domain (TEAD) inhibitors, carborane-modified natural product analogues, as well as for BNCT agents targeting prostate-specific membrane antigen (PSMA) [39,40,41]. Since BMS-202 contains a biphenyl core that fits into the hydrophobic groove on PD-L1, and carborane is well known for its high hydrophobicity, we hypothesized that replacing the terminal phenyl ring of BMS-202 with o-carborane can offer a better hydrophobic interaction and increase its PD-L1 inhibitory activity. Under this rationale, we synthesized carboranyl BMS-202 analogues and their biological activities were evaluated. The biodistribution of compound **1a** was also measured by testing its boron content with ICP-MS.

## 2. Results and Discussion

### 2.1. Molecular Design

**Figure 2:**
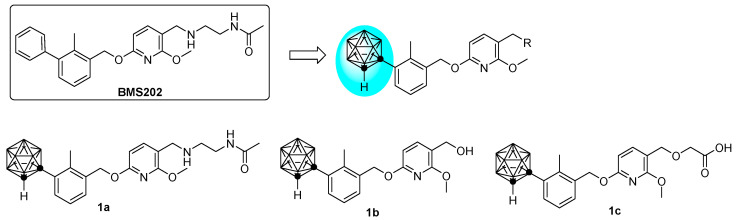
Molecular design of carboranyl BMS-202 analogues.

Studies have shown that the biphenyl ring of BMS-202 has a strong hydrophobic interaction with PD-L1. To increase this hydrophobic interaction and the metabolic stability, we replaced the terminal phenyl ring with a carborane group. The methoxyl pyridyl group was maintained to minimize the change for the whole scaffold. The amino ethyl acetamide moiety was either kept or changed to a hydroxyl group or carboxylic acid group to give compounds **1a**, **1b** and **1c** (Figure 2).

### 2.2. Chemistry

**Scheme 1:**
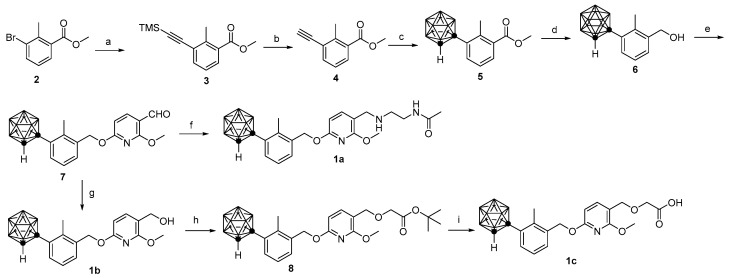
Synthesis of compounds **1a**–**1c**. Reagents and conditions: (a) Ethynyltrimethylsilane, PdCl_2_(PPh_3_)_2_, CuI, Et_3_N, 85 °C, 12 h; (b) K_2_CO_3_, MeOH, rt, 1 h; (c) B_10_H_12_(MeCN)_2_, AgNO_3_, toluene, 100 °C, 4 h; (d) LiAlH_4_, THF, rt, 4 h; (e) Pd(OAc)_2_, t-BuXphos, Cs_2_CO_3_, 6-bromo-2-methoxynicotinaldehyde, toluene, 80 °C, 8 h; (f) *N*-(2-aminoethyl)acetamide, NaBH(OAc)_3_, THF, rt, 12 h; (g) LiAlH_4_, THF, rt, 4 h; (h) NaH, tert-butyl bromoacetate, THF, rt, 12 h; (i) TFA, DCM, rt, 12 h.

**Figure 3:**
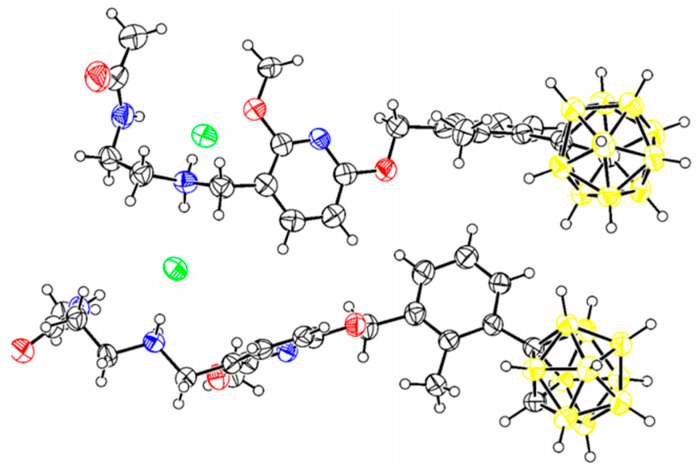
X-ray crystal structure of compound **1a**.

The synthesis route of carboranyl BMS-202 analogues is outlined in Scheme 1. Sonogashira coupling of commercially available compound **2** with trimethylsilyl acetylene followed by deprotection of the TMS group using K_2_CO_3_ formed intermediate **4**. Heating intermediate **4** with B_10_H_12_(MeCN)_2_ converted the terminal alkyne to o-carborane and formed intermediate **5** [42,43,44]. Reducing intermediate **5** with LiAlH_4_ converted the ester group to a primary alcohol and produced compound **6**. Coupling compound **6** with commercially available 6-bromo-2-methoxynicotinaldehyde generated aldehyde **7**. Compound **7** was reacted with *N*-(2-aminoethyl)acetamide using a reductive amination reaction to generate our final product **1a**. The structure of compound **1a** was confirmed by X-ray crystal structure (Figure 3). Compound **1b** was obtained by reducing aldehyde **7** with LiAlH_4._ Reacting compound **1b** with tert-butyl bromoacetate gave intermediate **8**. Compound **1c** was obtained by deprotection of intermediate **8** with TFA.

### 2.3. Biological Evaluation

#### 2.3.1. PD-L1 Inhibition Studies

**Figure 4:**
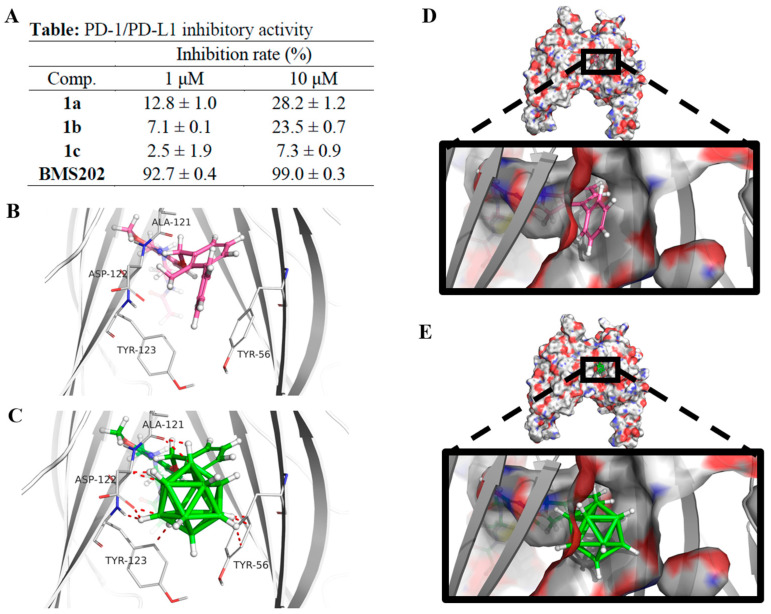
(**A**) PD-1/PD-L1 inhibitory activities of compounds **1a**–**1c**. (**B**) docking of BMS-202 in the binding pocket of PD-L1 dimer (PDB ID: 5N2F) (**C**) docking of **1a** in PD-L1 shows many collisions between the carborane in **1a** and its surrounding residues. The red dashed line represents the collisions. (**D**) docking of BMS-202 and PD-L1 dimer shows that the biphenyl ring can be accommodated in the pocket crevice. (**E**) docking of **1a** and PD-L1 dimer shows that the narrow crevice of PD-L1 dimer can not accommodate the bulky carborane.

Compounds **1a**–**1c** were tested *in vitro* for their inhibitory activities towards PD-1/PD-L1 interactions. The results suggested a loss of inhibitory activities of all three compounds (Figure 4A). Since the only difference between **1a** and BMS-202 is the terminal carborane group, we speculated that the carborane group actually cannot fit into the hydrophobic pocket of PD-L1. Docking studies revealed that the pocket of the PD-L1 dimmer is a narrow crevice that can accommodate the terminal phenyl ring of BMS-202 (Figure 4B,D). In comparison, the spherical bulky carborane causes many collisions in the pocket, suggesting that compound **1a** is not able to fit into the narrow crevice, which leads to the loss of its inhibitory activity towards PD-1/PD-L1 interactions (Figure 4C,E). Due to the lack of boron parameters in existing docking programs, our docking method used the dodecahedrane group to substitute carborane. It mimics the steric effect of carborane but does not account for its electronic features. Although these experiments did not reach the initial goal, they revealed that carborane is not always a mimetic of the phenyl ring. It may be able to increase the hydrophobic interaction with its target when the pocket is big enough to accommodate the spherical carborane group. But in the case of a narrow pocket, carborane can be hindered from entering the pocket due to its three-dimensional spherical shape, resulting in a total loss of biological activities. These results demonstrate that the “carborane replacing phenyl ring” strategy is not a universally effective approach and is dependent on the pocket size of its target protein.

#### 2.3.2. Anti-Proliferation Study

To evaluate the anti-proliferative activities of compounds **1a**–**1c**, six human cancer cell lines were applied for this test: Ramos and Raji, lymphoma; DU145, prostate cancer; HepG2, hepatocellular carcinoma; A549, non-small cell lung cancer; MDA-MB-468, triple-negative breast cancer. Cells were incubated with the tested compounds at various concentrations for 48 h and then their viability was measured through a CCK8 assay [45]. The results are shown in Table 1. Compounds **1a** and **1b** can effectively inhibit cell proliferation with IC_50_ value at a micromolar level. It has a better anti-proliferation effect on Ramos, Raji, DU145 and HepG2, but less active toward A549 and MDA-MB-468. In comparison, compound **1c** showed weak anti-proliferative activities with IC_50_ greater than 50 μM for all the tested cell lines (Table 1, Figure S1). Since compounds **1a** and **1b** showed no PD-L1 inhibition activities, the anti-proliferative activities could arise from other mechanisms such as cell cycle arrest or apoptosis.

#### 2.3.3. Compounds **1a** and **1b** Induce G1 Cell Cycle Phase Arrest

**Figure 5:**
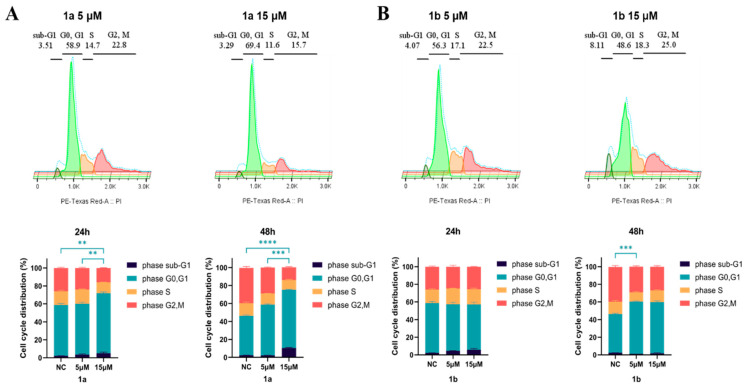
(**A**) cell cycle distribution of compound **1a** (**B**) cell cycle distribution of compound **1b.** The flow cytometry analysis graph shows the result after 24 h of dosing. Statistical analysis is shown on the bar graphs. Data are presented as the mean ± SD of at least three independent experiments. ** *p* < 0.01, *** *p* < 0.001, **** *p* < 0.0001.

To explore the mechanism of compounds **1a** and **1b**’s inhibition on cell proliferation, their effect on the cell cycle was examined using flow cytometry. The experimental data demonstrated that HepG2 incubated with 15 μM of compound **1a** induced significant G0/G1 phase cell cycle arrest. A time-dependent response was observed, with a lower concentration of 5 μM compound **1a** exhibiting significant G0/G1 phase blockade only following a prolonged incubation time of 48 h. Notably, treatment with 15 μM compound **1a** for 48 h generated a distinct sub-G1 apoptotic peak, accounting for approximately 10% of the total cell population. This result correlated well with the previously determined IC_50_ values (Figure 5A). The cell cycle arrest was not observed when compound **1b** was incubated with HepG2 for 24 h. When the incubation time was extended to 48 h, compound **1b** at 5 μM or 15 μM demonstrated comparable G0/G1 phase arrest efficacy. However, their potency remained significantly reduced compared to compound **1a** at equivalent concentrations and exposure durations (Figure 5B).

#### 2.3.4. Apoptosis Study

**Figure 6:**
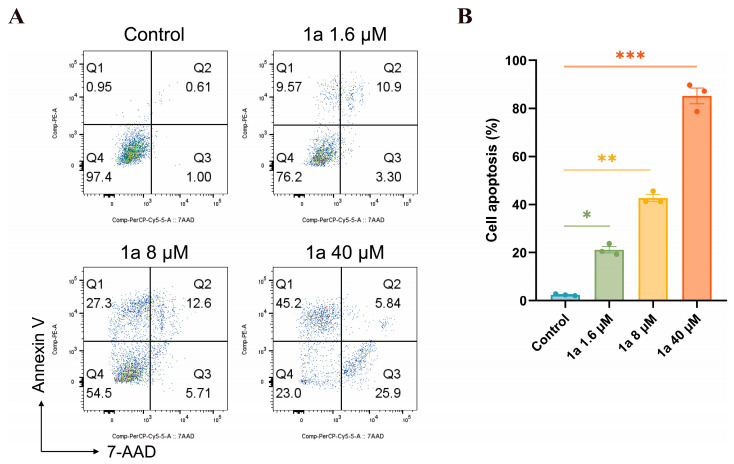
(**A**) The apoptosis analysis of HepG2 cells treated with various concentrations of compound **1a**. Cells that went through early apoptosis were marked with Annexin V-PE^+^/7-AAD^−^, cells that went through late apoptosis were marked with Annexin V-PE^+^/7-AAD^+^. (**B**) The apoptosis rate shown as bar graphs. Data are presented as mean ± SD of three independent experiments. * *p* < 0.05, ** *p* < 0.01, *** *p* < 0.001.

Apoptosis response was evaluated using HepG2 cells treated with compound **1a**. A small number of apoptotic HepG2 cells were observed when cells were treated at sub-therapeutic concentration (10% of the IC_50_ value). At near-IC_50_ concentration, the apoptotic cells accounted for approximately 45% of the total population. Overdosing **1a** at threefold of its IC_50_ value for 24 h resulted in a strong apoptotic response and a dramatic cytotoxic effect, with apoptotic cells increasing to 77% of the total population (Figure 6). These results demonstrated that compound **1a** could induce apoptosis in a dose-dependent manner.

#### 2.3.5. Biodistribution Study

**Figure 7:**
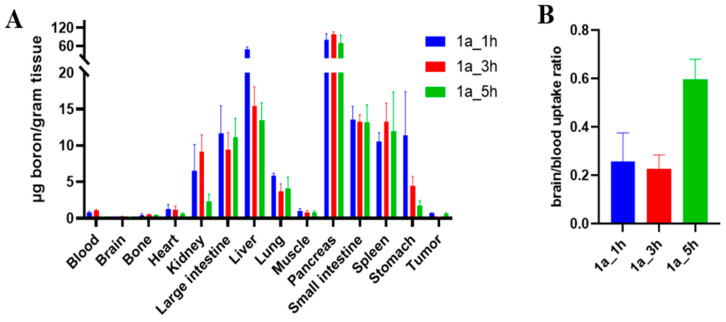
(**A**) In vivo boron biodistribution analysis of compound **1a** at 1 h, 3 h and 5 h post-injection. (**B**) the brain/blood uptake ratio of compound **1a**.

Since these compounds contained boron element, we measured the biodistribution of compound **1a** in HepG2 xenograft mice using boron ICP-MS at 1 h, 3 h and 5 h post-injection. The results were normalized to μg boron per gram tissue and were shown in Figure 7A and Table S1. The boron contents in blood, brain, heart and kidney were slightly increased from 1 h to 3 h post-injection and were dramatically decreased from 3 h to 5 h post-injection. The liver uptake was high up to 48.02 ± 15.3 μg/g at 1 h post-injection and was then decreased to 13.49 ± 4.15 μg/g at 5 h post-injection. Pancreas exhibited a steady and highest boron uptake (79.89 ± 35.39 μg/g at 1 h, 99.06 ± 13.57 μg/g at 3 h and 70.21 ± 44.51 μg/g at 5 h, respectively), which was also observed in our previous boron biodistribution studies when the drug was administered through intraperitoneal injection [40,41]. The boron contents in small intestine, large intestine and spleen were steady around 10 μg/g from 1 h to 5 h. We observed that the brain/blood uptake ratio was 0.60 ± 0.08 at 5 h post-injection, indicating a good penetration capability of compound **1a** through the blood–brain barrier (Figure 7B). We need to point out that absolute boron uptake in brain was 0.13 ± 0.04 μg/g at 5 h post-injection, and the boron uptake in the tumor was below 1 μg/g at all time points, which were all lower than the required 20–35 μg/g for BNCT. In our opinion, this is a reasonable result because the carborane was incorporated as a pharmacophore instead of a boron source for BNCT.

## 3. Materials and Methods

### 3.1. Chemistry

Unless otherwise noted, reagents and solvents were obtained from commercial suppliers and were used without further purification. ^1^H NMR spectra and ^13^C NMR spectra with ^1^H decoupling were recorded on a Bruker 500 MHz spectrometer (Billerica, MA, USA), and chemical shifts were reported in parts per million (ppm) downfield from tetramethylsilane (TMS). Coupling constants (*J* values) were reported in Hertz (Hz). Spin multiplicities are described as s (singlet), br (broad singlet), d (doublet), t (triplet), q (quartet), and m (multiplet). Column chromatography was performed on silica gel (200–300 mesh).

**Methyl 2-methyl-3-((trimethylsilyl)ethynyl)benzoate (3)**

Compound **2** (10.0 g, 43.9 mmol) was dissolved in anhydrous Et_3_N (100 mL) followed by the addition of PdCl_2_(PPh_3_)_2_ (1.5 g, 2.1 mmol), CuI (0.4 g, 2.1 mmol) and ethynyltrimethylsilane (9.3 mL, 65.8 mmol). The reaction mixture was heated to 85 °C and stirred for 12 h. After TLC showed the starting materials were consumed, the solvent was removed under reduced pressure. The mixture was purified by column chromatography (PE/EA = 100:1) to give compound **3** as brown oil (10.5 g, 98% yield). ^1^H NMR (500 MHz, CDCl_3_) *δ* 7.62 (d, *J* = 8.0 Hz, 1H), 7.42 (d, *J* = 8.0 Hz, 1H), 7.01(t, *J* = 8.0 Hz, 1H), 3.73 (s, 3H), 2.54 (s, 3H), 0.11 (s, 9H). ^13^C{^1^H} NMR (126 MHz, CDCl_3_) *δ* 168.0, 142.0, 135.8, 130.4, 129.2, 126.8, 125.3, 103.4, 99.4, 52.1, 18.8, 0.0.

**Methyl 3-ethynyl-2-methylbenzoate (4)**

Compound **3** (10.0 g, 40.6 mmol) was dissolved in MeOH (50 mL). K_2_CO_3_ (11.2 g, 81.1 mmol) was added to the solution. The reaction mixture was stirred for 1 h at room temperature. After TLC showed the starting materials were consumed, the solvent was removed under reduced pressure and the residue was purified by column chromatography (PE/EA = 80:1) to obtain compound **4** as yellow oil (6.8 g, 96% yield). ^1^H NMR (500 MHz, CDCl_3_) *δ* 7.81 (d, *J* = 7.5 Hz, 1H), 7.61 (d, *J* = 7.5 Hz, 1H), 7.19 (t, *J* = 7.5 Hz, 1H), 3.89 (s, 3H), 3.32 (s, 1H), 2.71 (s, 3H). ^13^C{^1^H} NMR (126 MHz, CDCl_3_) *δ* 168.0, 142.2, 136.1, 130.7, 129.2, 125.5, 124.1, 84.5, 82.0, 52.2, 18.8.

**Methyl 2-methyl-3-(1,2-dicarba-closo-dodecarboranyl** **)benzoate (5)**

Compound **4** (5.0 g, 28.7 mmol) was dissolved in anhydrous toluene (50 mL). B_10_H_12_(CH_3_CN)_2_ (8.8 g, 43.5 mmol) and AgNO_3_ (244 mg, 1.4 mmol) were added to the solution. The reaction mixture was heated to 100 °C and stirred for 6 h. After TLC showed that the starting materials were consumed, the reaction was concentrated under reduced pressure and the residue was purified by column chromatography (PE/EA = 50:1) to give compound **5** as a white solid (4.6 g, 54% yield). ^1^H NMR (500 MHz, CDCl_3_) *δ* 7.70 (d, *J* = 8.5 Hz, 1H), 7.59 (d, *J* = 8.5 Hz, 1H), 7.23 (t, *J* = 8.0 Hz, 1H), 4.61 (s, 1H), 3.90 (s, 3H), 2.62 (s, 3H), 3.32–2.04 (br, 10H); ^13^C{^1^H} NMR (126 MHz, CDCl_3_) *δ* 168.9, 135.7, 134.8, 133.8, 133.1, 130.2, 126.2, 77.7, 60.3, 52.6, 19.1. ^11^B NMR (162 MHz, CDCl_3_) δ −1.86, −4.78, −9.19, −10.68, −11.65, −12.71.

**(2-Methyl-3-(1,2-dicarba-closo-dodecarboranyl)phenyl)methanol (6)**

Compound **5** (4.0 g, 13.6 mmol) was dissolved in anhydrous THF (50 mL) and the reaction was cooled to 0 °C in an ice bath. LiAlH_4_ (2.5 M in THF, 22.0 mL) was added dropwise over 10 min. The reaction mixture was warmed to 50 °C and stirred for 4 h. The reaction was cooled to 0 °C in an ice bath. Water was added slowly until no bubbles were generated. 1M HCl (5 mL) was added to the solution, dried over anhydrous Na_2_SO_4_, filtered, and concentrated. Compound **6** was obtained as a white solid (3.4 g, 94% yield). ^1^H NMR (500 MHz, CDCl_3_) *δ* 7.51 (d, *J* = 8.5 Hz, 1H), 7.42 (d, *J* = 7.5 Hz, 1H), 7.19 (t, *J* = 8.0 Hz, 1H), 4.71 (s, 2H), 4.57 (s, 1H), 2.54 (s, 3H), 2.82–1.78 (br, 10H); ^13^C{^1^H} NMR (126 MHz, CDCl_3_) *δ* 141.2, 134.3, 132.4, 130.4, 129.3, 126.3, 78.2, 64.3, 60.6, 16.8. ^11^B NMR (162 MHz, CDCl_3_) δ −1.93, −4.71, −9.13, −10.67, −11.51, −12.72.

**2-Methoxy-6-((2-methyl-3-(1,2-dicarba-closo-dodecarboranyl)benzyl)oxy) nicotinaldehyde (7)**

Compound **6** (3.0 g, 11.3 mmol) was dissolved in anhydrous toluene (30 mL). 6-bromo-2-methoxynicotinaldehyde (2.7 g, 12.5 mmol). Pd(OAc)_2_ (130 mg, 0.58 mmol), Cs_2_CO_3_ (5.6 g, 17.2 mmol), t-BuXphos (724 mg, 1.7 mmol) were added to the solution. The reaction mixture was heated to 100 °C and stirred for 6 h. After TLC showed that the starting materials were consumed, the reaction mixture was filtered and the filter cake was washed by DCM. The filtrate was concentrated under reduced pressure. The residue was washed with petroleum ether three times to give compound **7** as a yellow solid (4.2 g, 93% yield). ^1^H NMR (500 MHz, CDCl_3_) *δ* 10.21 (s, 1H), 8.07 (d, *J* = 8.0 Hz, 1H), 7.56 (d, *J* = 8.0 Hz, 1H), 7.46 (d, *J* = 7.5 Hz, 1H), 7.21 (t, *J* = 8.0 Hz, 1H), 6.44 (d, *J* = 8.5 Hz, 1H), 5.44 (s, 2H), 4.56 (s, 1H), 4.05 (s, 3H), 2.59 (s, 3H), 2.84–1.58 (br, 10H); ^13^C{^1^H} NMR (126 MHz, CDCl_3_) *δ* 187.6, 165.9, 164.9, 140.6, 137.2, 135.2, 132.6, 131.1, 131.0, 126.3, 112.6, 103.7, 78.0, 67.6, 60.7, 54.0, 17.3. ^11^B NMR (162 MHz, CDCl_3_) δ −2.85, −8.80, −9.61, −11.08, −13.38.

***N*-(2-(((6-((3-** **(1,2-Dicarba-closo-dodecarboranyl** **)-2-methylbenzyl)oxy)-2-methoxypyridin-3-yl)methyl)amino)ethyl)acetamide (1a)**

Compound **7** (200 mg, 0.5 mmol) was dissolved in anhydrous THF (5 mL). *N*-(2-aminoethyl)acetamide (76.6 mg, 0.75 mmol) and NaBH(OAc)_3_ (420.5 mg, 2.0 mmol) were added to the solution. The reaction was stirred at room temperature for 4 h. After TLC showed that the starting materials were consumed, the mixture was filtered and the filtrate was concentrated under reduced pressure. The residue was purified by column chromatography (DCM/MeOH = 20:1) to give compound **1a** as a white solid. (132.2 mg, 55% yield). ^1^H NMR (500 MHz, CDCl_3_) δ 7.55–7.52 (m, 2H), 7.45 (d, *J* = 7.0 Hz, 1H), 7.19 (m, 1H), 6.66 (t, *J* = 5.0 Hz, 1H), 6.37 (d, *J* = 8.0 Hz, 1H), 5.35 (s, 2H), 4.57 (s, 1H), 3.99 (s, 3H), 3.92 (s, 2H), 3.48–3.47 (m, 2H), 3.03–3.01 (m, 2H), 2.57 (s, 3H), 2.01 (s, 3H), 2.81–1.64 (br, 10H); ^13^C{^1^H} NMR (126 MHz, CDCl_3_): *δ* 171.8, 163.1, 161.0, 143.5, 137.5, 135.2, 132.4, 131.0, 131.0, 126.2, 104.4, 102.3, 78.1, 67.1, 60.7, 53.9, 50.3, 46.9, 45.6, 36.1, 23.0, 17.2. ^11^B NMR (162 MHz, CDCl_3_) δ −2.91, −8.69, −10.13, −11.10, −13.08. HRMS (ESI positive mode, *m*/*z*) calcd for C_21_H_36_B_10_N_3_O_3_ [M + H]^+^: 487.3718, found: 487.3724.

**(6-((3-** **(1,2-Dicarba-closo-dodecarboranyl** **)-2-methylbenzyl)oxy)-2-methoxypyridin-3-yl)methanol (1b)**

Compound **7** (1.0 g, 2.5 mmol) was dissolved in anhydrous THF (10 mL) and the reaction was cooled to 0 °C in an ice bath. LiAlH_4_ (2.5M in THF, 4 mL) was added dropwise over three minutes. The reaction mixture was stirred for 1 h at room temperature. Water was added slowly until no bubbles were generated. 1M HCl (3 mL) was added to the solution. The mixture was dried over anhydrous Na_2_SO_4_, filtered, and concentrated to give compound **1b** as a white solid (950 mg, 95% yield). ^1^H NMR (500 MHz, CDCl_3_) *δ* 7.54–7.45 (m, 3H), 7.19–7.16 (m, 1H), 6.34 (d, *J* = 8.0 Hz, 1H), 5.35 (s, 2H), 4.57 (s, 3H), 3.96 (s, 3H), 2.58 (s, 3H), 3.17–1.91 (br, 10H); ^13^C{^1^H} NMR (126 MHz, CDCl_3_) *δ* 161.6, 160.3, 140.6, 138.1, 135.1, 132.4, 130.9, 130.8, 126.2, 114.7, 101.3, 78.2, 66.9, 60.7, 60.6, 53.5, 17.2. ^11^B NMR (162 MHz, CDCl_3_) δ −2.78, −8.76, −9.43, −10.93, −13.26. HRMS (ESI positive mode, *m*/*z*) calcd for C_17_H_28_B_10_NO_3_ [M + H]^+^: 403.3031, found: 403.3030.

**tert-Butyl 2-((6-((3-(1,2-dicarba-closo-dodecarboranyl)-2-methylbenzyl)oxy)-2-methoxypyridin-3-yl)methoxy)acetate (8)**

Compound **1b** (200 mg, 0.50 mmol) was dissolved in anhydrous THF (5 mL), and 60% NaH (80 mg, 2.00 mmol) was added to the solution. Then tert-butyl 2-iodoacetate (181.5 mg, 0.75 mmol) was added and the reaction was stirred at room temperature for 12 h. After TLC showed that the starting materials were consumed, the reaction was quenched with water, extracted with ethyl acetate three times. The combined organic layer was washed with brine. The organic layer was dried over Na_2_SO_4_, concentrated under reduced pressure, and the residue was purified by column chromatography (DCM/MeOH = 100:1) to give compound **8** as a white solid. (159.3 mg, 62% yield). ^1^H NMR (500 MHz, CDCl_3_) δ 7.61 (d, *J* = 8.0 Hz, 1H), 7.53 (d, *J* = 8.5 Hz, 1H), 7.46 (d, *J* = 7.5 Hz, 1H), 7.18 (t, *J* = 8.0 Hz, 1H), 6.35 (d, *J* = 8.0 Hz, 1H), 5.34 (s, 2H), 4.57 (s, 1H), 4.54 (s, 2H), 4.00 (s, 2H), 3.92 (s, 3H), 2.98–1.94 (br, 10H), 2.57 (s, 3H), 1.48 (s, 9H); ^13^C{^1^H} NMR (126 MHz, CDCl_3_): *δ* 169.7, 161.7, 160.3, 141.8, 138.1, 135.1, 132.4, 130.9, 130.8, 126.1, 111.4, 101.5, 81.6, 78.2, 68.1, 67.2, 66.8, 60.6, 53.5, 28.2, 17.2. ^11^B NMR (162 MHz, CDCl_3_) δ −2.84, −8.77, −9.45, −11.11, −13.35.

**2-((6-((3-** **(1,2-Dicarba-closo-dodecarboranyl** **)-2-methylbenzyl)oxy)-2-methoxypyridin-3-yl)methoxy)acetic acid (1c)**

Compound **8** (100 mg, 0.19 mmol) was dissolved in DCM (5 mL), and TFA (3 mL) was added to the solution. The reaction was stirred at room temperature for 12 h. After TLC showed that the starting materials were consumed, saturated NaHCO_3_ solution was added dropwise to adjust the pH to neutral. The product was extracted with DCM, concentrated under reduced pressure, and the residue was purified by column chromatography (DCM/MeOH = 50:1). Compound **1c** was obtained as a white solid (84.7 mg, 97% yield). ^1^H NMR (500 MHz, CDCl_3_) *δ* 7.54 (t, *J* = 8.0 Hz, 2H), 7.46 (d, *J* = 7.5 Hz, 1H), 7.19 (t, *J* = 8.0 Hz, 1H), 6.34 (d, *J* = 8.0 Hz, 1H), 5.35 (s, 2H), 5.04 (s, 2H), 4.58 (s, 1H), 3.95 (s, 3H), 2.57 (s, 2H), 2.07 (s, 3H), 2.97–1.64 (br, 10H); ^13^C{^1^H} NMR (126 MHz, CDCl_3_): *δ* 171.1, 162.1, 160.8, 142.3, 138.0, 135.2, 132.4, 131.0, 130.9, 126.2, 109.8, 101.4, 78.2, 66.9, 61.0, 60.6, 53.6, 21.1, 17.2. ^11^B NMR (162 MHz, CDCl_3_) δ −2.81, −8.77, −9.46, −10.96, −13.31. HRMS (ESI negative mode, *m*/*z*) calcd for C_19_H_28_B_10_NO_5_ [M–H]^−^: 459.2940, found: 459.2945.

### 3.2. Biological Evaluation

#### 3.2.1. PD1/PD-L1 Blockade Bioassay

The HTRF PD1/PD-L1 binding assay was performed using PD1/PD-L1 binding kits (CisbioBioassays SAS, Codolet, France, part no. 64PD1PEG) according to the manufacturer’s instructions. The interaction between PD1 and PD-L1 is detected by anti-Tag1 Eu Cryptate reagent (HTRF donor) and anti-Tag2 XL665 antibody (HTRF acceptor). Synthesized compounds blocking the PD1/PD-L1 interaction cause a reduction in the HTRF signal. Briefly, 2 µL of compounds, 4 µL of Tag1-PD-L1 and 4 µL of Tag2-PD1 were added sequentially to a 384-well plate for a total volume of 10 µL and incubated for 15 min at room temperature. Subsequently 10 µL of pre-mixed anti-Tag1-EuK and anti-Tag2-XL665 reagent was added into the assay well and then incubated for 1 h at room temperature. The signals were measured at 665 nm/620 nm and HTRF ratio was calculated with (OD_665 nm_/OD_620 nm_) × 10^4^. Inhibition rate (%) = (Ratio_max_ − Ratio_sample_)/(Ratio_max_ − Ratio_min_) × 100.

#### 3.2.2. Docking Studies

All the ligands were drawn and optimized using Chem3D software (version 22.2.0). Energy minimization calculations were performed using the MM2 calculation in Chem3D. Existing docking programs generally lack parameters for boron. Therefore, we replaced the carborane group with a structurally similar adamantane group for docking. These new ligands, incorporating adamantane groups, were then redrawn and subjected to another MM2 energy minimization. The protein structure was downloaded from the RCSB Protein Data Bank in PDB format (PDB ID: 5N2F). For docking studies, protein preparation was performed using the Protein Preparation Workflow in Schrödinger’s Maestro suite (version 22-3). The docking site was defined using the Receptor Grid Generation function of Maestro by selecting amino acids near the original ligand. Subsequently, docking was performed using the Ligand Docking function. This docking incorporated core constraints, defined by a core pattern comparison with the original protein ligand’s core atoms, specifically utilizing a Maximum Common Substructure approach. Finally, the carborane-containing ligand was aligned to the adamantane ligand docking result using the Ligand Alignment function [46,47].

#### 3.2.3. Cell Culture

The cell lines Raji, Ramos, HepG2, MDA-MB-468, DU145 and A549 were obtained from the American Type Culture Collection (ATCC, Manassas, VA, USA). Raji and Ramos were cultured in RPMI-1640 supplemented with 10% fetal bovine serum (FBS), 100 U penicillin, and 100 mg/mL streptomycin. HepG2 and MDA-MB-468 were maintained in DMEM supplemented with 10% FBS, 100 U penicillin, and 100 mg/mL streptomycin. DU145 cells were routinely cultured in MEM supplemented with 10% FBS, 100 U penicillin, and 100 mg/mL streptomycin. A549 cells were cultured in Ham’s F-12K supplemented with 10% FBS, 100 U penicillin, and 100 mg/mL streptomycin. Cultures were incubated in a humidified incubator at 37 °C and 5% CO_2_. Cells were subcultured every 3–4 days. For HepG2, DU145, A549 and MDA-MB-468 cell lines, cells were removed from flasks for passage or for transfer to assay plates by incubating them with 0.25% trypsin.

#### 3.2.4. Anti-Proliferation Assay

Cell proliferation was determined by a CCK8 assay (CCK-8 cell counting kit, catalog number A311-02, Vazyme, Nanjing, China) using 2-(2-methoxy-4-nitrophenyl)-3-(4-nitrophenyl)-5-(2,4-disulfophenyl)-2*H*-tetrazolium, monosodium salt (WST-8) to measure cell viability. Briefly, Raji, Ramos, HepG2, DU145, A549 and MDA-MB-468 were seeded into 96-well transparent plates at a density of 4000 cells per well. Various concentrations of the tested compounds or solvent control were added to the wells in triplicate. The cells were incubated at 37 °C with 5% CO_2_ for 48 h. 10 μL of WST-8 was added to each well and the cells were incubated at 37 °C with 5% CO_2_ for 4 h. The absorbance value of each well was measured by a microplate reader (SpectraMax i3x, Molecular Devices, San Jose, CA, USA) at a wavelength of 450 nm. The IC_50_ values were calculated using nonlinear regression analysis with GraphPad Prism 9 software.

#### 3.2.5. Cell Cycle Analysis by Flow Cytometry

HepG2 cells were seeded on a 24-well plate at a density of 50,000 cells per well and cultured overnight. Then cells were treated in triplicate with either 0.1% DMSO as a control group, compound **1a** (5 μM and 15 μM) or compound **1b** (5 μM and 15 μM). Following 24 h or 48 h treatment, cells were harvested by trypsin (no EDTA), washed with cold PBS, and mixed with pre-cooled 70% ethanol and fixed at 4 °C for 4 h. After washing with cold PBS, cells were treated with RNase A and stained with propidium iodide (cell cycle and apoptosis analysis kit, catalog No. 40301ES60, Yeasen, Shanghai, China). The samples were analyzed using a flow cytometer (LSRFortessa, BD Biosciences, Franklin Lakes, NJ, USA), with the data acquired from 20,000 cells in general. The results were analyzed using FlowJo v10.10 software.

#### 3.2.6. Apoptosis Analysis Assay

HepG2 cells were seeded on a 6-well plate at a density of 200,000 cells per well and cultured overnight. Cells were then exposed in triplicate to either 0.1% DMSO as a control group or compound **1a** (1.6 μM, 8 μM and 40 μM) and incubated for 24 h. Annexin V-PE/7-AAD apoptosis detection kit (catalog No. A213-02, Vazyme) was used based on the manufacturer’s instructions to detect the apoptosis rates. In brief, cells were washed twice in ice-cold PBS and incubated with 100 μL 1 × binding buffer supplemented with 5 μL Annexin V-PE and 5 μL 7-AAD staining solution. 30 min later, another 400 μL 1 × binding buffer was added. Apoptotic rate was measured by flow cytometry, and the data were analyzed by FlowJo v10.10 software. Live cells were defined as Annexin V^−^/7-AAD^−^, early apoptosis as Annexin V^+^/7-AAD^−^ and late apoptosis as Annexin V^+^/7-AAD^+^.

#### 3.2.7. Xenograft Models

All animal studies were conducted in compliance with Institutional Animal Care and Use Committee guidelines at ShanghaiTech University (Approval number: 20240407002). Male BALB/c nu/nu nude mice (4–6 weeks old) were purchased from Shanghai Jihui Laboratory Animal Care Co., Ltd. (Shanghai, China). The mice were maintained in air-conditioned rooms and fed with standard laboratory food and water. For tumor inoculation, HepG2 cells were trypsinized, washed, centrifuged, and resuspended in PBS, followed by mixing with matrigel (Xiamen Mogengel, Xiamen, China) at a 1:1 ratio. A total amount of 5 × 10^6^ cells was subcutaneously injected into the flank region of each mouse. Mice were enrolled in experiments when the average tumor volume reached 300–500 mm^3^.

#### 3.2.8. Biodistribution Study of Compound **1a** Using ICP-MS

1 mg of compound **1a** was administered in a mixture of saline and DMSO through a single intraperitoneal injection. Mice were sacrificed at 1 h, 3 h and 5 h post-injection (n = 3 per time point). Blood was collected by cardiac puncture. Major organs (bone, brain, heart, kidney, large intestine, liver, lung, muscle, pancreas, small intestine, spleen, stomach, and subcutaneous tumor) were harvested and weighed. Tissue digestion was performed for 2 days at room temperature in 1 mL of a 1:1 mixture of concentrated sulfuric and nitric acids. After vortexing and thorough mixing, 100 μL of the digestion solution was taken out and diluted 100-fold in 9.9 mL of DI water followed by filtering through a 0.22 μm membrane. Boron content was then quantified using ICP-Mass (NexION 2000, PerkinElmer, Waltham, MA, USA).

#### 3.2.9. Statistical Analysis

Data were analyzed using the unpaired two-tailed Student’s t test for comparisons between two groups and one-way ANOVA for comparisons involving more than two groups. Differences at the 95% confidence level (*p* < 0.05) were considered statistically significant. The results are presented as mean ± SD and plotted using GraphPad Prism 9 software.

## 4. Conclusions

In this study, we applied the “carborane replacing phenyl ring” strategy to the PD-L1 small molecule inhibitor BMS-202 and made its carboranyl derivatives. Unfortunately, the carboranyl derivatives of BMS-202 lost the binding affinity to PD-L1. The docking study revealed that PD-L1 holds the biphenyl ring of BMS-202 in a narrow crevice, which was not big enough to hold the spherical bulky carborane. These results remind medicinal chemists that they need to consider the pocket size of the target protein when using the “carborane replacing phenyl ring” strategy to design molecules. To our delight, compounds **1a** and **1b** exhibited anti-proliferative activities to a variety of cancer cell lines. The mechanism studies revealed that compound **1a** can induce cell apoptosis and lead to G1 cell cycle phase arrest. In the biodistribution study, we found that compound **1a** was accumulated in the liver and pancreas at a high uptake value when it was administered through intraperitoneal injection. The brain/blood uptake ratio was 0.60 ± 0.08, indicating a good blood-brain penetration capability.

## Figures and Tables

**Table 1:** *In vitro* antiproliferative activities of carboranyl BMS-202 analogues.

	IC_50_ (μM)					
**ID**	**Ramos**	**Raji**	**DU-145**	**HepG2**	**A549**	**MDA-MB-468**
**1a**	3.5 ± 1.0	3.9 ± 1.0	9.5 ± 1.2	13.3 ± 1.2	67.2 ± 1.2	70.5 ± 1.0
**1b**	2.5 ± 1.0	3.5 ± 1.1	10.4 ± 1.4	26.1 ± 1.1	99.4 ± 1.2	38.5 ± 1.1
**1c**	57.6 ± 1.1	169.3 ± 1.2	80.4 ± 1.3	110.9 ± 1.2	149.5 ± 1.0	52.9 ± 1.2

Data were expressed as means ± SD (*n* ≥ 4).

## Data Availability

Data are contained within the article and Supplementary Materials.

## Supplementary Materials

The following supporting information can be downloaded at: https://www.mdpi.com/article/10.3390/molecules30244789/s1, Accession Codes: Crystallographic data are available free of charge from the Cambridge Crystallographic Data Centre via www.ccdc.cam.ac.uk/data_request/cif (accessed on 9 October 2025). The CCDC number for compound **1a** is assigned as 2482386. Figure S1: IC_50_ of compounds **1a**, **1b** and **1c** towards various cancer cell lines; Table S1: In vivo boron biodistribution analysis of compound **1a**. ^1^H and ^13^C NMR Spectra Data. X-ray crystallographic structure of compound **1a** (Tables S2–S9).
