# Supplementary material for: Synthesis and Anti-Tumor Evaluation of Carboranyl BMS-202 Analogues—A Case of Carborane Not as Phenyl Ring Mimetic
Source: Molecules. 2025 Dec 16;30(24):4789. doi: 10.3390/molecules30244789 (PMC12735689; doi:10.3390/molecules30244789)
Supplement: Supplementary file 1 [file molecules-30-04789-s001.zip › molecules-3949075-supplementary.pdf]

Supporting Information

Synthesis and Anti-tumor Evaluation of Carboranyl BMS-202 Analogues – an Example of Carborane not as Phenyl Ring Mimetic

Changxian Yuan[1,†], Chaofan Li[1,2,†], Chenyang Ma[1], Yuzhe Lin[1], Linyuan Wang[1], Guanxiang Hao[1], Yirong Zhang[1], Hongjing Li[1], Yuan Li[1,3], Yu Zhao[4,5], Nan Sun[1,4], Tiezheng Chen[1], Zhiguang Zhang[2], Dengfeng Cheng[1,3], Sinan Wang[1],*

[1] School of Biomedical Engineering & State Key Laboratory of Advanced Medical Materials and Devices, ShanghaiTech University, Shanghai 201210, China.
[2] College of Chemical and Pharmaceutical Engineering, Hebei University of Science and Technology, Shijiazhuang 050018, China.
[3] Shanghai Clinical Research and Trial Center, Shanghai, 201210, China.
[4] School of Life Science and Technology, ShanghaiTech University, Shanghai 201210, China.
[5] Shanghai Institute for Advanced Immunochemical Studies, ShanghaiTech University, Shanghai 201210, China.

* Corresponding author at: School of Biomedical Engineering & State Key Laboratory of Advanced Medical Materials and Devices, ShanghaiTech University, Shanghai 201210, China
E-mail address: wangsn@shanghaitech.edu.cn (Sinan Wang).
† These authors contributed equally to this work.

Table of contents

Figure S1. IC_{50} of compounds **1a**, **1b** and **1c** towards various cancer cell lines……..S2

Table S1. I*n vivo* boron biodistribution analysis of compound **1a** ……………..……S2

^{1}H, ^{13}C and ^{11}B NMR spectra data…………………….……………………..………..S3

High-resolution mass spectra…………….………………………………...……..S16

HPLC data of final compounds…………………………………..……...……..S17

Determination of X-ray crystallographic structure of compound **1a**………...........S19

Figure S1. IC_{50} of compounds **1a**, **1b** and **1c** towards various cancer cell lines

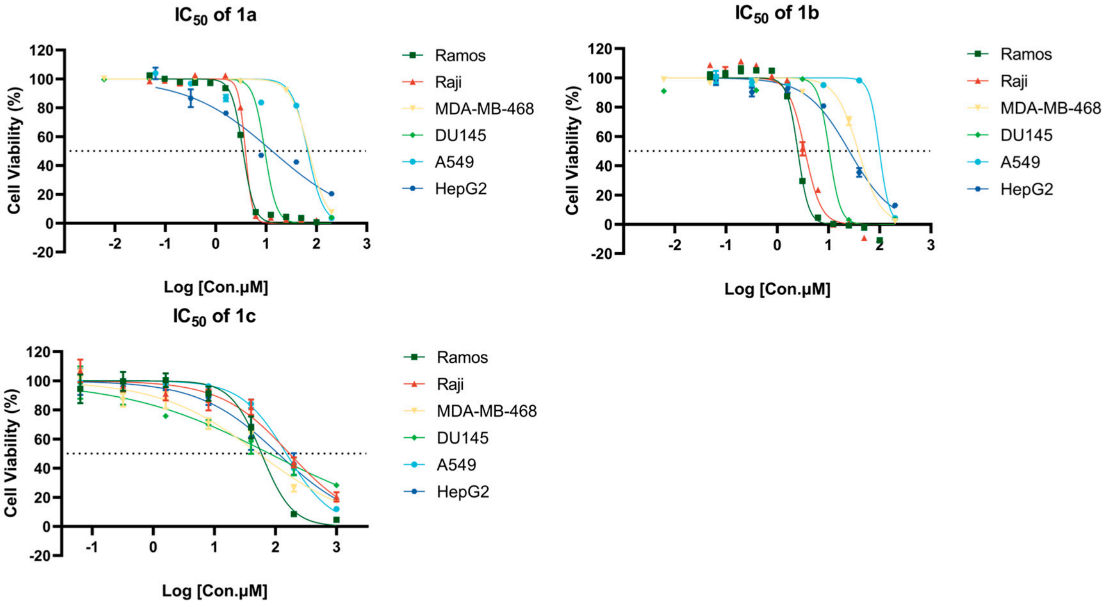

Table S1. I*n vivo* boron biodistribution analysis of compound 1a at 1h、3h and 5h post-injection[a]

	**1h**	**3h**	**5h**
Blood	0.81 ± 0.28	1.08 ± 0.21	0.22 ± 0.03
Brain	0.2 ± 0.07	0.26 ± 0.1	0.13 ± 0.04
Bone	0.46 ± 0.32	0.51 ± 0.08	0.42 ± 0.08
Heart	1.27 ± 1.07	1.21 ± 0.81	0.62 ± 0.2
Kidney	6.51 ± 6.29	9.15 ± 4.06	2.3 ± 1.74
Large intestine	11.7 ± 6.56	9.46 ± 4.03	11.12 ± 4.6
Liver	48.02 ± 15.3	15.48 ± 4.48	13.49 ± 4.15
Lung	5.88 ± 0.57	3.76 ± 1.75	4.14 ± 2.62
Muscle	1.01 ± 0.57	0.76 ± 0.55	0.82 ± 0.3
Pancreas	79.89 ± 35.39	99.06 ± 13.57	70.21 ± 44.51
Small intestine	13.56 ± 3.16	13.28 ± 1.72	13.19 ± 4.16
Spleen	10.6 ± 2.04	13.26 ± 4.45	12.03 ± 9.25
Stomach	11.36 ± 10.49	4.45 ± 2.2	1.76 ± 1.16
Tumor	0.66 ± 0.19	0.18 ± 0.06	0.58 ± 0.34

[a] value unit is ug boron/gram tissue

^{1}H NMR, ^{13}C NMR and ^{11}B NMR spectra

methyl 2-methyl-3-((trimethylsilyl)ethynyl)benzoate (3)

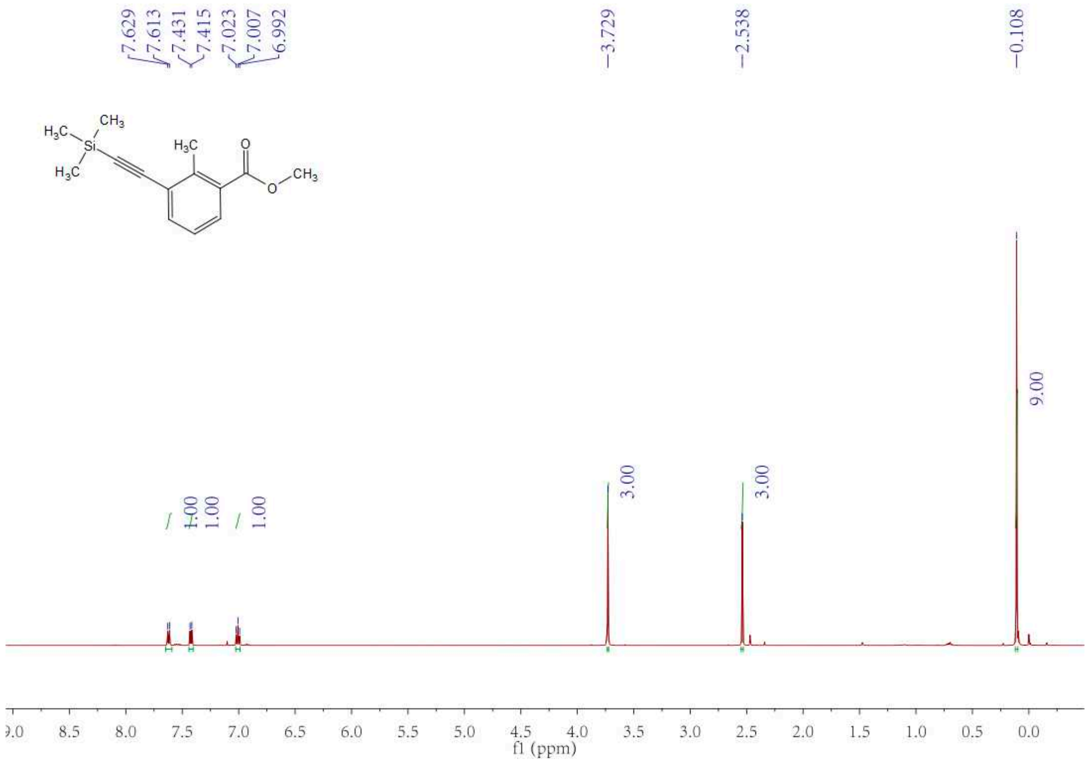

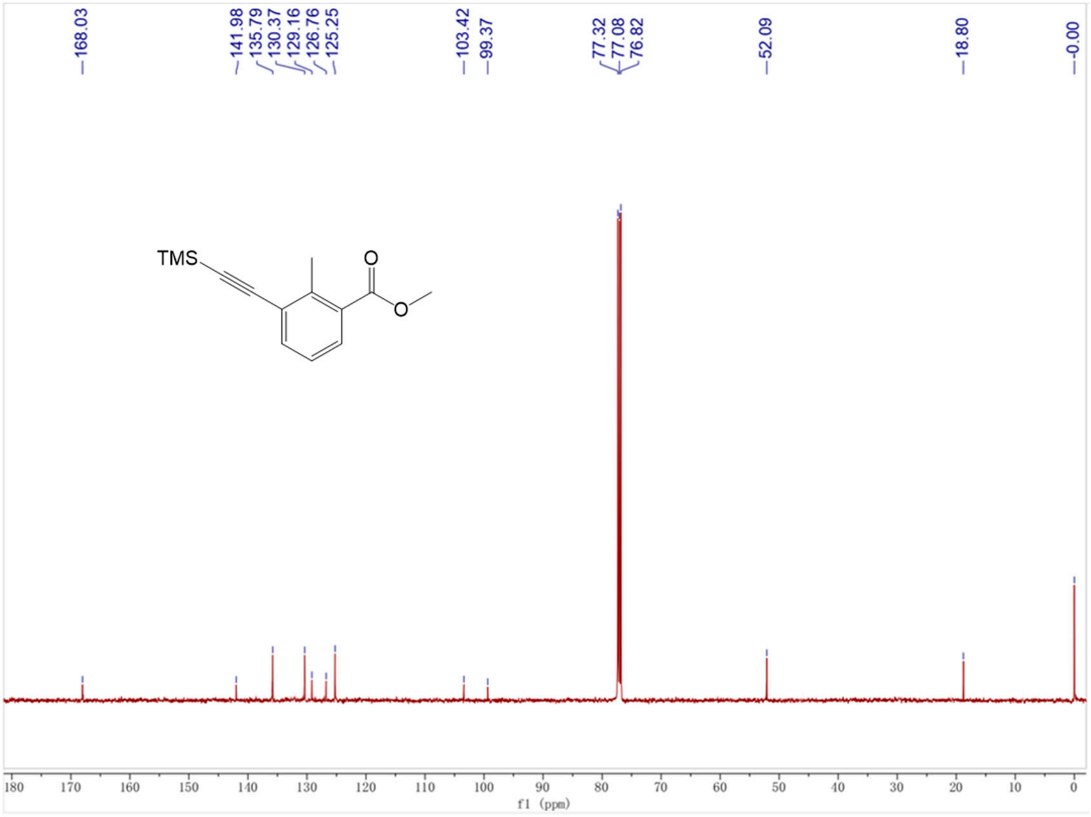

methyl 3-ethynyl-2-methylbenzoate (4)

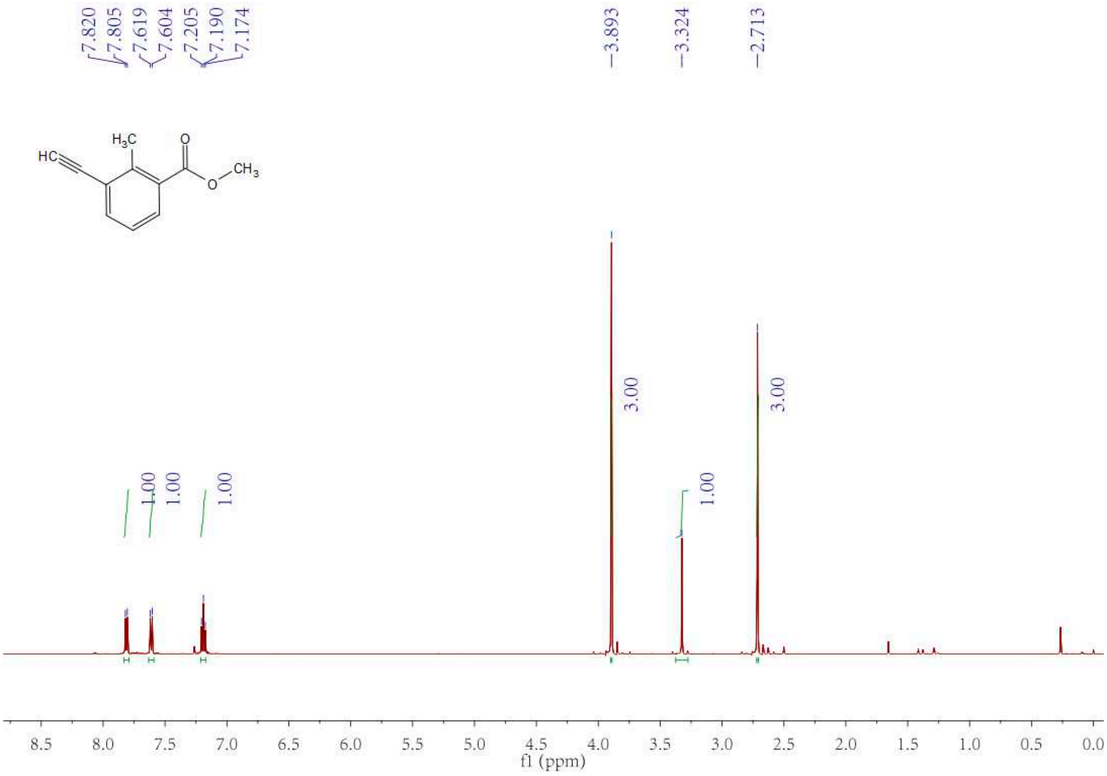

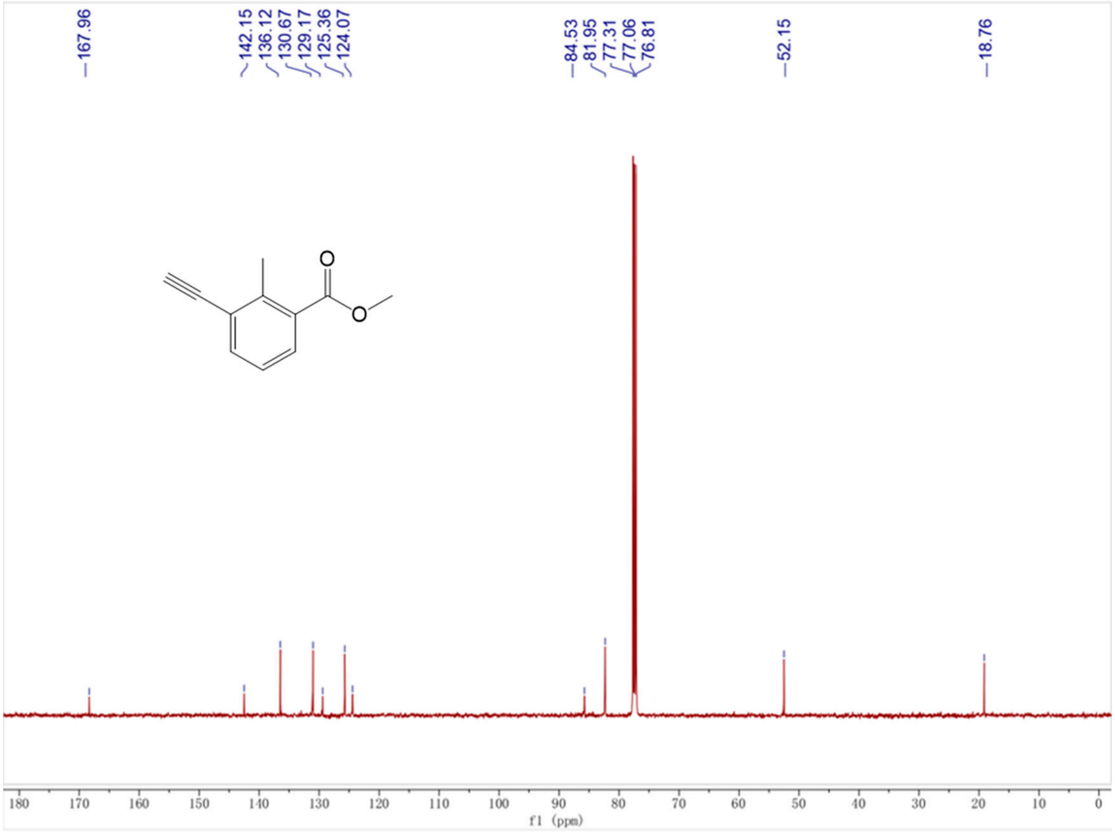

methyl 2-methyl-3-(1,2-dicarba-closo-dodecarboranyl)benzoate (5)

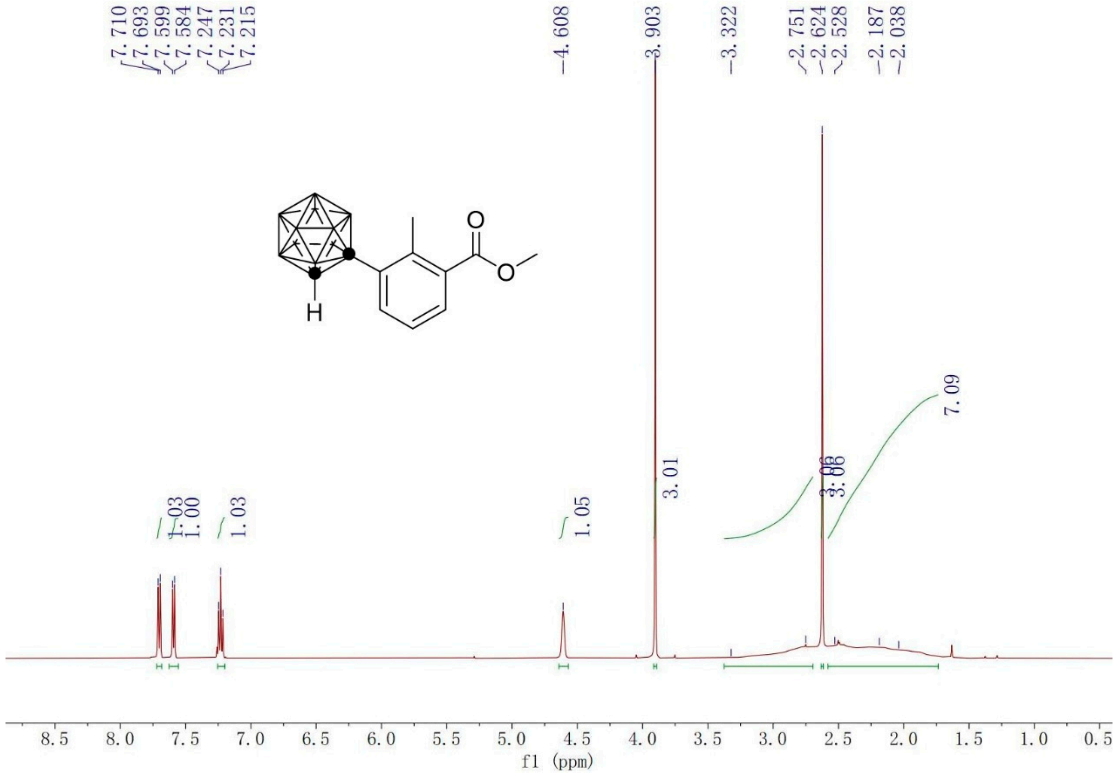

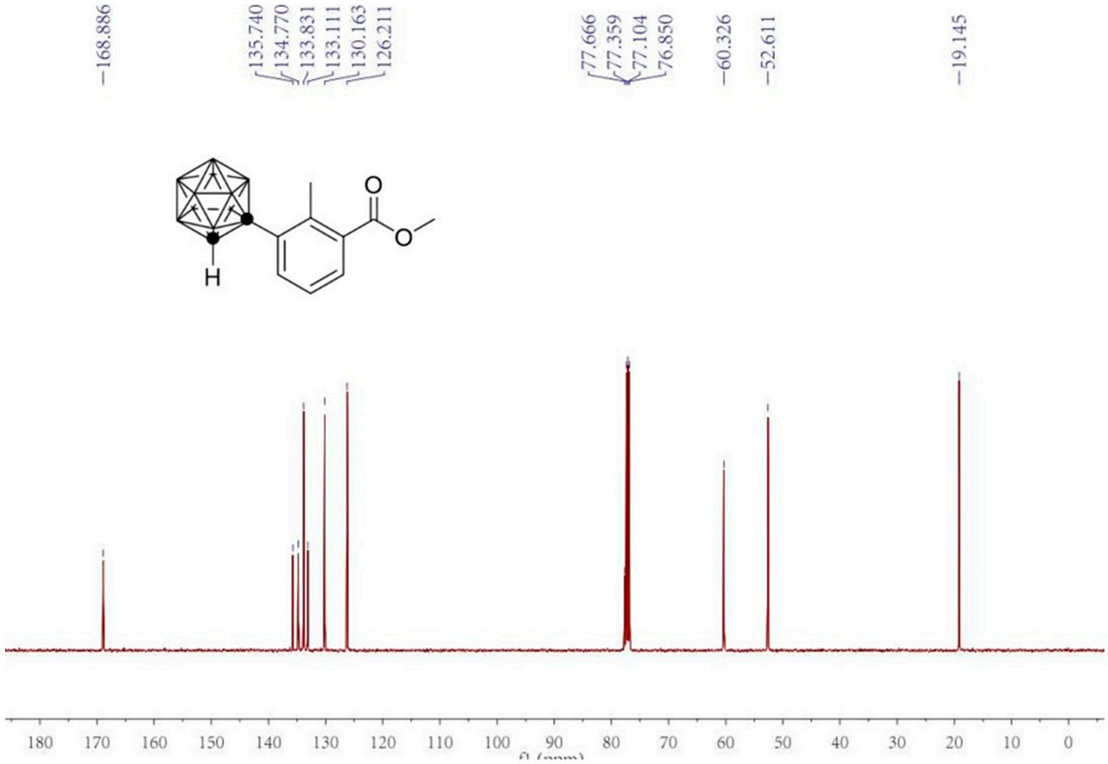

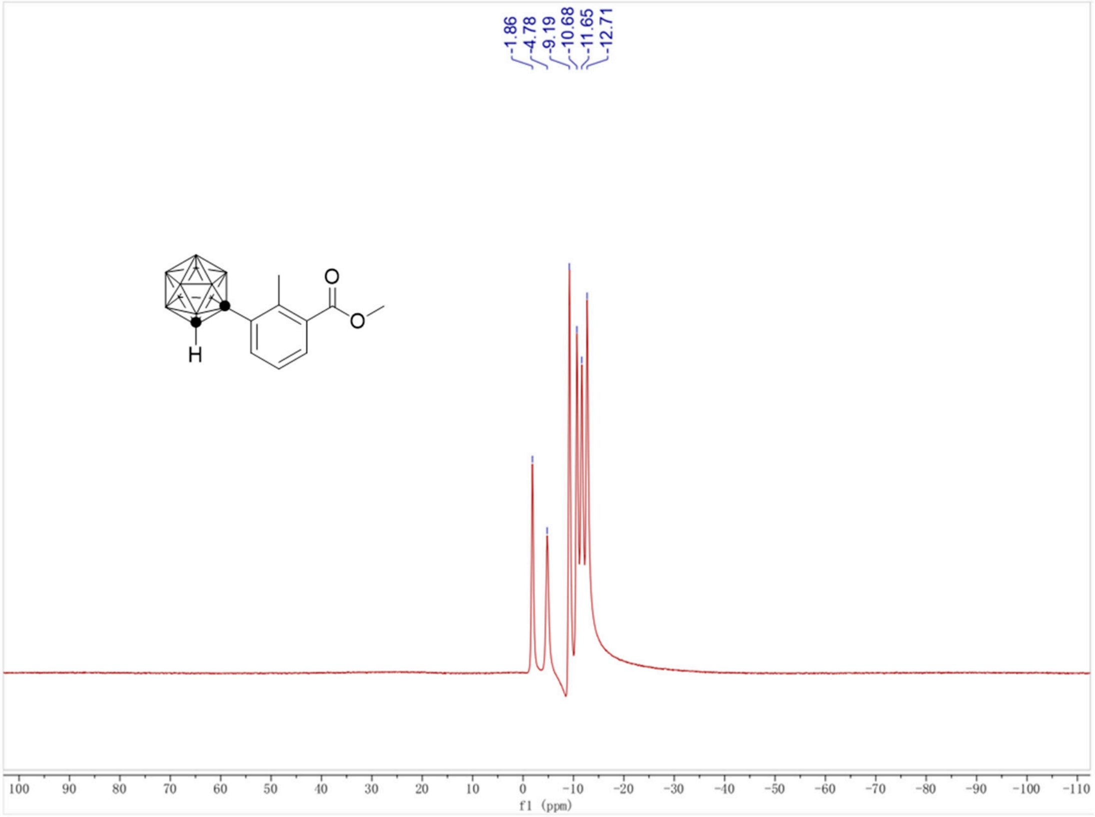

(2-methyl-3-(1,2-dicarba-closo-dodecarboranyl)phenyl)methanol (6)

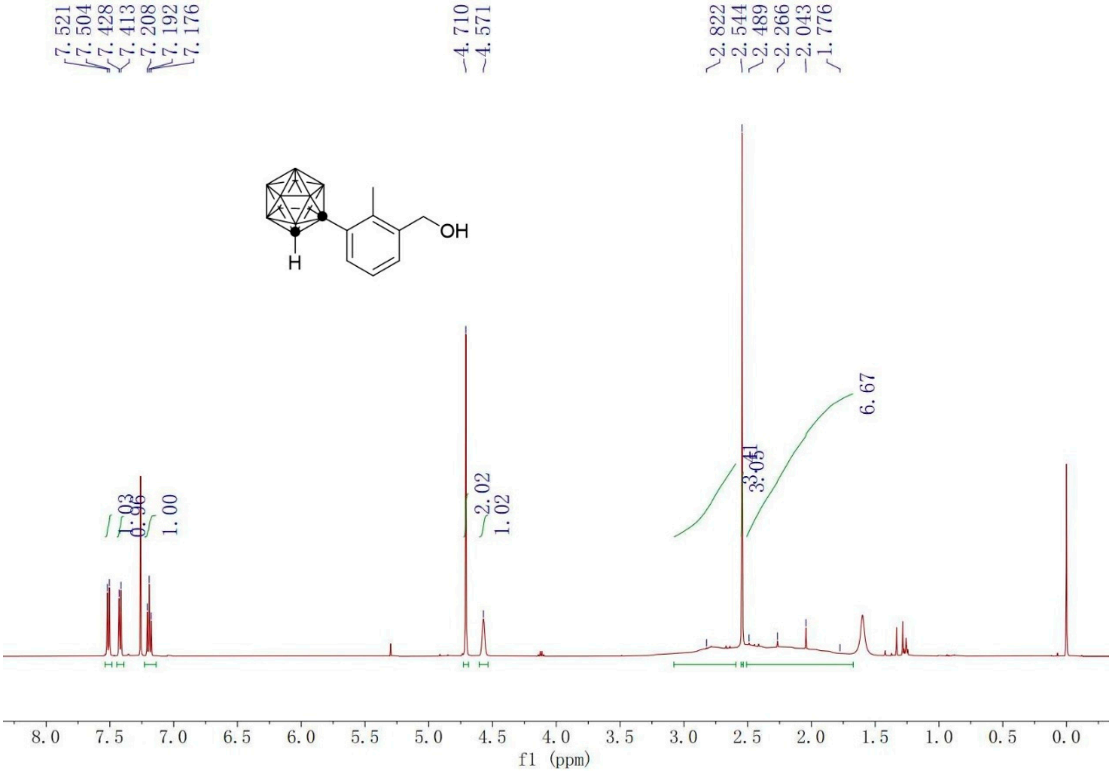

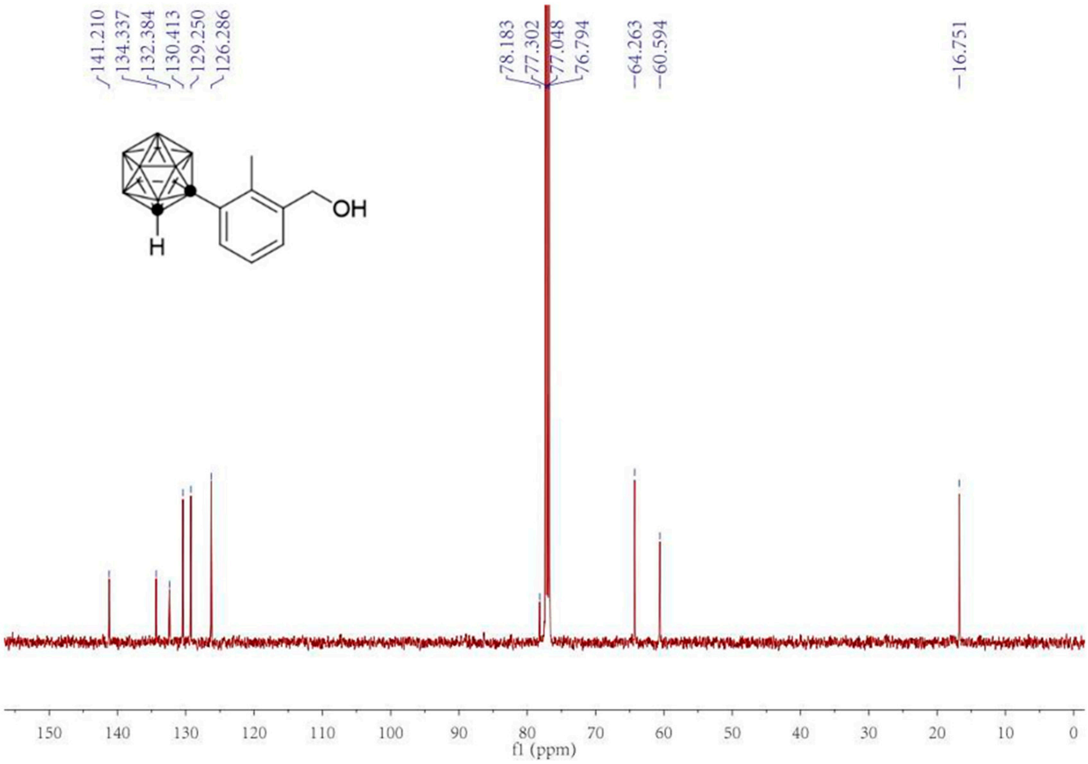
141.210
134.337
132.384
130.413
129.250
126.286
78.183
77.302
77.048
76.794
64.263
60.594
16.751
OH
H
150
140
130
120
110
100
90
80
70
60
50
40
30
20
10
0
f1 (ppm)

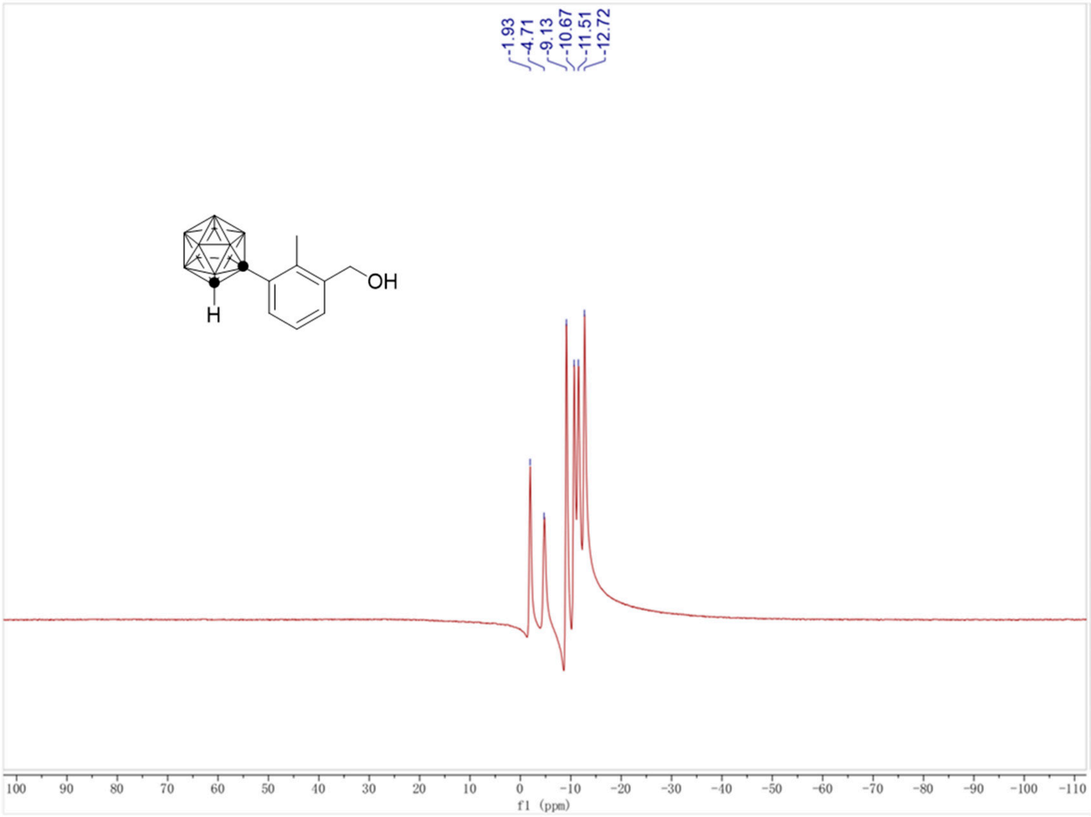
-1.93
-4.71
-9.13
-10.67
-11.51
-12.72
OH
H
100
90
80
70
60
50
40
30
20
10
0
-10
-20
-30
-40
-50
-60
-70
-80
-90
-100
-110
f1 (ppm)

2-methoxy-6-((2-methyl-3-(1,2-dicarba-closo-dodecarboranyl)benzyl)oxy) nicotinaldehyde (7)

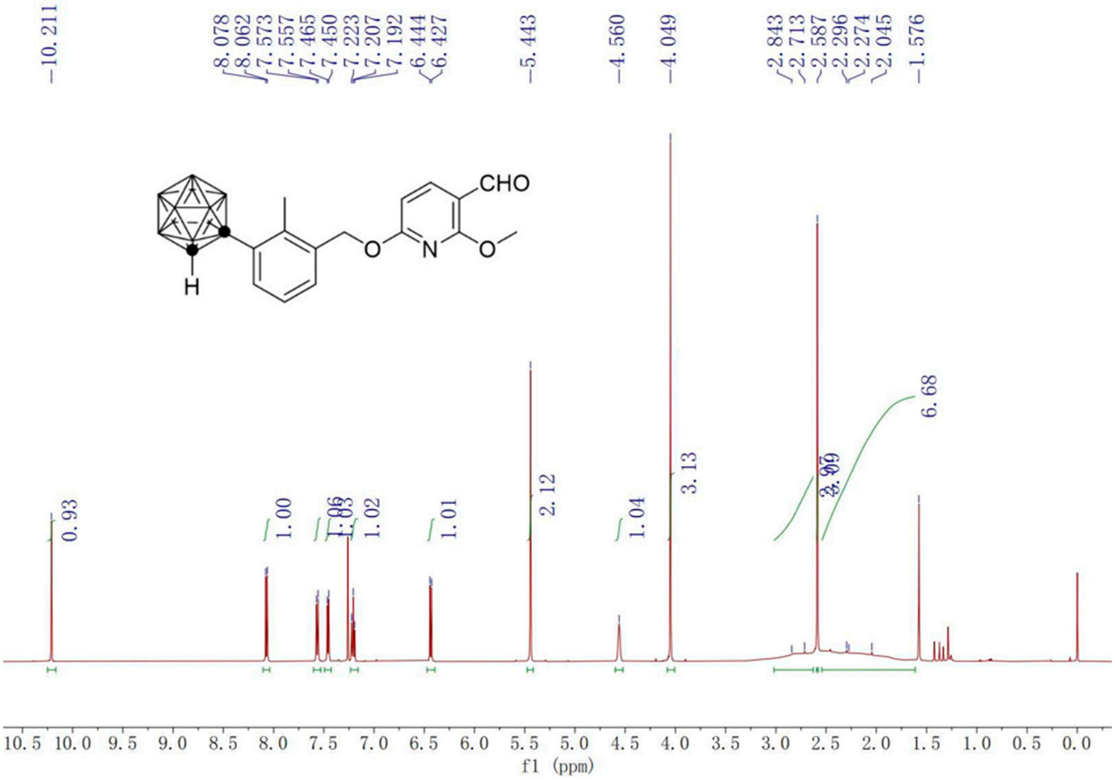

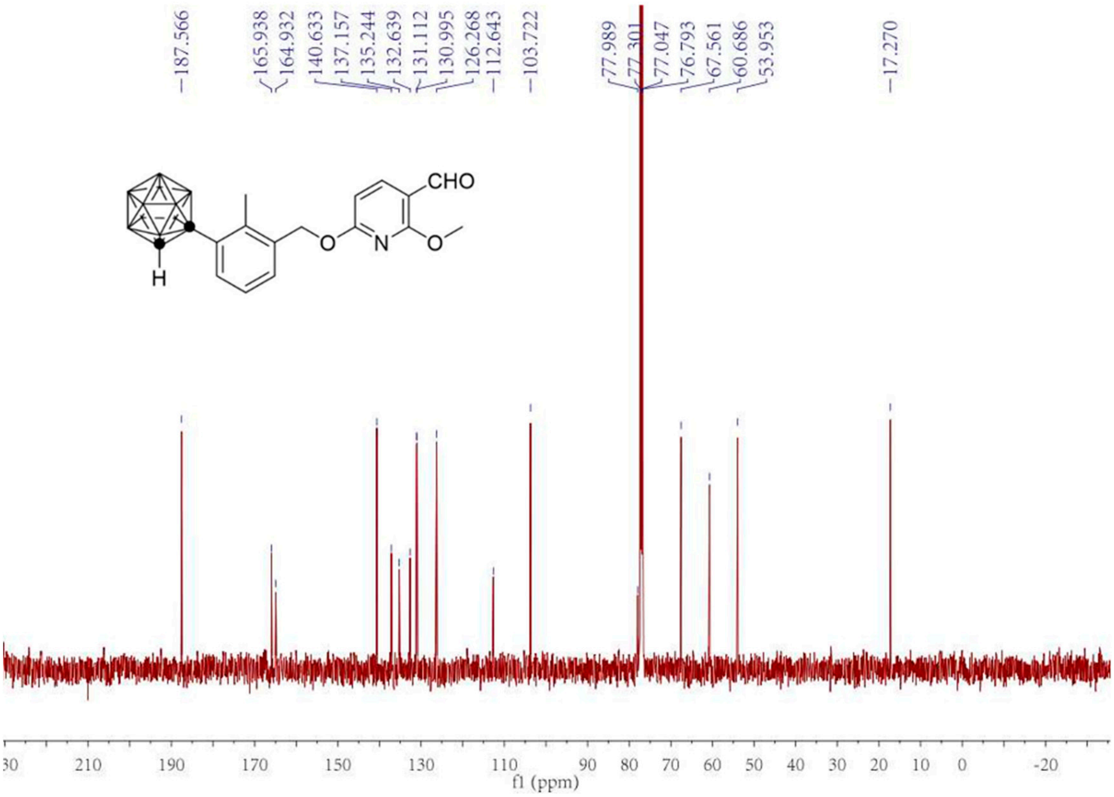

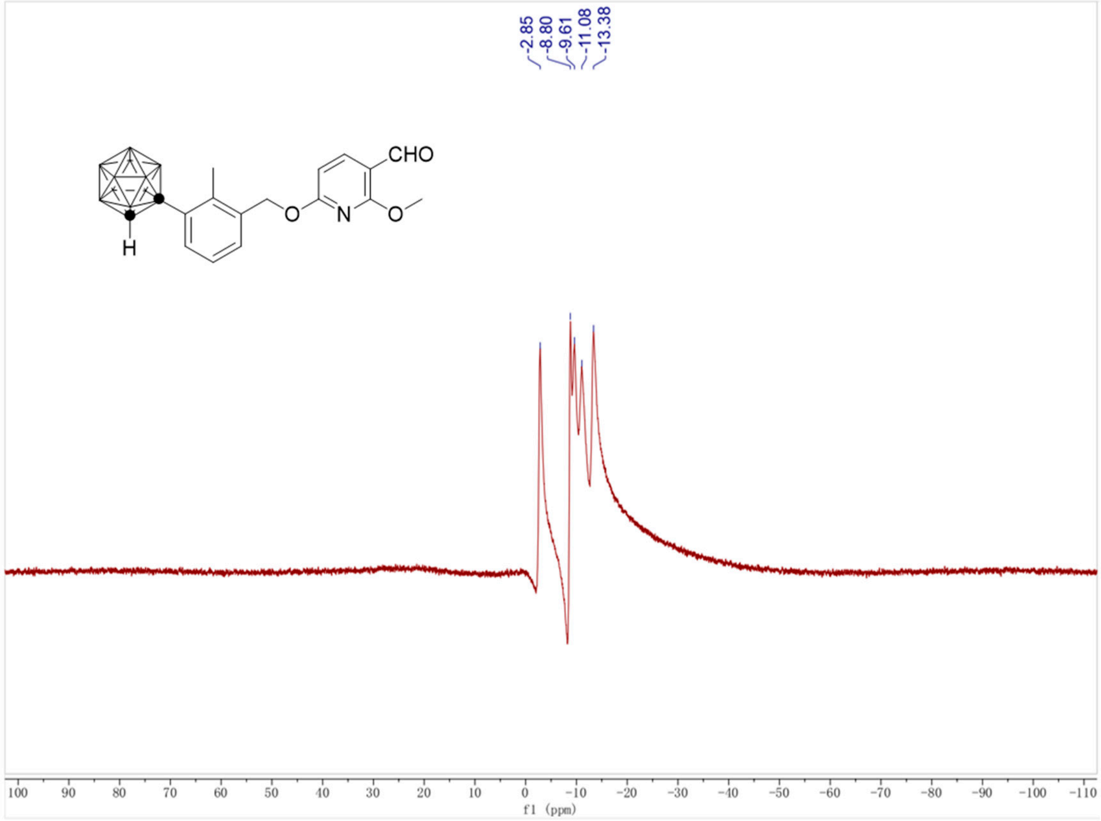

***N*-(2-(((6-((3-(1,2-dicarba-closo-dodecarboranyl)-2-methylbenzyl)oxy)-2-methoxypyridin-3-yl)methyl)amino)ethyl)acetamide (1a)**

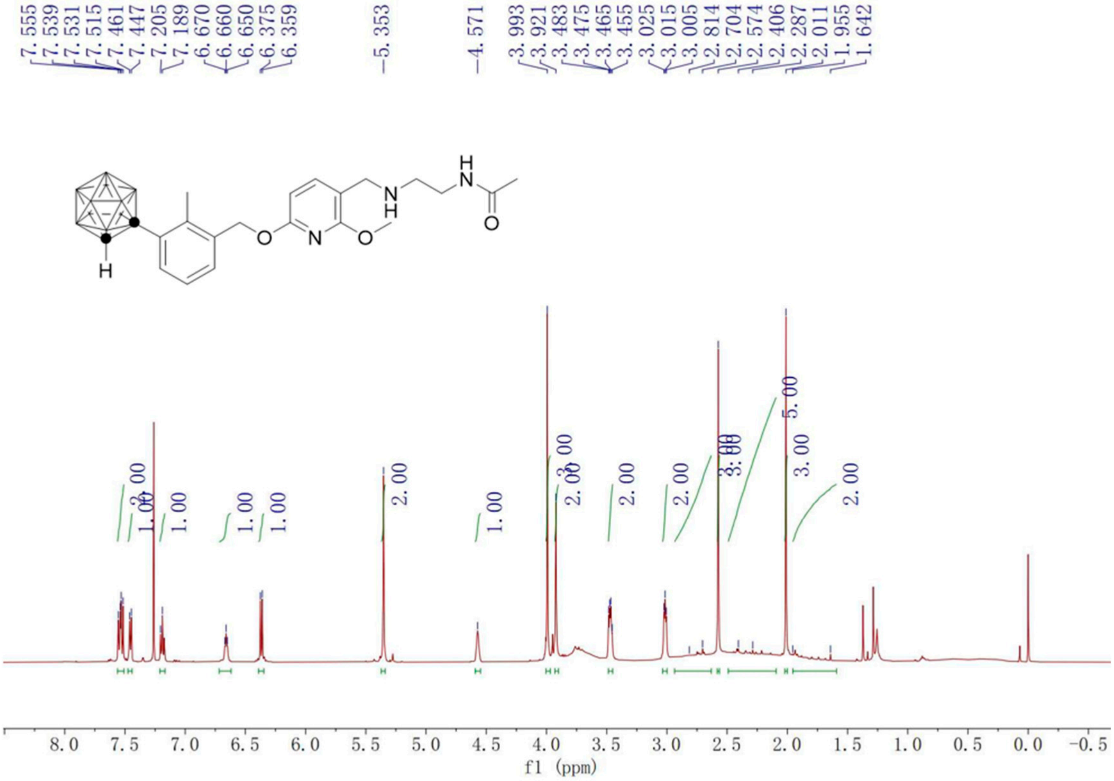

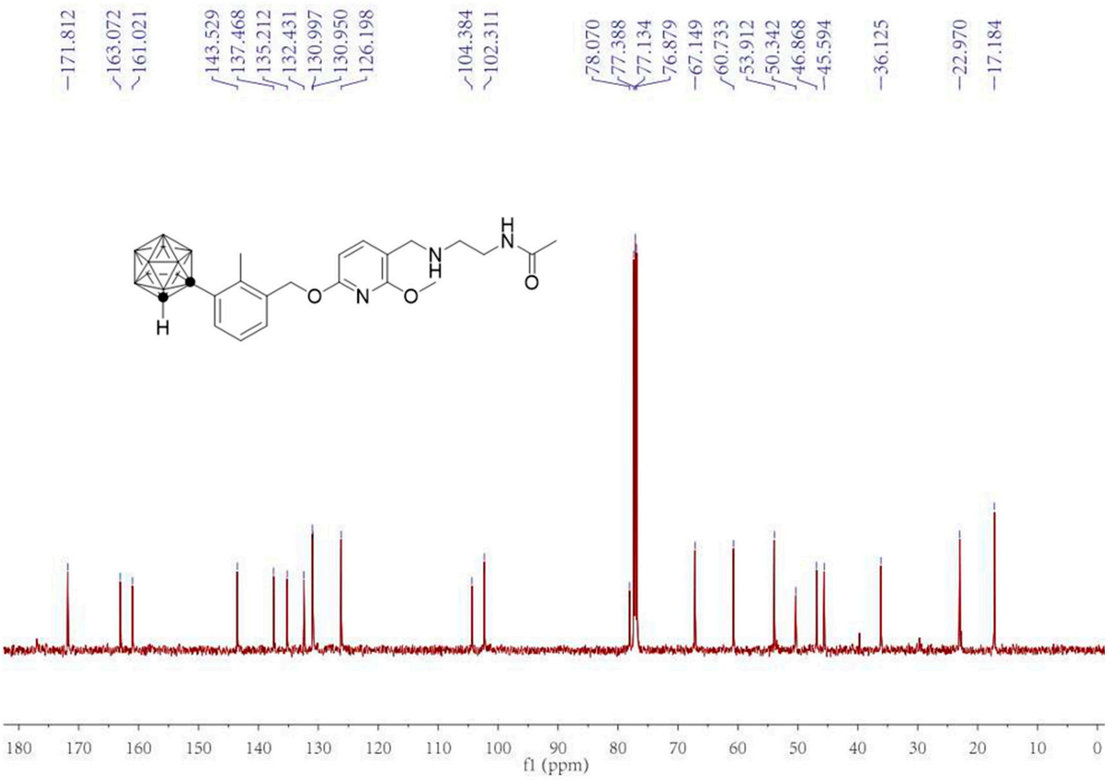
171.812
163.072
161.021
143.529
137.468
135.212
132.431
130.997
130.950
126.198
104.384
102.311
78.070
77.388
77.134
76.879
67.149
60.733
53.912
50.342
46.868
45.594
36.125
22.970
17.184
H
N
O
f1 (ppm)

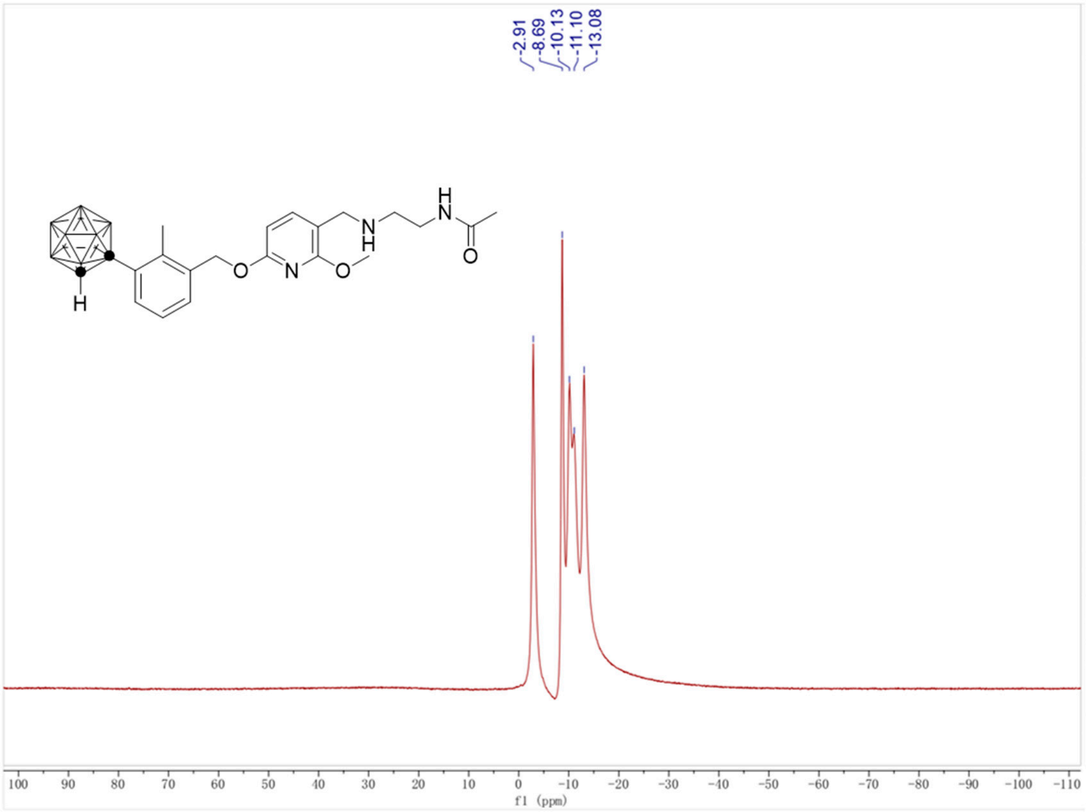
-2.91
-8.69
-10.13
-11.10
-13.08
H
N
O
f1 (ppm)

(6-((3-(1,2-dicarba-closo-dodecarboranyl)-2-methylbenzyl)oxy)-2-methoxypyridin-3-yl)methanol (1b)

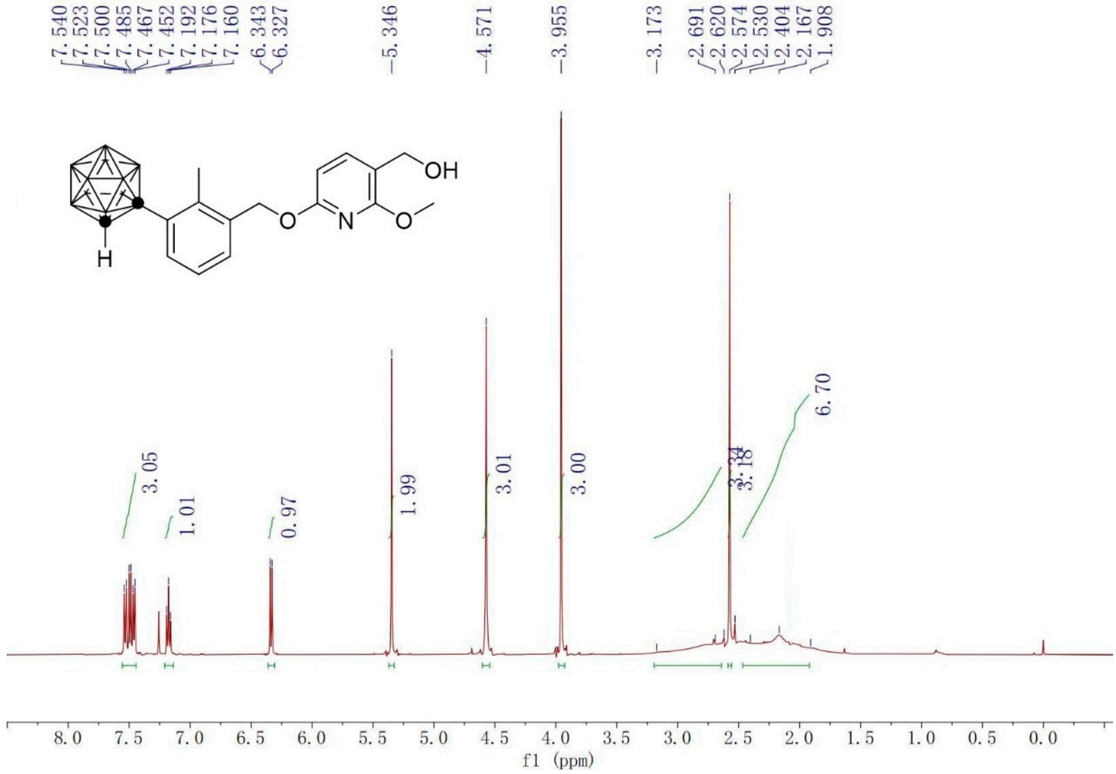

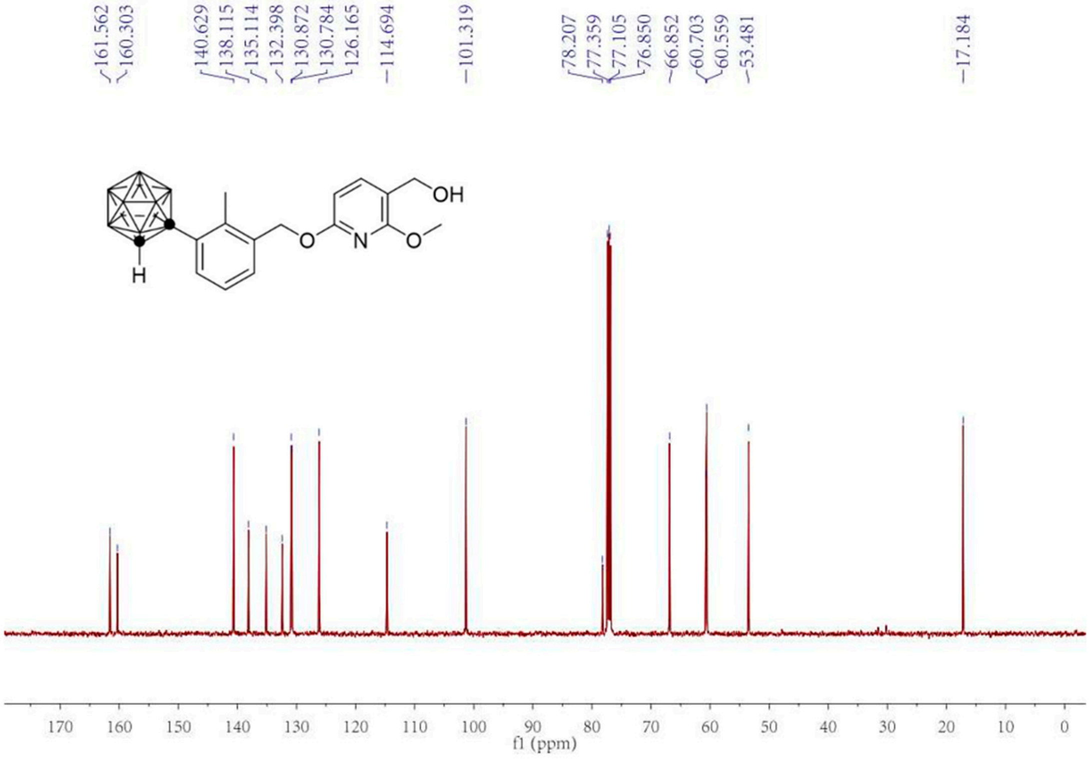

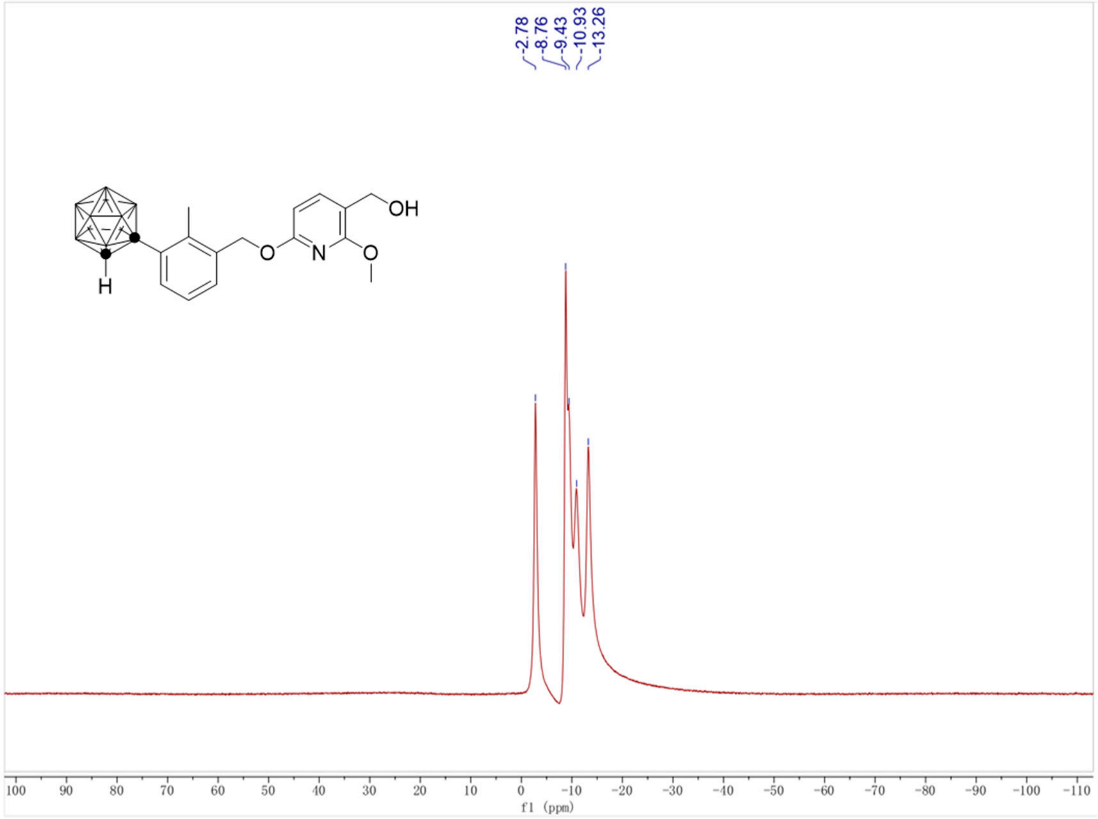

tert-butyl 2-((6-((3-(1,2-dicarba-closo-dodecarboranyl)-2-methylbenzyl)oxy)-2-methoxypyridin-3-yl)methoxy)acetate (8)

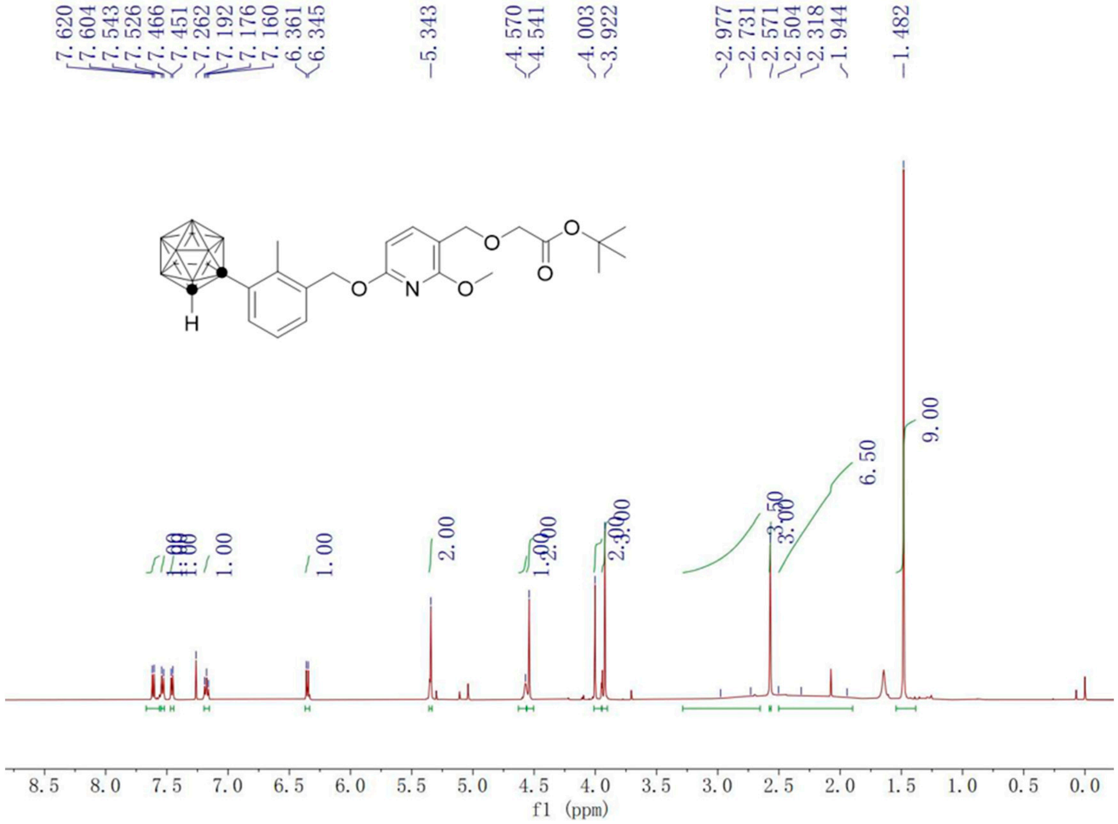

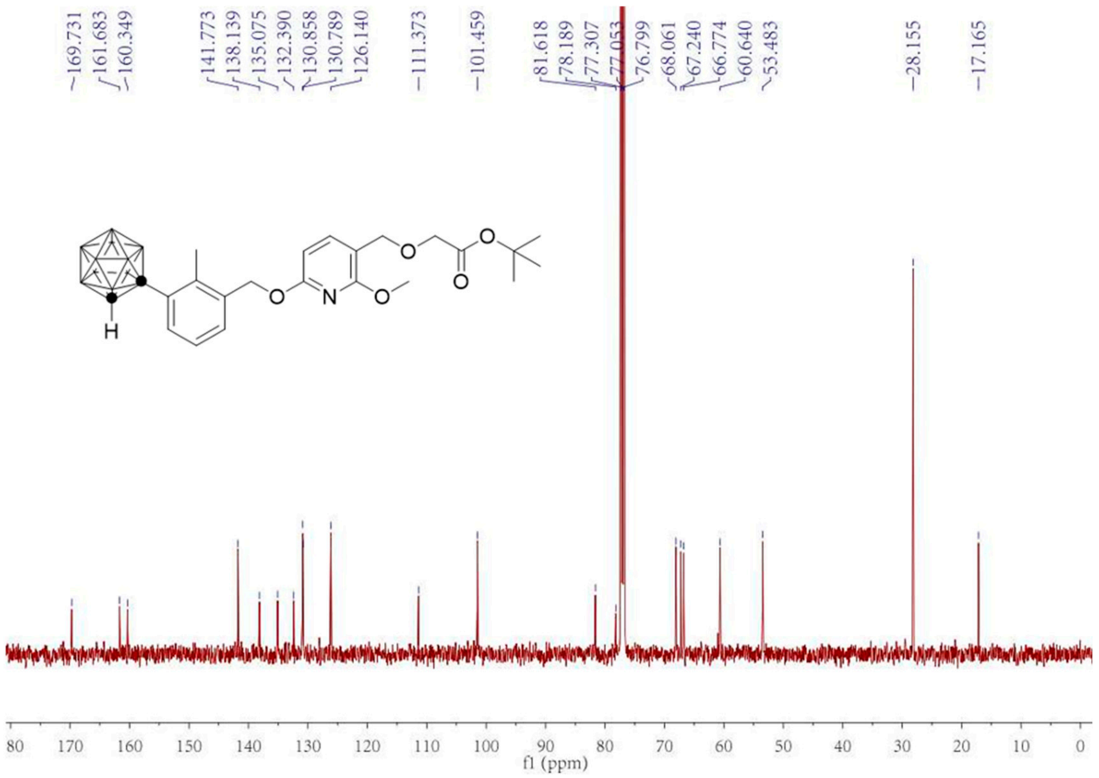
169.731
161.683
160.349
141.773
138.139
135.075
132.390
130.858
130.789
126.140
111.373
101.459
81.618
78.189
77.307
77.053
76.799
68.061
67.240
66.774
60.640
53.483
28.155
17.165
H
N
O
f1 (ppm)

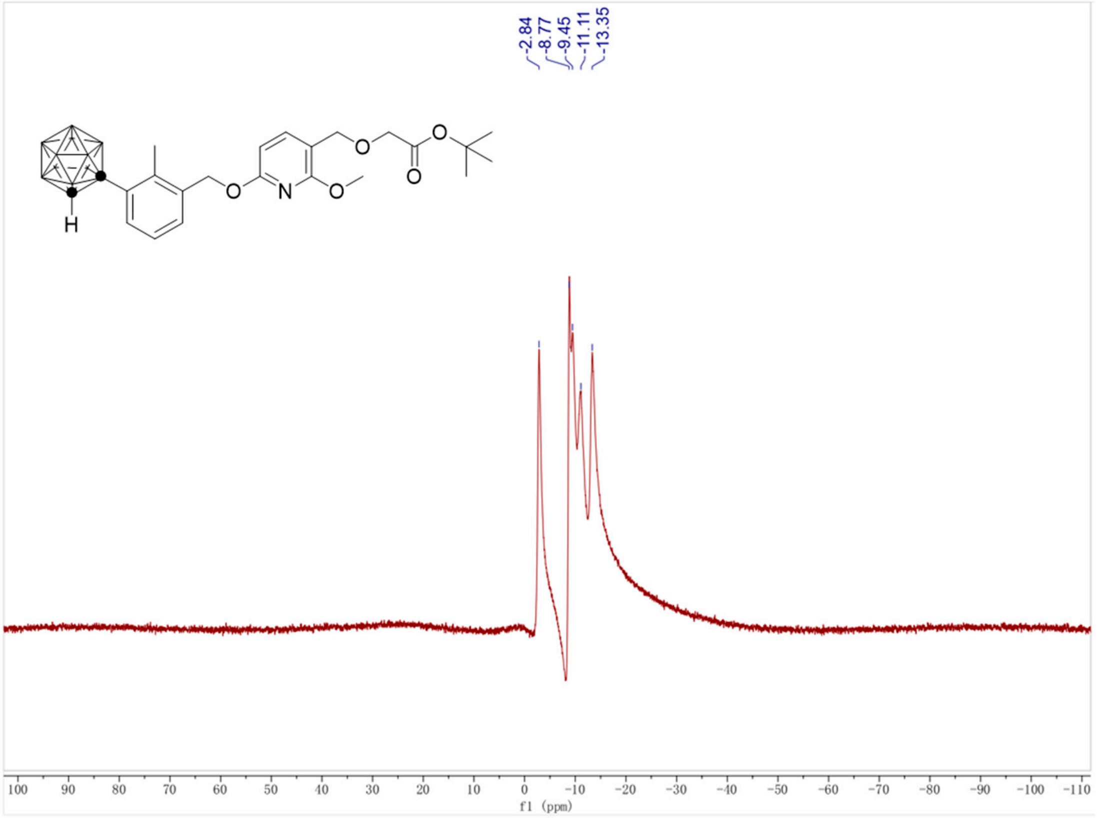
-2.84
-8.77
-9.45
-11.11
-13.35
H
N
O
f1 (ppm)

2-((6-((3-(1,2-dicarba-closo-dodecarboranyl)-2-methylbenzyl)oxy)-2-methoxypyridin-3-yl)methoxy)acetic acid (1c)

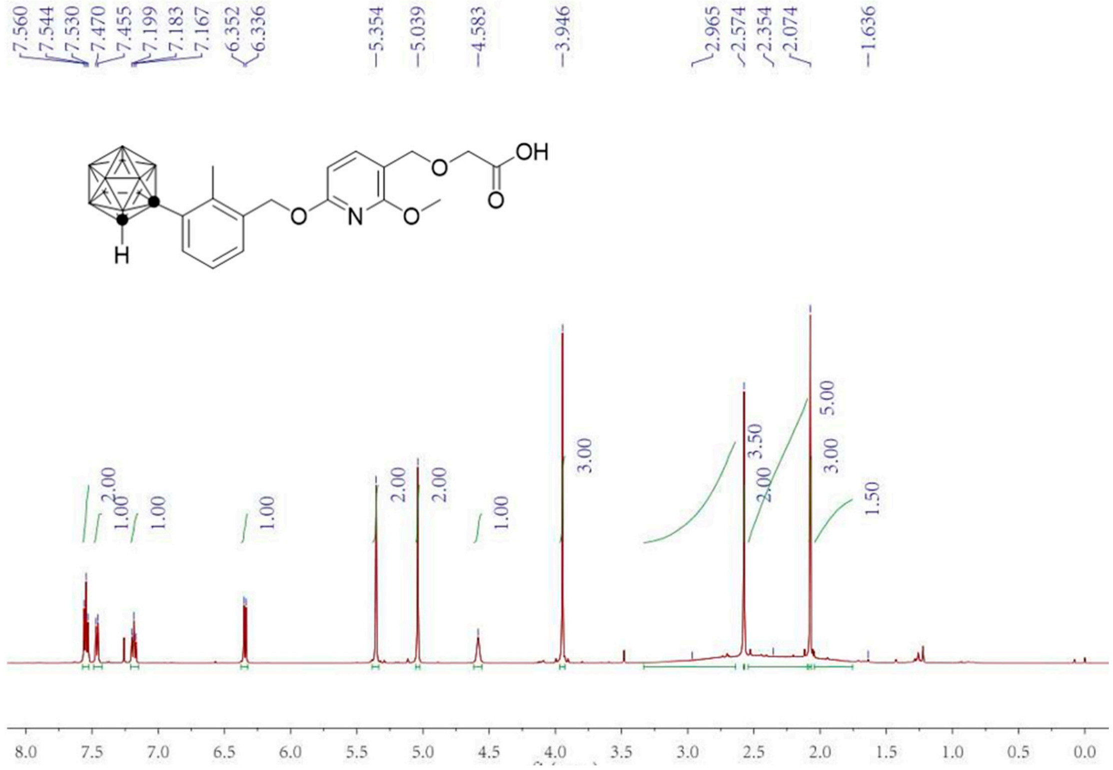

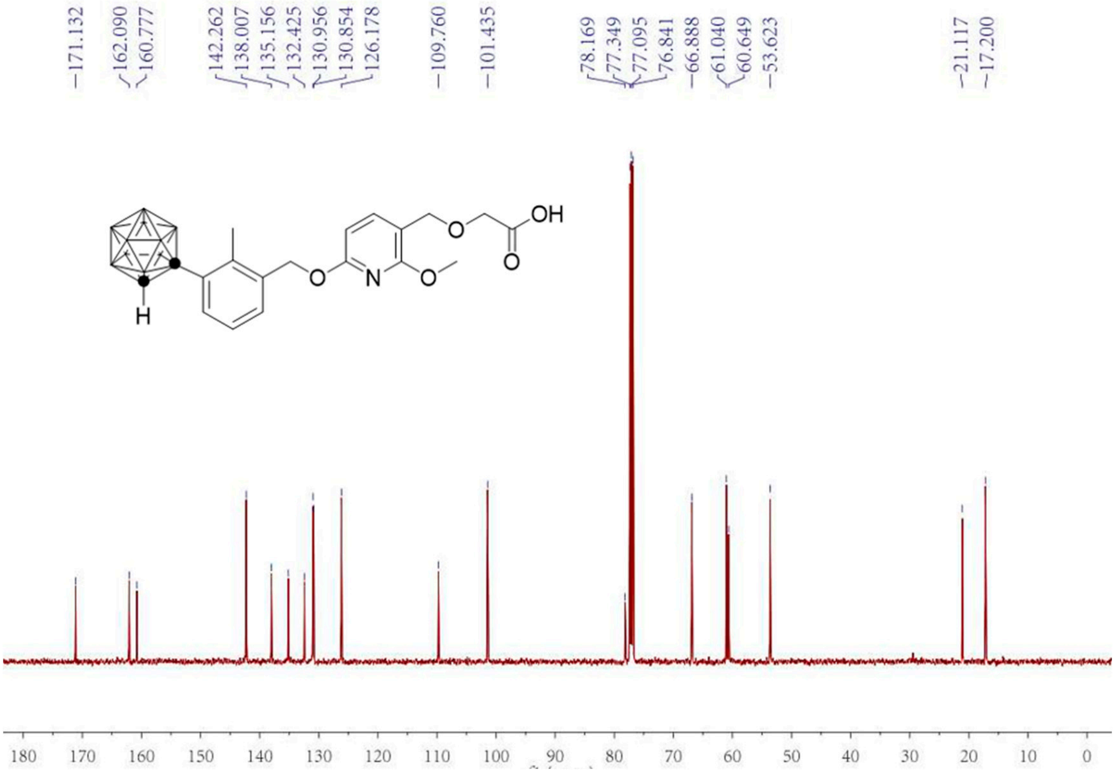

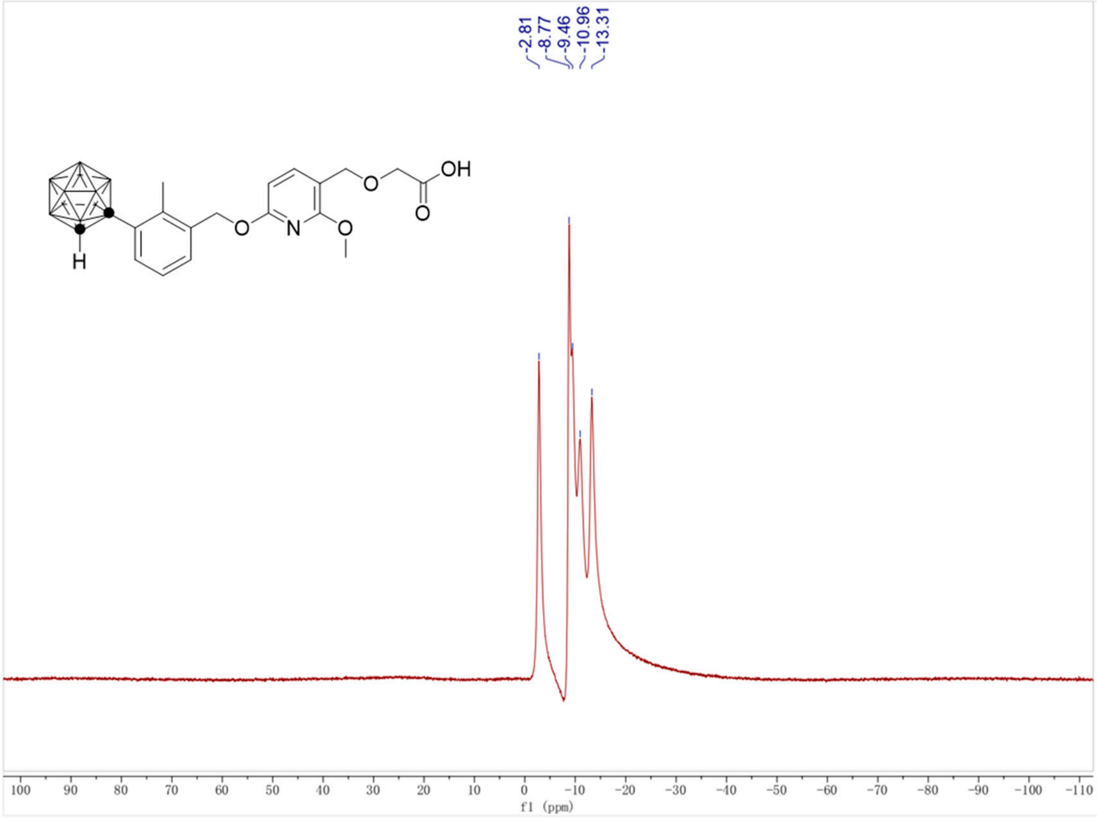
-2.81
-8.77
-9.46
-10.96
-13.31
OH
O
O
O
N
O
O
H
f1 (ppm)

High-resolution mass spectra

Compound 1a: HRMS (ESI positive mode, m/z) calcd for $C_{21}H_{36}B_{10}N_3O_3$ $[M + H]^+$: 487.3718, found: 487.3724.

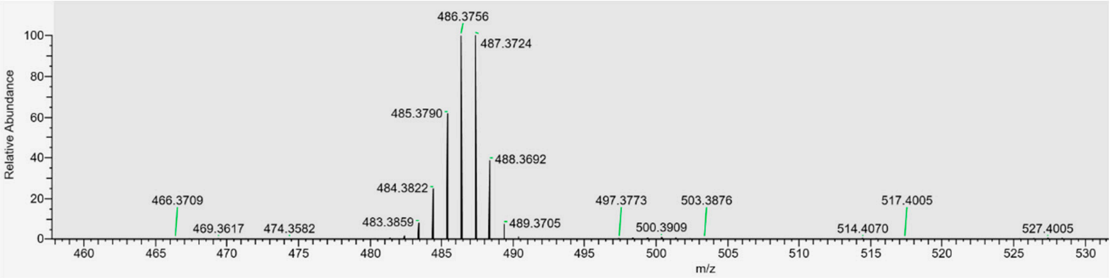

Compound 1b: HRMS (ESI positive mode, m/z) calcd for $C_{17}H_{28}B_{10}NO_3$ $[M + H]^+$: 403.3031, found: 403.3030.

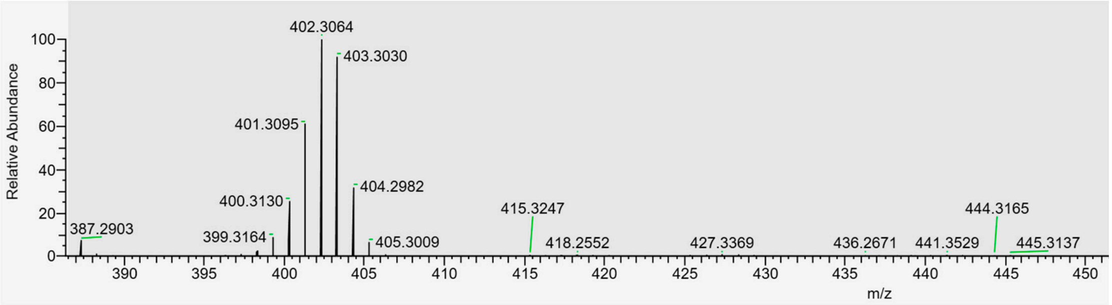

Compound 1c: HRMS (ESI negative mode, m/z) calcd for $C_{19}H_{28}B_{10}NO_5$ $[M - H]^-$: 459.2940, found: 459.2934.

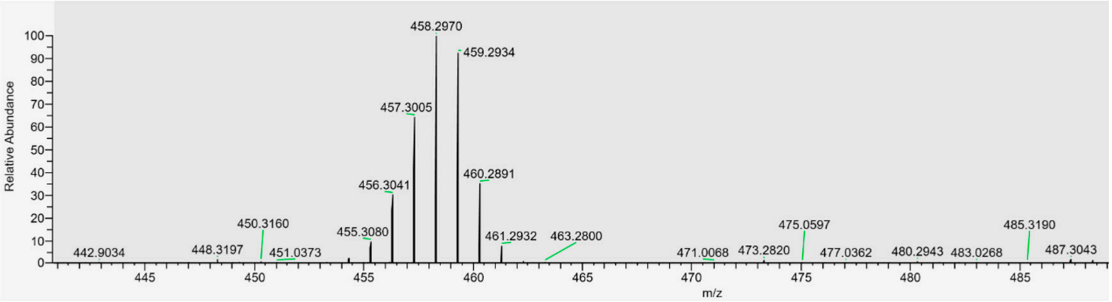

HPLC data of final compounds

HPLC Spectra of compound **1a**

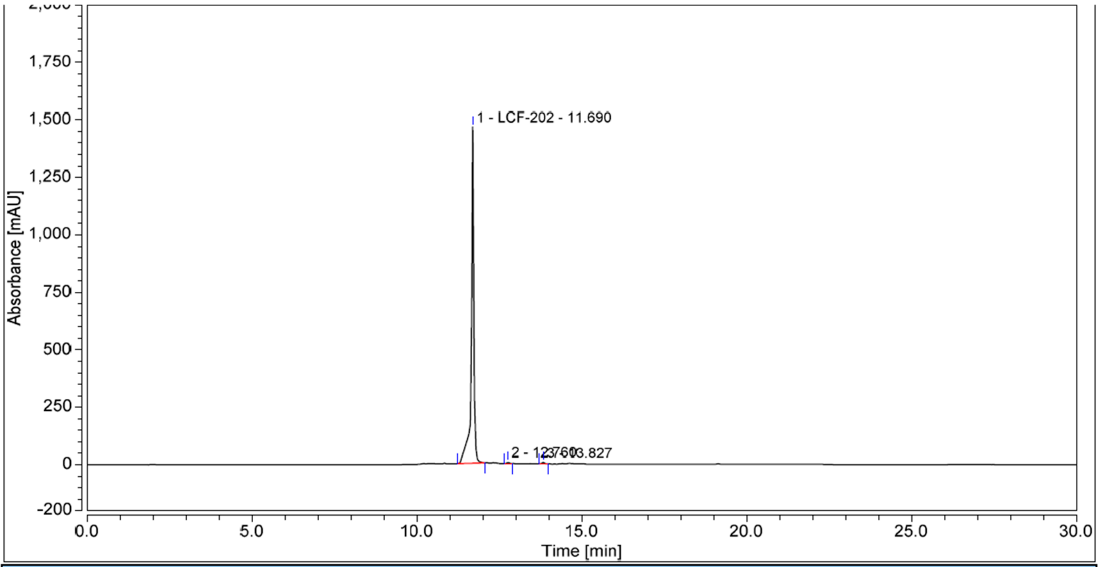

Integration Results

No.	Peak Name	Retention Time min	Area mAU*min	Height mAU	Relative Area %	Relative Height %
1	LCF-202	11.690	126.273	1463.773	99.25	98.97
2		12.760	0.392	7.891	0.31	0.53
3		13.827	0.568	7.294	0.45	0.49
**Total:**			**127.233**	**1478.957**	**100.00**	**100.00**

HPLC Spectra of compound **1b**

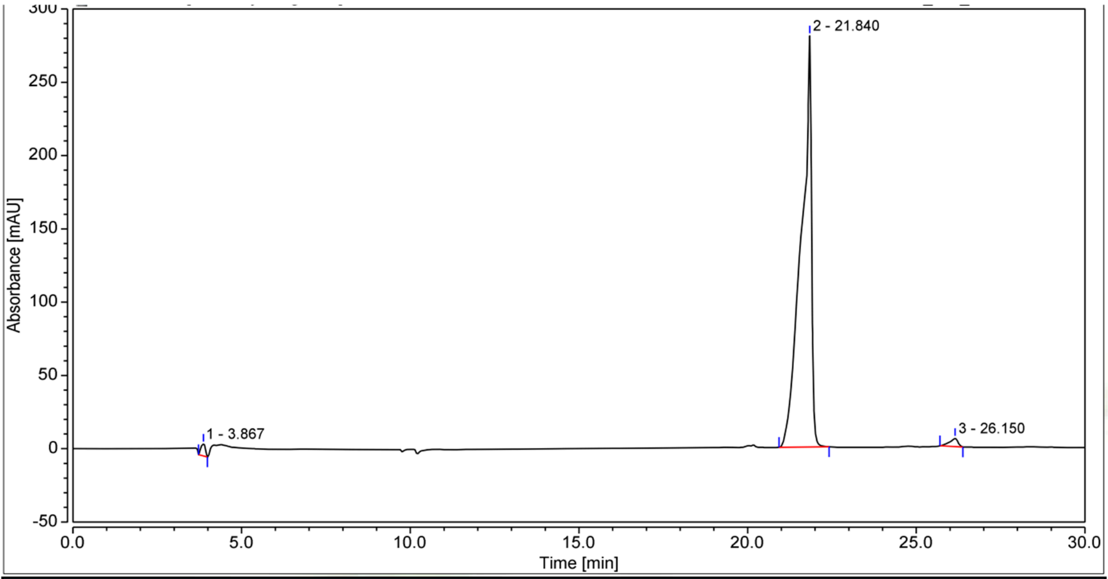

Integration Results

No.	Peak Name	Retention Time min	Area mAU*min	Height mAU	Relative Area %	Relative Height %	Amount n.a.
1		3.867	1.254	8.108	1.22	2.76	n.a.
2		21.840	99.561	280.492	97.20	95.33	n.a.
3		26.150	1.614	5.619	1.58	1.91	n.a.
**Total:**			**102.429**	**294.219**	**100.00**	**100.00**	

HPLC Spectra of compound **1c**

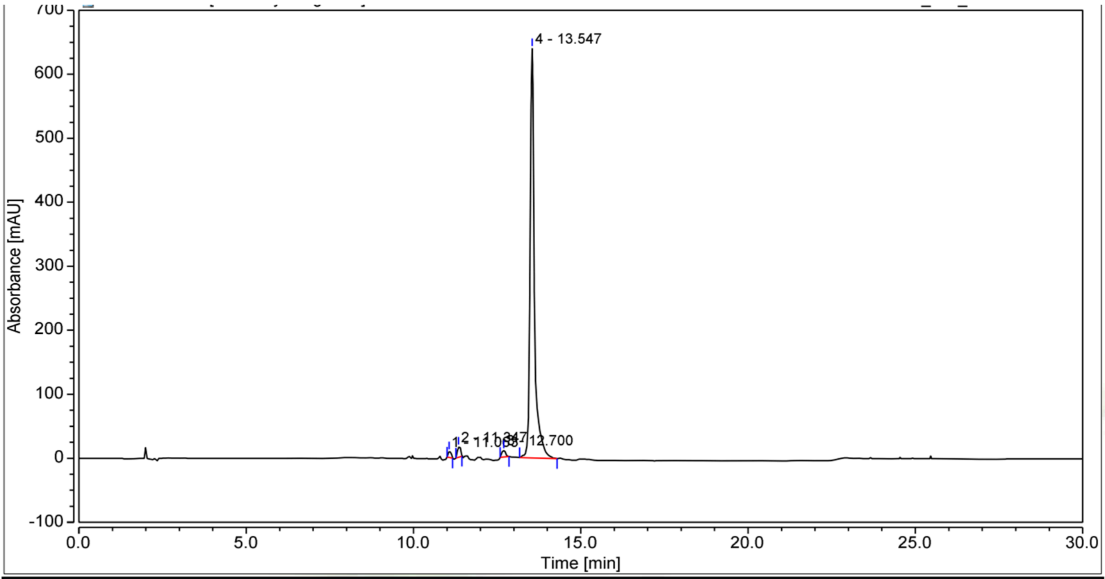

Integration Results							
No.	Peak Name	Retention Time min	Area mAU*min	Height mAU	Relative Area %	Relative Height %	Amount n.a.
1		11.063	1.035	8.972	1.03	1.33	n.a.
2		11.347	1.925	15.753	1.91	2.34	n.a.
3		12.700	1.389	10.012	1.38	1.48	n.a.
4		13.547	96.377	639.713	95.68	94.85	n.a.
**Total:**			**100.726**	**674.451**	**100.00**	**100.00**	

Determination of X-ray crystallographic structure of compound 1a

Single crystals of **1a** suitable for X-ray analysis were grown in dichloromethane/petroleum ether at room temperature. The ellipsoid contour was set at 50% probability levels in the caption for the ORTEP diagram. Crystallographic data have been deposited with the Cambridge Crystallographic Data Centre as supplementary publication nos. CCDC 2342434. Copies of these data can be obtained free of charge from the Cambridge Crystallographic Data Centre via www.ccdc.cam.ac.uk/data_request/cif.

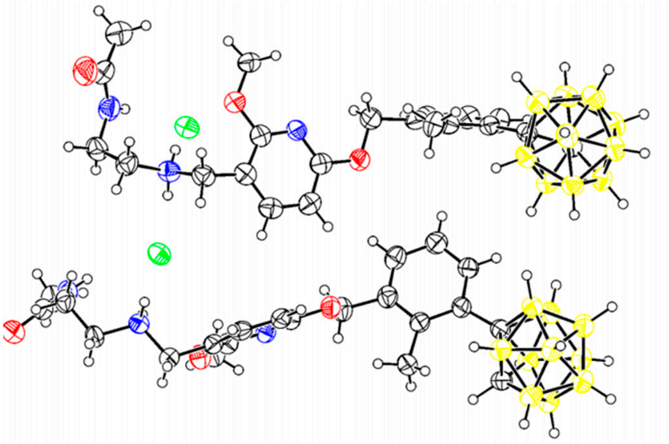

Table S2. Crystal data and structure refinement for compound 1a

Identification code	cu_20231657_0m_4_sq
Empirical formula	$C_{21}H_{36}B_{10}ClN_3O_3$
Formula weight	522.08
Temperature/K	100
Crystal system	monoclinic
Space group	$P2_1/n$
a/Å	7.0585(2)
b/Å	30.3949(9)
c/Å	29.2410(8)
α/°	90
β/°	91.434(2)
γ/°	90
Volume/Å^3	6271.5(3)
Z	8
ρ_{calc}g/cm^3	1.106
μ/mm^{-1}	1.275
F(000)	2192.0
Crystal size/mm^3	0.07 × 0.04 × 0.02
Radiation	CuKα (λ = 1.54178)

2Θ range for data collection/°	4.194 to 127.482
Index ranges	? ≤ h ≤ ?, ? ≤ k ≤ ?, ? ≤ l ≤ ?
Reflections collected	10101
Independent reflections	10101 [R_{int} = ?, R_{sigma} = 0.0983]
Data/restraints/parameters	10101/2/699
Goodness-of-fit on F^2	1.035
Final R indexes [I>=2σ (I)]	R_1 = 0.0995, wR_2 = 0.2588
Final R indexes [all data]	R_1 = 0.1325, wR_2 = 0.2840
Largest diff. peak/hole / e Å^{-3}	0.34/-0.46

Table S3 Fractional Atomic Coordinates (×104) and Equivalent Isotropic Displacement Parameters (Å2×103) for cu_20231657_0m_4_sq. Ueq is defined as 1/3 of the trace of the orthogonalised UIJ tensor.

Atom	*x*	*y*	*z*	U(eq)
Cl1	7149.3(19)	4370.6(4)	8687.4(4)	50.8(3)
Cl2	2111.1(19)	5095.6(4)	8269.8(4)	51.3(3)
O1	8340(5)	3450.4(11)	6963.9(11)	47.3(8)
O2	9431(5)	3345.5(12)	8526.1(11)	50.1(9)
O5	4247(5)	5595.2(12)	7395.8(12)	51.1(9)
O4	3116(5)	4400.3(11)	6445.8(12)	49.1(9)
O3	10370(6)	4275.6(13)	10342.3(13)	60.7(10)
N1	8834(6)	3390.4(14)	7744.2(14)	43.4(10)
N4	3607(6)	4997.7(14)	6931.8(13)	45.4(10)
N2	11609(6)	4231.3(14)	8798.9(13)	42.2(9)
N5	6613(6)	5271.8(14)	8218.9(14)	47.2(10)
N3	9541(7)	4501.0(15)	9629.9(15)	48.3(10)
O6	5469(8)	6544.3(16)	8744.7(16)	83.5(15)
N6	4339(7)	5858.1(17)	8791.1(16)	57.7(12)
C11	9365(8)	3554.9(17)	7350.4(16)	44.9(12)
C18	12516(8)	4326.8(18)	9252.7(16)	45.5(12)
C4	6864(8)	2815.7(15)	6220.5(17)	43.8(12)
C13	11988(8)	3937.7(16)	7680.3(18)	46.7(12)
C24	801(7)	4137.8(16)	5094.4(17)	42.1(11)
C15	9890(8)	3504.1(18)	8109.2(17)	49.6(13)
C12	10931(8)	3825.9(17)	7288.4(17)	46.1(12)
C34	5787(8)	4391.8(18)	6932.7(18)	51.6(13)
C33	4166(8)	4608.1(17)	6776.9(17)	47.7(12)
C37	4721(8)	5187.9(17)	7236.7(17)	45.4(12)

Table S3 Fractional Atomic Coordinates (×104) and Equivalent Isotropic Displacement Parameters (Å2×103) for cu_20231657_0m_4_sq. Ueq is defined as 1/3 of the trace of the orthogonalised UIJ tensor.

Atom	*x*	*y*	*z*	U(eq)
C1	6764(7)	2508.3(16)	5386.4(16)	42.0(11)
C25	1589(8)	4386.7(17)	5460.5(18)	46.4(12)
C22	1731(7)	4092.0(16)	4624.9(17)	42.7(11)
C3	5976(8)	2760.8(16)	5781.8(17)	45.3(12)
C9	4240(7)	2965.8(16)	5688.6(17)	43.8(11)
C14	11499(7)	3772.9(16)	8103.2(17)	43.3(11)
C38	2530(8)	5785.2(18)	7218.8(19)	53.1(13)
C36	6420(8)	5013.2(18)	7419.2(18)	48.6(12)
C20	9161(8)	4295.1(18)	10030.5(18)	49.1(13)
C6	5929(8)	3073.3(17)	6540.3(17)	47.7(12)
C27	664(8)	4374.2(16)	5880.5(18)	47.1(12)
C17	12565(8)	3876.5(18)	8540.1(17)	47.7(12)
C7	4212(8)	3273.8(18)	6435.8(19)	53.1(13)
C32	1412(9)	4622.7(18)	6289.1(18)	52.2(13)
C2	8323(7)	2108.1(16)	5470.0(18)	44.1(11)
C35	6910(8)	4601.8(18)	7264.5(18)	51.8(13)
C30	-919(8)	3917.2(18)	5147.0(19)	50.7(13)
C8	3340(8)	3220.8(19)	6004.5(19)	55.0(14)
C19	11381(8)	4663.9(18)	9515.5(18)	50.2(13)
C23	906(8)	3691.6(18)	4284.8(17)	47.9(12)
C39	7546(8)	5267(2)	7767.2(18)	52.9(13)
C10	6728(8)	3156.1(19)	7019.0(18)	53.4(13)
C28	-988(9)	4137.6(18)	5927.8(19)	53.9(14)
C26	3342(8)	4671.3(19)	5431.3(19)	53.6(14)
C40	7427(9)	5599(2)	8553(2)	57.9(14)
C5	8737(9)	2610.8(19)	6374.9(19)	56.7(14)
C29	-1797(9)	3909.2(19)	5559.6(19)	54.5(13)
C21	7215(9)	4111(2)	10067(2)	60.7(15)
C16	7716(9)	3096(2)	8551(2)	59.5(15)
C41	6129(9)	5662(2)	8942(2)	59.0(15)
C42	4125(12)	6283(2)	8694(2)	72.0(19)
B3	8086(8)	1696.5(19)	5082.2(19)	43.3(13)
B4	6081(9)	1959.1(19)	5306(2)	44.5(13)
B1	9112(9)	2603.4(19)	5241(2)	45.6(13)
B9	4106(9)	4149(2)	4515(2)	48.4(14)
B5	5393(9)	2375(2)	4903.4(19)	43.7(13)

Table S3 Fractional Atomic Coordinates (×104) and Equivalent Isotropic Displacement Parameters (Å2×103) for cu_20231657_0m_4_sq. Ueq is defined as 1/3 of the trace of the orthogonalised UIJ tensor.

Atom	*x*	*y*	*z*	U(eq)
B7	6216(9)	1859.5(19)	4705.2(19)	45.5(13)
B01Q	7217(9)	2761.8(19)	4875.3(19)	43.4(13)
B01R	9262(9)	2497(2)	4656(2)	48.1(14)
B15	828(10)	3829(2)	3721(2)	52.9(16)
B8	8644(10)	1936(2)	4552(2)	51.2(15)
B14	2591(9)	3460(2)	3951(2)	48.9(14)
B2	9940(10)	2085(2)	5056(2)	50.4(15)
B18	3268(10)	3898(2)	3584(2)	52.5(15)
B10	3075(9)	3619(2)	4527(2)	48.9(14)
B6	6935(10)	2359(2)	4442(2)	51.6(15)
B13	4653(9)	3762(2)	4089(2)	50.0(14)
B16	1865(10)	4364(2)	3711(2)	56.9(16)
B12	2354(9)	4525(2)	4286(2)	50.1(15)
B17	4238(10)	4320(2)	3942(2)	52.9(15)
B11	296(10)	4220(2)	4154(2)	52.3(15)
C43	2199(13)	6430(3)	8528(3)	96(3)

Table S4 Anisotropic Displacement Parameters (Å^2×10^3) for cu_20231657_0m_4_sq. The Anisotropic displacement factor exponent takes the form: $-2\pi^2[h^2a^{*2}U_{11}+2hka^*b^*U_{12}+\ldots]$.

Atom	U_{11}	U_{22}	U_{33}	U_{23}	U_{13}	U_{12}
Cl1	44.2(7)	55.7(8)	52.1(7)	5.0(6)	-3.4(5)	-1.2(6)
Cl2	44.5(7)	50.3(7)	58.9(7)	8.3(6)	-2.3(6)	-4.3(6)
O1	51(2)	48(2)	42.8(18)	-3.5(15)	-1.8(16)	-2.4(16)
O2	48(2)	59(2)	43.4(18)	1.2(16)	4.7(16)	-7.4(17)
O5	54(2)	51(2)	48.0(19)	-2.5(16)	0.3(17)	7.4(17)
O4	57(2)	44.1(19)	46.5(19)	-1.8(15)	-1.2(16)	-0.2(17)
O3	67(3)	59(2)	56(2)	2.2(18)	-6(2)	5(2)
N1	42(2)	43(2)	45(2)	-4.7(18)	2.2(18)	-4.3(18)
N4	53(3)	46(2)	37(2)	4.7(18)	2.0(19)	4(2)
N2	39(2)	44(2)	43(2)	1.7(18)	-1.1(17)	1.3(18)
N5	35(2)	49(2)	58(2)	1(2)	-2.3(19)	-2.8(19)
N3	44(3)	56(3)	44(2)	-6(2)	-4(2)	-2(2)
O6	114(4)	60(3)	75(3)	-8(2)	-19(3)	-12(3)
N6	55(3)	61(3)	58(3)	-13(2)	-2(2)	-3(2)

Table S4 Anisotropic Displacement Parameters ($Å^2×10^3$) for cu_20231657_0m_4_sq. The Anisotropic displacement factor exponent takes the form: $-2\pi^2[h^2a^{*2}U_{11}+2hka^*b^*U_{12}+…]$.

Atom	U_{11}	U_{22}	U_{33}	U_{23}	U_{13}	U_{12}
C11	52(3)	45(3)	38(2)	-2(2)	2(2)	6(2)
C18	44(3)	53(3)	39(2)	-3(2)	-7(2)	-2(2)
C4	54(3)	30(2)	46(3)	-1(2)	-4(2)	-4(2)
C13	48(3)	35(3)	57(3)	0(2)	1(2)	1(2)
C24	40(3)	40(3)	46(3)	-1(2)	1(2)	2(2)
C15	54(3)	49(3)	46(3)	-2(2)	1(2)	6(3)
C12	51(3)	45(3)	42(3)	-1(2)	5(2)	7(2)
C34	55(3)	47(3)	53(3)	1(2)	11(3)	-1(3)
C33	50(3)	48(3)	45(3)	6(2)	11(2)	2(2)
C37	45(3)	48(3)	43(3)	4(2)	6(2)	-4(2)
C1	43(3)	40(3)	43(3)	1(2)	-3(2)	0(2)
C25	43(3)	42(3)	54(3)	9(2)	2(2)	-6(2)
C22	39(3)	42(3)	47(3)	3(2)	-7(2)	-5(2)
C3	53(3)	37(3)	45(3)	-3(2)	-7(2)	-3(2)
C9	42(3)	42(3)	47(3)	0(2)	-2(2)	-2(2)
C14	45(3)	39(3)	46(3)	-4(2)	0(2)	2(2)
C38	50(3)	50(3)	60(3)	-3(2)	-1(3)	11(3)
C36	44(3)	54(3)	48(3)	5(2)	3(2)	-2(2)
C20	52(3)	45(3)	50(3)	-5(2)	-4(3)	2(2)
C6	55(3)	41(3)	47(3)	-2(2)	-1(2)	-8(2)
C27	53(3)	36(3)	53(3)	2(2)	5(2)	7(2)
C17	51(3)	47(3)	45(3)	-6(2)	-2(2)	8(2)
C7	53(3)	50(3)	56(3)	-10(2)	3(3)	-6(3)
C32	63(4)	48(3)	46(3)	3(2)	2(3)	3(3)
C2	41(3)	39(3)	52(3)	0(2)	-3(2)	2(2)
C35	48(3)	52(3)	55(3)	10(2)	7(2)	2(3)
C30	50(3)	49(3)	54(3)	-3(2)	4(2)	-13(3)
C8	49(3)	55(3)	60(3)	-7(3)	-4(3)	3(3)
C19	54(3)	49(3)	47(3)	-5(2)	-4(2)	-7(3)
C23	53(3)	47(3)	43(3)	1(2)	0(2)	-7(2)
C39	45(3)	61(3)	53(3)	5(3)	2(2)	3(3)
C10	57(4)	54(3)	49(3)	-3(2)	-2(3)	-9(3)
C28	60(4)	50(3)	52(3)	5(2)	9(3)	1(3)
C26	57(4)	52(3)	53(3)	-8(2)	10(3)	-15(3)
C40	52(3)	56(3)	65(3)	-12(3)	-4(3)	-5(3)
C5	66(4)	52(3)	51(3)	-4(2)	-16(3)	7(3)

Table S4 Anisotropic Displacement Parameters ($Å^2×10^3$) for cu_20231657_0m_4_sq. The Anisotropic displacement factor exponent takes the form: $-2\pi^2[h^2a^{*2}U_{11}+2hka^*b^*U_{12}+...]$.

Atom	U_{11}	U_{22}	U_{33}	U_{23}	U_{13}	U_{12}
C29	54(3)	52(3)	58(3)	1(3)	5(3)	-3(3)
C21	57(4)	69(4)	57(3)	3(3)	7(3)	-11(3)
C16	59(4)	65(4)	54(3)	9(3)	8(3)	-11(3)
C41	57(4)	61(3)	58(3)	-3(3)	-14(3)	-2(3)
C42	97(6)	55(4)	63(4)	-16(3)	-9(4)	14(4)
B3	41(3)	41(3)	48(3)	-5(2)	5(2)	-9(3)
B4	44(3)	41(3)	48(3)	1(2)	-3(3)	-8(3)
B1	40(3)	40(3)	57(3)	-1(3)	-1(3)	-8(2)
B9	41(3)	58(4)	45(3)	2(3)	-7(3)	0(3)
B5	40(3)	48(3)	44(3)	-1(2)	-2(2)	-6(3)
B7	51(4)	43(3)	43(3)	-1(2)	6(3)	-9(3)
B01Q	49(3)	38(3)	44(3)	6(2)	-1(3)	-7(3)
B01R	47(4)	49(3)	50(3)	-3(3)	10(3)	-11(3)
B15	51(4)	63(4)	45(3)	1(3)	-7(3)	-11(3)
B8	55(4)	50(3)	49(3)	-5(3)	8(3)	-3(3)
B14	52(4)	48(3)	46(3)	-6(3)	3(3)	-8(3)
B2	50(4)	43(3)	58(4)	-2(3)	6(3)	-3(3)
B18	59(4)	57(4)	42(3)	1(3)	-1(3)	-9(3)
B10	52(4)	47(3)	47(3)	-1(3)	-2(3)	10(3)
B6	62(4)	48(3)	45(3)	-1(3)	-1(3)	-5(3)
B13	45(4)	56(4)	49(3)	2(3)	2(3)	9(3)
B16	61(4)	58(4)	51(4)	11(3)	0(3)	-4(3)
B12	51(4)	44(3)	55(3)	4(3)	0(3)	-8(3)
B17	50(4)	60(4)	48(3)	4(3)	4(3)	-6(3)
B11	44(3)	56(4)	56(4)	10(3)	-4(3)	-1(3)
C43	102(7)	88(5)	96(5)	-21(4)	-23(5)	36(5)

Table S5 Bond Lengths for cu_20231657_0m_4_sq.

Atom	Atom	Length/Å	Atom	Atom	Length/Å
O1	C11	1.364(6)	C27	C32	1.499(7)
O1	C10	1.459(7)	C27	C28	1.379(8)
O2	C15	1.358(6)	C7	C8	1.399(8)
O2	C16	1.431(7)	C2	B3	1.694(7)
O5	C37	1.367(6)	C2	B4	1.704(8)

Table S5 Bond Lengths for cu_20231657_0m_4_sq.

Atom	Atom	Length/Å	Atom	Atom	Length/Å
O5	C38	1.428(6)	C2	B1	1.744(8)
O4	C33	1.360(6)	C2	B2	1.686(8)
O4	C32	1.444(7)	C30	C29	1.370(8)
O3	C20	1.234(6)	C23	B15	1.701(8)
N1	C11	1.318(6)	C23	B14	1.709(8)
N1	C15	1.332(7)	C23	B10	1.686(8)
N4	C33	1.331(7)	C23	B11	1.704(8)
N4	C37	1.308(7)	C28	C29	1.392(8)
N2	C18	1.488(6)	C40	C41	1.492(9)
N2	C17	1.489(6)	C42	C43	1.500(10)
N5	C39	1.491(7)	B3	B4	1.765(9)
N5	C40	1.498(7)	B3	B7	1.769(9)
N3	C20	1.361(7)	B3	B8	1.766(8)
N3	C19	1.437(7)	B3	B2	1.765(8)
O6	C42	1.242(9)	B4	B5	1.786(8)
N6	C41	1.456(8)	B4	B7	1.788(8)
N6	C42	1.331(8)	B1	B01Q	1.759(8)
C11	C12	1.394(8)	B1	B01R	1.745(9)
C18	C19	1.521(8)	B1	B2	1.771(9)
C4	C3	1.424(7)	B9	B10	1.768(9)
C4	C6	1.398(7)	B9	B13	1.763(9)
C4	C5	1.520(8)	B9	B12	1.801(9)
C13	C12	1.393(7)	B9	B17	1.759(9)
C13	C14	1.386(7)	B5	B7	1.773(8)
C24	C25	1.413(7)	B5	B01Q	1.747(8)
C24	C22	1.543(7)	B5	B6	1.755(9)
C24	C30	1.399(7)	B7	B8	1.797(9)
C15	C14	1.399(8)	B7	B6	1.782(8)
C34	C33	1.387(8)	B01Q	B01R	1.785(9)
C34	C35	1.393(8)	B01Q	B6	1.769(8)
C37	C36	1.404(8)	B01R	B8	1.785(9)
C1	C3	1.506(7)	B01R	B2	1.772(9)
C1	C2	1.654(7)	B01R	B6	1.793(9)
C1	B4	1.752(7)	B15	B14	1.792(10)
C1	B1	1.745(8)	B15	B18	1.790(10)
C1	B5	1.740(7)	B15	B16	1.784(9)
C1	B01Q	1.718(7)	B15	B11	1.785(10)
C25	C27	1.406(7)	B8	B2	1.773(9)

Table S5 Bond Lengths for cu_20231657_0m_4_sq.

Atom	Atom	Length/Å	Atom	Atom	Length/Å
C25	C26	1.514(7)	B8	B6	1.787(10)
C22	C23	1.668(7)	B14	B18	1.782(9)
C22	B9	1.724(8)	B14	B10	1.778(8)
C22	B10	1.751(8)	B14	B13	1.759(9)
C22	B12	1.710(8)	B18	B13	1.799(9)
C22	B11	1.733(8)	B18	B16	1.772(10)
C3	C9	1.395(7)	B18	B17	1.783(9)
C9	C8	1.374(8)	B10	B13	1.773(9)
C14	C17	1.500(7)	B13	B17	1.772(9)
C36	C35	1.377(8)	B16	B12	1.779(9)
C36	C39	1.491(8)	B16	B17	1.795(10)
C20	C21	1.489(8)	B16	B11	1.780(9)
C6	C7	1.384(8)	B12	B17	1.799(10)
C6	C10	1.517(7)	B12	B11	1.758(9)

Table S6 Bond Angles for cu_20231657_0m_4_sq.

Atom	Atom	Atom	Angle/°	Atom	Atom	Atom	Angle/°
C11	O1	C10	116.7(4)	C1	B5	B4	59.6(3)
C15	O2	C16	117.0(4)	C1	B5	B7	106.9(4)
C37	O5	C38	117.1(4)	C1	B5	B01Q	59.0(3)
C33	O4	C32	116.3(4)	C1	B5	B6	106.7(4)
C11	N1	C15	115.8(5)	B7	B5	B4	60.3(3)
C37	N4	C33	116.6(5)	B01Q	B5	B4	108.6(4)
C18	N2	C17	113.8(4)	B01Q	B5	B7	109.4(4)
C39	N5	C40	114.4(4)	B01Q	B5	B6	60.7(3)
C20	N3	C19	123.9(5)	B6	B5	B4	108.9(5)
C42	N6	C41	123.8(6)	B6	B5	B7	60.7(3)
O1	C11	C12	115.8(4)	B3	B7	B4	59.5(3)
N1	C11	O1	118.6(5)	B3	B7	B5	106.8(4)
N1	C11	C12	125.5(5)	B3	B7	B8	59.3(3)
N2	C18	C19	111.2(4)	B3	B7	B6	106.9(4)
C3	C4	C5	125.4(5)	B4	B7	B8	107.3(4)
C6	C4	C3	117.6(5)	B5	B7	B4	60.2(3)
C6	C4	C5	116.9(4)	B5	B7	B8	106.8(4)
C14	C13	C12	120.4(5)	B5	B7	B6	59.2(3)
C25	C24	C22	123.7(4)	B6	B7	B4	107.6(4)

Table S6 Bond Angles for cu_20231657_0m_4_sq.

Atom	Atom	Atom	Angle/°	Atom	Atom	Atom	Angle/°
C30	C24	C25	120.0(5)	B6	B7	B8	59.9(4)
C30	C24	C22	116.4(4)	C1	B01Q	B1	60.2(3)
O2	C15	C14	115.5(5)	C1	B01Q	B5	60.3(3)
N1	C15	O2	119.1(5)	C1	B01Q	B01R	106.2(4)
N1	C15	C14	125.4(5)	C1	B01Q	B6	107.0(4)
C13	C12	C11	116.6(5)	B1	B01Q	B01R	59.0(3)
C33	C34	C35	117.5(5)	B1	B01Q	B6	108.4(4)
O4	C33	C34	116.4(5)	B5	B01Q	B1	109.7(4)
N4	C33	O4	119.7(5)	B5	B01Q	B01R	108.5(4)
N4	C33	C34	124.0(5)	B5	B01Q	B6	59.9(3)
O5	C37	C36	115.3(5)	B6	B01Q	B01R	60.6(4)
N4	C37	O5	119.0(5)	B1	B01R	B01Q	59.7(3)
N4	C37	C36	125.8(5)	B1	B01R	B8	108.9(4)
C3	C1	C2	121.2(4)	B1	B01R	B2	60.5(3)
C3	C1	B4	118.9(4)	B1	B01R	B6	108.0(4)
C3	C1	B1	118.3(4)	B01Q	B01R	B6	59.3(3)
C3	C1	B5	122.2(4)	B8	B01R	B01Q	107.2(4)
C3	C1	B01Q	121.3(4)	B8	B01R	B6	59.9(4)
C2	C1	B4	59.9(3)	B2	B01R	B01Q	106.8(4)
C2	C1	B1	61.7(3)	B2	B01R	B8	59.8(4)
C2	C1	B5	107.6(4)	B2	B01R	B6	107.1(4)
C2	C1	B01Q	108.7(4)	C23	B15	B14	58.5(3)
B1	C1	B4	112.6(4)	C23	B15	B18	103.8(4)
B5	C1	B4	61.5(3)	C23	B15	B16	103.6(4)
B5	C1	B1	110.7(4)	C23	B15	B11	58.5(3)
B01Q	C1	B4	111.5(4)	B18	B15	B14	59.7(4)
B01Q	C1	B1	61.0(3)	B16	B15	B14	107.1(5)
B01Q	C1	B5	60.7(3)	B16	B15	B18	59.5(4)
C24	C25	C26	124.7(5)	B16	B15	B11	59.8(4)
C27	C25	C24	117.7(5)	B11	B15	B14	107.8(4)
C27	C25	C26	117.6(5)	B11	B15	B18	107.5(4)
C24	C22	C23	116.5(4)	B3	B8	B7	59.5(3)
C24	C22	B9	126.5(4)	B3	B8	B01R	107.6(4)
C24	C22	B10	117.6(4)	B3	B8	B2	59.8(3)
C24	C22	B12	124.5(4)	B3	B8	B6	106.9(5)
C24	C22	B11	115.7(4)	B01R	B8	B7	108.2(4)
C23	C22	B9	106.8(4)	B01R	B8	B6	60.3(4)
C23	C22	B10	59.0(3)	B2	B8	B7	107.5(4)

Table S6 Bond Angles for cu_20231657_0m_4_sq.

Atom	Atom	Atom	Angle/°	Atom	Atom	Atom	Angle/°
C23	C22	B12	107.9(4)	B2	B8	B01R	59.7(4)
C23	C22	B11	60.1(3)	B2	B8	B6	107.4(4)
B9	C22	B10	61.2(4)	B6	B8	B7	59.6(4)
B9	C22	B11	112.4(4)	C23	B14	B15	58.1(3)
B12	C22	B9	63.3(4)	C23	B14	B18	103.8(4)
B12	C22	B10	112.9(4)	C23	B14	B10	57.8(3)
B12	C22	B11	61.4(3)	C23	B14	B13	103.8(4)
B11	C22	B10	111.3(4)	B18	B14	B15	60.1(4)
C4	C3	C1	126.1(5)	B10	B14	B15	107.6(4)
C9	C3	C4	119.2(5)	B10	B14	B18	108.7(4)
C9	C3	C1	114.7(4)	B13	B14	B15	108.8(5)
C8	C9	C3	122.6(5)	B13	B14	B18	61.1(4)
C13	C14	C15	116.3(5)	B13	B14	B10	60.2(4)
C13	C14	C17	123.6(5)	C2	B2	B3	58.7(3)
C15	C14	C17	120.1(5)	C2	B2	B1	60.6(3)
C37	C36	C39	119.9(5)	C2	B2	B01R	105.5(4)
C35	C36	C37	115.9(5)	C2	B2	B8	105.2(5)
C35	C36	C39	124.1(5)	B3	B2	B1	109.3(5)
O3	C20	N3	120.7(5)	B3	B2	B01R	108.3(5)
O3	C20	C21	123.4(5)	B3	B2	B8	59.9(4)
N3	C20	C21	115.9(5)	B1	B2	B01R	59.0(3)
C4	C6	C10	122.6(5)	B1	B2	B8	108.2(5)
C7	C6	C4	121.6(5)	B01R	B2	B8	60.5(4)
C7	C6	C10	115.7(5)	B15	B18	B13	107.2(4)
C25	C27	C32	121.4(5)	B14	B18	B15	60.2(4)
C28	C27	C25	120.9(5)	B14	B18	B13	58.8(4)
C28	C27	C32	117.7(5)	B14	B18	B17	106.7(4)
N2	C17	C14	111.1(4)	B16	B18	B15	60.1(4)
C6	C7	C8	120.8(5)	B16	B18	B14	108.1(5)
O4	C32	C27	106.9(4)	B16	B18	B13	107.8(4)
C1	C2	B3	112.9(4)	B16	B18	B17	60.7(4)
C1	C2	B4	62.9(3)	B17	B18	B15	108.1(5)
C1	C2	B1	61.7(3)	B17	B18	B13	59.3(4)
C1	C2	B2	112.6(4)	C22	B10	B9	58.6(3)
B3	C2	B4	62.6(3)	C22	B10	B14	106.6(4)
B3	C2	B1	114.0(4)	C22	B10	B13	105.6(4)
B4	C2	B1	115.1(4)	C23	B10	C22	58.0(3)
B2	C2	B3	62.9(3)	C23	B10	B9	104.0(4)

Table S6 Bond Angles for cu_20231657_0m_4_sq.

Atom	Atom	Atom	Angle/°	Atom	Atom	Atom	Angle/°
B2	C2	B4	115.3(4)	C23	B10	B14	59.1(3)
B2	C2	B1	62.1(3)	C23	B10	B13	104.1(4)
C36	C35	C34	120.1(5)	B9	B10	B14	107.3(4)
C29	C30	C24	121.2(5)	B9	B10	B13	59.7(4)
C9	C8	C7	118.1(5)	B13	B10	B14	59.4(4)
N3	C19	C18	112.1(4)	B5	B6	B7	60.1(3)
C22	C23	B15	113.7(4)	B5	B6	B01Q	59.4(3)
C22	C23	B14	113.8(4)	B5	B6	B01R	107.8(4)
C22	C23	B10	62.9(3)	B5	B6	B8	108.1(4)
C22	C23	B11	61.9(3)	B7	B6	B01R	108.5(4)
B15	C23	B14	63.4(4)	B7	B6	B8	60.5(4)
B15	C23	B11	63.2(4)	B01Q	B6	B7	107.9(4)
B10	C23	B15	116.5(5)	B01Q	B6	B01R	60.1(4)
B10	C23	B14	63.2(4)	B01Q	B6	B8	107.8(4)
B10	C23	B11	116.1(4)	B8	B6	B01R	59.8(4)
B11	C23	B14	115.8(4)	B9	B13	B18	107.7(4)
N5	C39	C36	111.7(5)	B9	B13	B10	60.0(4)
O1	C10	C6	105.8(4)	B9	B13	B17	59.7(4)
C27	C28	C29	120.9(5)	B14	B13	B9	108.4(5)
C41	C40	N5	110.4(5)	B14	B13	B18	60.1(4)
C30	C29	C28	119.1(6)	B14	B13	B10	60.4(4)
N6	C41	C40	111.5(5)	B14	B13	B17	108.2(5)
O6	C42	N6	120.8(7)	B10	B13	B18	108.2(5)
O6	C42	C43	122.2(7)	B17	B13	B18	59.9(4)
N6	C42	C43	117.0(7)	B17	B13	B10	107.9(4)
C2	B3	B4	59.0(3)	B15	B16	B17	107.8(5)
C2	B3	B7	105.7(4)	B18	B16	B15	60.4(4)
C2	B3	B8	105.2(4)	B18	B16	B12	108.8(5)
C2	B3	B2	58.3(3)	B18	B16	B17	60.0(4)
B4	B3	B7	60.8(3)	B18	B16	B11	108.5(5)
B4	B3	B8	109.7(4)	B12	B16	B15	107.8(4)
B8	B3	B7	61.1(4)	B12	B16	B17	60.4(4)
B2	B3	B4	108.4(4)	B12	B16	B11	59.2(4)
B2	B3	B7	109.2(4)	B11	B16	B15	60.1(4)
B2	B3	B8	60.3(4)	B11	B16	B17	107.4(4)
C1	B4	B3	105.1(4)	C22	B12	B9	58.7(3)
C1	B4	B5	58.9(3)	C22	B12	B16	106.8(4)
C1	B4	B7	105.8(4)	C22	B12	B17	105.2(4)

Table S6 Bond Angles for cu_20231657_0m_4_sq.

Atom	Atom	Atom	Angle/°	Atom	Atom	Atom	Angle/°
C2	B4	C1	57.2(3)	C22	B12	B11	60.0(3)
C2	B4	B3	58.4(3)	B16	B12	B9	106.9(5)
C2	B4	B5	103.5(4)	B16	B12	B17	60.2(4)
C2	B4	B7	104.5(4)	B17	B12	B9	58.5(4)
B3	B4	B5	106.4(4)	B11	B12	B9	107.7(4)
B3	B4	B7	59.7(3)	B11	B12	B16	60.4(4)
B5	B4	B7	59.5(3)	B11	B12	B17	108.2(5)
C1	B1	B01Q	58.7(3)	B9	B17	B18	108.6(5)
C1	B1	B01R	106.8(4)	B9	B17	B13	59.9(4)
C1	B1	B2	104.5(4)	B9	B17	B16	108.1(5)
C2	B1	C1	56.6(3)	B9	B17	B12	60.8(4)
C2	B1	B01Q	103.0(4)	B18	B17	B16	59.4(4)
C2	B1	B01R	104.2(4)	B18	B17	B12	107.4(5)
C2	B1	B2	57.3(3)	B13	B17	B18	60.8(4)
B01Q	B1	B2	108.1(4)	B13	B17	B16	108.0(5)
B01R	B1	B01Q	61.2(4)	B13	B17	B12	108.4(4)
B01R	B1	B2	60.5(4)	B16	B17	B12	59.3(4)
C22	B9	B10	60.2(3)	C22	B11	B15	106.6(5)
C22	B9	B13	107.2(4)	C22	B11	B16	105.8(5)
C22	B9	B12	58.0(3)	C22	B11	B12	58.7(3)
C22	B9	B17	106.4(4)	C23	B11	C22	58.0(3)
B10	B9	B12	107.9(4)	C23	B11	B15	58.3(3)
B13	B9	B10	60.3(4)	C23	B11	B16	103.7(5)
B13	B9	B12	108.8(4)	C23	B11	B12	104.2(4)
B17	B9	B10	108.7(4)	B16	B11	B15	60.1(4)
B17	B9	B13	60.4(4)	B12	B11	B15	108.7(5)
B17	B9	B12	60.7(4)	B12	B11	B16	60.4(4)

Table S7 Torsion Angles for cu_20231657_0m_4_sq.

A	B	C	D	Angle/°	A	B	C	D	Angle/°
O1	C11	C12	C13	179.7(4)	B5	B4	B7	B3	136.4(4)
O2	C15	C14	C13	180.0(4)	B5	B4	B7	B8	99.8(4)
O2	C15	C14	C17	-1.3(7)	B5	B4	B7	B6	36.7(4)
O5	C37	C36	C35	179.6(4)	B5	B7	B8	B3	99.9(4)
O5	C37	C36	C39	1.0(7)	B5	B7	B8	B01R	-0.3(6)
N1	C11	C12	C13	-1.5(8)	B5	B7	B8	B2	62.8(5)

Table S7 Torsion Angles for cu_20231657_0m_4_sq.

A	B	C	D	Angle/°	A	B	C	D	Angle/°
N1	C15	C14	C13	1.3(8)	B5	B7	B8	B6	-37.4(4)
N1	C15	C14	C17	179.9(5)	B5	B7	B6	B01Q	36.6(4)
N4	C37	C36	C35	-1.2(8)	B5	B7	B6	B01R	100.3(5)
N4	C37	C36	C39	-179.8(5)	B5	B7	B6	B8	137.3(4)
N2	C18	C19	N3	64.9(6)	B5	B01Q	B01R	B1	-102.3(4)
N5	C40	C41	N6	-65.0(7)	B5	B01Q	B01R	B8	-0.1(5)
C11	O1	C10	C6	-176.6(4)	B5	B01Q	B01R	B2	-63.0(5)
C11	N1	C15	O2	-179.7(5)	B5	B01Q	B01R	B6	37.3(4)
C11	N1	C15	C14	-1.1(8)	B5	B01Q	B6	B7	-37.0(4)
C18	N2	C17	C14	175.7(4)	B5	B01Q	B6	B01R	-138.4(4)
C4	C3	C9	C8	0.0(8)	B5	B01Q	B6	B8	-100.9(5)
C4	C6	C7	C8	0.7(9)	B7	B3	B4	C1	-99.7(4)
C4	C6	C10	O1	71.3(6)	B7	B3	B4	C2	-134.2(4)
C13	C14	C17	N2	98.8(6)	B7	B3	B4	B5	-38.3(4)
C24	C25	C27	C32	179.2(5)	B7	B3	B8	B01R	101.1(5)
C24	C25	C27	C28	-1.9(8)	B7	B3	B8	B2	138.3(5)
C24	C22	C23	B15	143.3(5)	B7	B3	B8	B6	37.7(4)
C24	C22	C23	B14	-146.6(4)	B7	B3	B2	C2	97.0(4)
C24	C22	C23	B10	-107.8(5)	B7	B3	B2	B1	62.3(5)
C24	C22	C23	B11	105.8(5)	B7	B3	B2	B01R	-0.4(6)
C24	C22	B9	B10	104.6(5)	B7	B3	B2	B8	-38.1(4)
C24	C22	B9	B13	143.6(5)	B7	B4	B5	C1	-136.4(4)
C24	C22	B9	B12	-114.6(5)	B7	B4	B5	B01Q	-102.2(5)
C24	C22	B9	B17	-152.9(5)	B7	B4	B5	B6	-37.7(4)
C24	C22	B10	C23	105.9(5)	B7	B5	B01Q	C1	-98.6(4)
C24	C22	B10	B9	-118.5(5)	B7	B5	B01Q	B1	-62.8(5)
C24	C22	B10	B14	141.0(4)	B7	B5	B01Q	B01R	0.0(5)
C24	C22	B10	B13	-157.0(4)	B7	B5	B01Q	B6	37.6(4)
C24	C22	B12	B9	117.5(5)	B7	B5	B6	B01Q	-138.7(5)
C24	C22	B12	B16	-142.6(5)	B7	B5	B6	B01R	-101.5(5)
C24	C22	B12	B17	154.5(5)	B7	B5	B6	B8	-38.3(4)
C24	C22	B12	B11	-103.0(5)	B7	B8	B2	C2	-1.8(6)
C24	C22	B11	C23	-107.1(5)	B7	B8	B2	B3	37.0(4)
C24	C22	B11	B15	-140.9(4)	B7	B8	B2	B1	-65.3(5)
C24	C22	B11	B16	156.3(4)	B7	B8	B2	B01R	-101.2(5)
C24	C22	B11	B12	117.0(5)	B7	B8	B6	B5	38.2(4)
C24	C30	C29	C28	2.1(9)	B7	B8	B6	B01Q	101.0(5)
C15	N1	C11	O1	180.0(4)	B7	B8	B6	B01R	138.6(4)

Table S7 Torsion Angles for cu_20231657_0m_4_sq.

A	B	C	D	Angle/°	A	B	C	D	Angle/°
C15	N1	C11	C12	1.2(8)	B01Q	C1	C3	C4	-119.3(6)
C15	C14	C17	N2	-79.8(6)	B01Q	C1	C3	C9	58.5(7)
C12	C13	C14	C15	-1.5(7)	B01Q	C1	C2	B3	-65.7(5)
C12	C13	C14	C17	179.8(5)	B01Q	C1	C2	B4	-104.6(4)
C33	O4	C32	C27	171.2(4)	B01Q	C1	C2	B1	40.2(4)
C33	N4	C37	O5	177.8(4)	B01Q	C1	C2	B2	3.3(5)
C33	N4	C37	C36	-1.4(8)	B01Q	C1	B4	C2	99.8(5)
C33	C34	C35	C36	-1.4(8)	B01Q	C1	B4	B3	64.7(5)
C37	N4	C33	O4	-177.3(4)	B01Q	C1	B4	B5	-35.6(4)
C37	N4	C33	C34	2.7(8)	B01Q	C1	B4	B7	2.5(6)
C37	C36	C35	C34	2.5(8)	B01Q	C1	B1	C2	-135.6(4)
C37	C36	C39	N5	72.5(6)	B01Q	C1	B1	B01R	-39.5(4)
C1	C3	C9	C8	-178.0(5)	B01Q	C1	B1	B2	-102.6(4)
C1	C2	B3	B4	-39.0(4)	B01Q	C1	B5	B4	141.6(4)
C1	C2	B3	B7	1.5(5)	B01Q	C1	B5	B7	102.8(4)
C1	C2	B3	B8	65.2(5)	B01Q	C1	B5	B6	39.1(4)
C1	C2	B3	B2	104.6(5)	B01Q	B1	B01R	B8	-99.3(5)
C1	C2	B4	B3	139.4(4)	B01Q	B1	B01R	B2	-135.7(4)
C1	C2	B4	B5	38.2(4)	B01Q	B1	B01R	B6	-35.8(4)
C1	C2	B4	B7	99.7(4)	B01Q	B1	B2	C2	-94.1(4)
C1	C2	B1	B01Q	-37.8(4)	B01Q	B1	B2	B3	-60.1(5)
C1	C2	B1	B01R	-101.0(4)	B01Q	B1	B2	B01R	40.1(4)
C1	C2	B1	B2	-141.1(4)	B01Q	B1	B2	B8	3.5(6)
C1	C2	B2	B3	-105.2(5)	B01Q	B5	B7	B3	62.5(5)
C1	C2	B2	B1	36.8(4)	B01Q	B5	B7	B4	100.9(4)
C1	C2	B2	B01R	-2.9(6)	B01Q	B5	B7	B8	0.2(5)
C1	C2	B2	B8	-65.8(5)	B01Q	B5	B7	B6	-37.6(4)
C1	B4	B5	B7	136.4(4)	B01Q	B5	B6	B7	138.7(5)
C1	B4	B5	B01Q	34.2(4)	B01Q	B5	B6	B01R	37.2(4)
C1	B4	B5	B6	98.7(4)	B01Q	B5	B6	B8	100.4(5)
C1	B4	B7	B3	98.5(4)	B01Q	B01R	B8	B3	-62.6(5)
C1	B4	B7	B5	-37.9(4)	B01Q	B01R	B8	B7	0.3(6)
C1	B4	B7	B8	61.9(5)	B01Q	B01R	B8	B2	-99.9(5)
C1	B4	B7	B6	-1.2(6)	B01Q	B01R	B8	B6	37.1(4)
C1	B1	B01Q	B5	-35.7(4)	B01Q	B01R	B2	C2	1.4(5)
C1	B1	B01Q	B01R	-136.0(4)	B01Q	B01R	B2	B3	63.0(5)
C1	B1	B01Q	B6	-99.5(4)	B01Q	B01R	B2	B1	-39.0(4)
C1	B1	B01R	B01Q	38.3(4)	B01Q	B01R	B2	B8	100.4(5)

Table S7 Torsion Angles for cu_20231657_0m_4_sq.

A	B	C	D	Angle/°	A	B	C	D	Angle/°
C1	B1	B01R	B8	-61.0(5)	B01Q	B01R	B6	B5	-36.9(4)
C1	B1	B01R	B2	-97.4(4)	B01Q	B01R	B6	B7	-100.5(5)
C1	B1	B01R	B6	2.5(5)	B01Q	B01R	B6	B8	-137.8(4)
C1	B1	B2	C2	-32.7(4)	B01R	B1	B01Q	C1	136.0(4)
C1	B1	B2	B3	1.2(5)	B01R	B1	B01Q	B5	100.3(5)
C1	B1	B2	B01R	101.4(4)	B01R	B1	B01Q	B6	36.5(4)
C1	B1	B2	B8	64.8(5)	B01R	B1	B2	C2	-134.1(4)
C1	B5	B7	B3	0.1(5)	B01R	B1	B2	B3	-100.2(5)
C1	B5	B7	B4	38.4(4)	B01R	B1	B2	B8	-36.6(4)
C1	B5	B7	B8	-62.2(5)	B01R	B01Q	B6	B5	138.4(4)
C1	B5	B7	B6	-100.0(4)	B01R	B01Q	B6	B7	101.4(5)
C1	B5	B01Q	B1	35.7(4)	B01R	B01Q	B6	B8	37.5(4)
C1	B5	B01Q	B01R	98.5(4)	B01R	B8	B2	C2	99.4(5)
C1	B5	B01Q	B6	136.1(4)	B01R	B8	B2	B3	138.2(5)
C1	B5	B6	B7	100.4(4)	B01R	B8	B2	B1	35.9(4)
C1	B5	B6	B01Q	-38.4(4)	B01R	B8	B6	B5	-100.5(5)
C1	B5	B6	B01R	-1.2(6)	B01R	B8	B6	B7	-138.6(4)
C1	B5	B6	B8	62.1(5)	B01R	B8	B6	B01Q	-37.7(4)
C1	B01Q	B01R	B1	-38.9(4)	B15	C23	B14	B18	40.8(4)
C1	B01Q	B01R	B8	63.3(5)	B15	C23	B14	B10	144.4(5)
C1	B01Q	B01R	B2	0.5(5)	B15	C23	B14	B13	103.8(5)
C1	B01Q	B01R	B6	100.7(4)	B15	C23	B10	C22	104.4(5)
C1	B01Q	B6	B5	39.0(4)	B15	C23	B10	B9	66.4(6)
C1	B01Q	B6	B7	2.1(6)	B15	C23	B10	B14	-35.6(5)
C1	B01Q	B6	B01R	-99.4(5)	B15	C23	B10	B13	4.7(6)
C1	B01Q	B6	B8	-61.8(5)	B15	C23	B11	C22	-141.3(5)
C25	C24	C22	C23	165.1(5)	B15	C23	B11	B16	-41.1(4)
C25	C24	C22	B9	24.8(8)	B15	C23	B11	B12	-103.5(5)
C25	C24	C22	B10	97.9(6)	B15	B14	B18	B13	138.0(5)
C25	C24	C22	B12	-55.4(7)	B15	B14	B18	B16	37.7(4)
C25	C24	C22	B11	-127.1(5)	B15	B14	B18	B17	101.6(5)
C25	C24	C30	C29	-5.0(8)	B15	B14	B10	C22	-3.4(6)
C25	C27	C32	O4	-71.2(6)	B15	B14	B10	C23	31.2(4)
C25	C27	C28	C29	-0.8(8)	B15	B14	B10	B9	-65.0(5)
C22	C24	C25	C27	-175.4(5)	B15	B14	B10	B13	-102.1(5)
C22	C24	C25	C26	5.5(8)	B15	B14	B13	B9	62.4(5)
C22	C24	C30	C29	175.2(5)	B15	B14	B13	B18	-37.8(4)
C22	C23	B15	B14	105.9(5)	B15	B14	B13	B10	99.9(5)

Table S7 Torsion Angles for cu_20231657_0m_4_sq.

A	B	C	D	Angle/°	A	B	C	D	Angle/°
C22	C23	B15	B18	65.3(6)	B15	B14	B13	B17	-0.8(6)
C22	C23	B15	B16	3.9(6)	B15	B18	B13	B9	-64.1(6)
C22	C23	B15	B11	-37.0(4)	B15	B18	B13	B14	37.4(4)
C22	C23	B14	B15	-105.7(5)	B15	B18	B13	B10	-0.6(6)
C22	C23	B14	B18	-64.9(5)	B15	B18	B13	B17	-101.2(5)
C22	C23	B14	B10	38.7(4)	B15	B18	B16	B12	100.2(5)
C22	C23	B14	B13	-1.9(6)	B15	B18	B16	B17	137.2(5)
C22	C23	B10	B9	-38.0(4)	B15	B18	B16	B11	37.4(4)
C22	C23	B10	B14	-140.0(4)	B15	B18	B17	B9	62.2(6)
C22	C23	B10	B13	-99.8(4)	B15	B18	B17	B13	99.6(5)
C22	C23	B11	B15	141.3(5)	B15	B18	B17	B16	-38.3(4)
C22	C23	B11	B16	100.2(5)	B15	B18	B17	B12	-2.1(6)
C22	C23	B11	B12	37.8(4)	B15	B16	B12	C22	2.6(6)
C22	B9	B10	C23	37.7(4)	B15	B16	B12	B9	64.3(6)
C22	B9	B10	B14	99.2(4)	B15	B16	B12	B17	100.8(5)
C22	B9	B10	B13	136.1(4)	B15	B16	B12	B11	-36.8(5)
C22	B9	B13	B14	-1.4(6)	B15	B16	B17	B9	-63.0(6)
C22	B9	B13	B18	62.2(5)	B15	B16	B17	B18	38.4(4)
C22	B9	B13	B10	-39.0(4)	B15	B16	B17	B13	0.4(6)
C22	B9	B13	B17	99.4(5)	B15	B16	B17	B12	-100.8(5)
C22	B9	B12	B16	-99.7(5)	B15	B16	B11	C22	100.3(5)
C22	B9	B12	B17	-137.0(4)	B15	B16	B11	C23	40.2(4)
C22	B9	B12	B11	-36.1(4)	B15	B16	B11	B12	138.9(5)
C22	B9	B17	B18	-63.0(6)	B8	B3	B4	C1	-61.8(5)
C22	B9	B17	B13	-100.9(5)	B8	B3	B4	C2	-96.3(4)
C22	B9	B17	B16	-0.1(6)	B8	B3	B4	B5	-0.3(5)
C22	B9	B17	B12	37.1(4)	B8	B3	B4	B7	37.9(4)
C22	B10	B13	B9	37.9(4)	B8	B3	B7	B4	-138.6(4)
C22	B10	B13	B14	-100.3(5)	B8	B3	B7	B5	-99.9(4)
C22	B10	B13	B18	-62.4(5)	B8	B3	B7	B6	-37.8(4)
C22	B10	B13	B17	0.9(6)	B8	B3	B2	C2	135.0(5)
C22	B12	B17	B9	-37.1(4)	B8	B3	B2	B1	100.4(5)
C22	B12	B17	B18	64.8(5)	B8	B3	B2	B01R	37.7(4)
C22	B12	B17	B13	0.5(6)	B8	B7	B6	B5	-137.3(4)
C22	B12	B17	B16	101.0(5)	B8	B7	B6	B01Q	-100.7(5)
C22	B12	B11	C23	-37.5(4)	B8	B7	B6	B01R	-37.0(4)
C22	B12	B11	B15	-98.4(5)	B8	B01R	B2	C2	-99.0(5)
C22	B12	B11	B16	-135.4(5)	B8	B01R	B2	B3	-37.4(4)

Table S7 Torsion Angles for cu_20231657_0m_4_sq.

A	B	C	D	Angle/°	A	B	C	D	Angle/°
C3	C4	C6	C7	-0.8(8)	B8	B01R	B2	B1	-139.4(5)
C3	C4	C6	C10	180.0(5)	B8	B01R	B6	B5	101.0(5)
C3	C1	C2	B3	146.4(5)	B8	B01R	B6	B7	37.3(4)
C3	C1	C2	B4	107.5(5)	B8	B01R	B6	B01Q	137.8(4)
C3	C1	C2	B1	-107.7(5)	B14	C23	B15	B18	-40.6(4)
C3	C1	C2	B2	-144.6(5)	B14	C23	B15	B16	-101.9(5)
C3	C1	B4	C2	-111.4(5)	B14	C23	B15	B11	-142.9(5)
C3	C1	B4	B3	-146.5(4)	B14	C23	B10	C22	140.0(4)
C3	C1	B4	B5	113.2(5)	B14	C23	B10	B9	102.0(5)
C3	C1	B4	B7	151.3(4)	B14	C23	B10	B13	40.3(4)
C3	C1	B1	C2	112.3(5)	B14	C23	B11	C22	-104.5(5)
C3	C1	B1	B01Q	-112.1(5)	B14	C23	B11	B15	36.8(4)
C3	C1	B1	B01R	-151.6(4)	B14	C23	B11	B16	-4.2(6)
C3	C1	B1	B2	145.3(4)	B14	C23	B11	B12	-66.6(6)
C3	C1	B5	B4	-107.9(5)	B14	B15	B18	B13	-36.8(4)
C3	C1	B5	B7	-146.7(4)	B14	B15	B18	B16	-137.9(5)
C3	C1	B5	B01Q	110.5(5)	B14	B15	B18	B17	-99.3(5)
C3	C1	B5	B6	149.6(5)	B14	B15	B16	B18	37.3(4)
C3	C1	B01Q	B1	107.3(5)	B14	B15	B16	B12	-64.7(6)
C3	C1	B01Q	B5	-112.0(5)	B14	B15	B16	B17	-0.8(6)
C3	C1	B01Q	B01R	145.7(5)	B14	B15	B16	B11	-101.1(5)
C3	C1	B01Q	B6	-150.8(5)	B14	B15	B11	C22	0.9(6)
C3	C9	C8	C7	-0.1(8)	B14	B15	B11	C23	-32.7(4)
C14	C13	C12	C11	1.7(7)	B14	B15	B11	B16	99.8(5)
C38	O5	C37	N4	0.7(7)	B14	B15	B11	B12	62.7(5)
C38	O5	C37	C36	180.0(5)	B14	B18	B13	B9	-101.5(5)
C20	N3	C19	C18	92.5(6)	B14	B18	B13	B10	-38.1(4)
C6	C4	C3	C1	178.2(5)	B14	B18	B13	B17	-138.6(5)
C6	C4	C3	C9	0.4(7)	B14	B18	B16	B15	-37.8(4)
C6	C7	C8	C9	-0.2(8)	B14	B18	B16	B12	62.5(6)
C27	C28	C29	C30	0.8(9)	B14	B18	B16	B17	99.4(5)
C17	N2	C18	C19	-175.3(4)	B14	B18	B16	B11	-0.4(6)
C7	C6	C10	O1	-108.0(6)	B14	B18	B17	B9	-1.3(6)
C32	O4	C33	N4	-0.9(7)	B14	B18	B17	B13	36.2(4)
C32	O4	C33	C34	179.0(5)	B14	B18	B17	B16	-101.7(5)
C32	C27	C28	C29	178.1(5)	B14	B18	B17	B12	-65.6(6)
C2	C1	C3	C4	24.6(8)	B14	B10	B13	B9	138.2(5)
C2	C1	C3	C9	-157.6(5)	B14	B10	B13	B18	37.9(4)

Table S7 Torsion Angles for cu_20231657_0m_4_sq.

A	B	C	D	Angle/°	A	B	C	D	Angle/°
C2	C1	B4	B3	-35.1(4)	B14	B10	B13	B17	101.2(5)
C2	C1	B4	B5	-135.4(4)	B14	B13	B17	B9	101.1(5)
C2	C1	B4	B7	-97.3(5)	B14	B13	B17	B18	-37.1(4)
C2	C1	B1	B01Q	135.6(4)	B14	B13	B17	B16	0.2(6)
C2	C1	B1	B01R	96.1(4)	B14	B13	B17	B12	63.0(5)
C2	C1	B1	B2	33.0(4)	B2	C2	B3	B4	-143.6(4)
C2	C1	B5	B4	39.6(4)	B2	C2	B3	B7	-103.2(4)
C2	C1	B5	B7	0.8(5)	B2	C2	B3	B8	-39.5(4)
C2	C1	B5	B01Q	-102.0(4)	B2	C2	B4	C1	-103.6(5)
C2	C1	B5	B6	-62.9(5)	B2	C2	B4	B3	35.7(4)
C2	C1	B01Q	B1	-40.5(4)	B2	C2	B4	B5	-65.5(5)
C2	C1	B01Q	B5	100.2(4)	B2	C2	B4	B7	-4.0(6)
C2	C1	B01Q	B01R	-2.2(5)	B2	C2	B1	C1	141.1(4)
C2	C1	B01Q	B6	61.3(5)	B2	C2	B1	B01Q	103.3(5)
C2	B3	B4	C1	34.5(3)	B2	C2	B1	B01R	40.1(4)
C2	B3	B4	B5	96.0(4)	B2	B3	B4	C1	2.4(5)
C2	B3	B4	B7	134.2(4)	B2	B3	B4	C2	-32.1(4)
C2	B3	B7	B4	-39.6(4)	B2	B3	B4	B5	63.8(5)
C2	B3	B7	B5	-0.9(5)	B2	B3	B4	B7	102.1(5)
C2	B3	B7	B8	99.0(4)	B2	B3	B7	B4	-100.9(4)
C2	B3	B7	B6	61.2(5)	B2	B3	B7	B5	-62.2(5)
C2	B3	B8	B7	-99.7(4)	B2	B3	B7	B8	37.7(4)
C2	B3	B8	B01R	1.3(6)	B2	B3	B7	B6	0.0(5)
C2	B3	B8	B2	38.5(4)	B2	B3	B8	B7	-138.3(5)
C2	B3	B8	B6	-62.1(5)	B2	B3	B8	B01R	-37.2(4)
C2	B3	B2	B1	-34.7(4)	B2	B3	B8	B6	-100.6(5)
C2	B3	B2	B01R	-97.4(5)	B2	B1	B01Q	C1	96.3(4)
C2	B3	B2	B8	-135.0(5)	B2	B1	B01Q	B5	60.5(5)
C2	B4	B5	C1	-37.3(4)	B2	B1	B01Q	B01R	-39.7(4)
C2	B4	B5	B7	99.0(4)	B2	B1	B01Q	B6	-3.2(6)
C2	B4	B5	B01Q	-3.1(5)	B2	B1	B01R	B01Q	135.7(4)
C2	B4	B5	B6	61.3(5)	B2	B1	B01R	B8	36.4(4)
C2	B4	B7	B3	39.1(4)	B2	B1	B01R	B6	99.9(5)
C2	B4	B7	B5	-97.3(4)	B2	B01R	B8	B3	37.2(4)
C2	B4	B7	B8	2.5(5)	B2	B01R	B8	B7	100.1(5)
C2	B4	B7	B6	-60.6(5)	B2	B01R	B8	B6	137.0(5)
C2	B1	B01Q	C1	36.8(4)	B2	B01R	B6	B5	62.9(5)
C2	B1	B01Q	B5	1.1(5)	B2	B01R	B6	B7	-0.8(6)

Table S7 Torsion Angles for cu_20231657_0m_4_sq.

A	B	C	D	Angle/°	A	B	C	D	Angle/°
C2	B1	B01Q	B01R	-99.2(4)	B2	B01R	B6	B01Q	99.8(4)
C2	B1	B01Q	B6	-62.7(5)	B2	B01R	B6	B8	-38.1(4)
C2	B1	B01R	B01Q	97.2(4)	B2	B8	B6	B5	-62.3(6)
C2	B1	B01R	B8	-2.1(6)	B2	B8	B6	B7	-100.5(5)
C2	B1	B01R	B2	-38.5(4)	B2	B8	B6	B01Q	0.4(6)
C2	B1	B01R	B6	61.4(5)	B2	B8	B6	B01R	38.1(4)
C2	B1	B2	B3	34.0(4)	B18	B15	B14	C23	133.0(4)
C2	B1	B2	B01R	134.1(5)	B18	B15	B14	B10	101.9(5)
C2	B1	B2	B8	97.6(5)	B18	B15	B14	B13	38.2(4)
C35	C34	C33	O4	178.6(4)	B18	B15	B16	B12	-102.0(5)
C35	C34	C33	N4	-1.4(8)	B18	B15	B16	B17	-38.1(4)
C35	C36	C39	N5	-106.0(6)	B18	B15	B16	B11	-138.4(5)
C30	C24	C25	C27	4.8(8)	B18	B15	B11	C22	-62.1(5)
C30	C24	C25	C26	-174.3(5)	B18	B15	B11	C23	-95.7(5)
C30	C24	C22	C23	-15.1(7)	B18	B15	B11	B16	36.9(4)
C30	C24	C22	B9	-155.4(5)	B18	B15	B11	B12	-0.3(6)
C30	C24	C22	B10	-82.3(6)	B18	B14	B10	C22	60.1(6)
C30	C24	C22	B12	124.4(5)	B18	B14	B10	C23	94.8(5)
C30	C24	C22	B11	52.7(6)	B18	B14	B10	B9	-1.4(6)
C19	N3	C20	O3	7.3(8)	B18	B14	B10	B13	-38.5(4)
C19	N3	C20	C21	-174.2(5)	B18	B14	B13	B9	100.2(5)
C23	C22	B9	B10	-38.8(4)	B18	B14	B13	B10	137.7(5)
C23	C22	B9	B13	0.3(5)	B18	B14	B13	B17	37.0(4)
C23	C22	B9	B12	102.0(4)	B18	B13	B17	B9	138.2(5)
C23	C22	B9	B17	63.7(5)	B18	B13	B17	B16	37.3(4)
C23	C22	B10	B9	135.6(4)	B18	B13	B17	B12	100.1(5)
C23	C22	B10	B14	35.1(4)	B18	B16	B12	C22	-61.4(6)
C23	C22	B10	B13	97.2(4)	B18	B16	B12	B9	0.2(6)
C23	C22	B12	B9	-100.2(4)	B18	B16	B12	B17	36.8(5)
C23	C22	B12	B16	-0.2(6)	B18	B16	B12	B11	-100.8(5)
C23	C22	B12	B17	-63.2(5)	B18	B16	B17	B9	-101.3(5)
C23	C22	B12	B11	39.4(4)	B18	B16	B17	B13	-38.0(4)
C23	C22	B11	B15	-33.7(4)	B18	B16	B17	B12	-139.1(5)
C23	C22	B11	B16	-96.5(5)	B18	B16	B11	C22	62.8(6)
C23	C22	B11	B12	-135.9(5)	B18	B16	B11	C23	2.6(6)
C23	B15	B14	B18	-133.0(4)	B18	B16	B11	B15	-37.6(4)
C23	B15	B14	B10	-31.1(4)	B18	B16	B11	B12	101.3(5)
C23	B15	B14	B13	-94.8(5)	B10	C22	C23	B15	-108.9(5)

Table S7 Torsion Angles for cu_20231657_0m_4_sq.

A	B	C	D	Angle/°	A	B	C	D	Angle/°
C23	B15	B18	B14	40.0(4)	B10	C22	C23	B14	-38.8(4)
C23	B15	B18	B13	3.2(6)	B10	C22	C23	B11	-146.4(5)
C23	B15	B18	B16	-97.9(5)	B10	C22	B9	B13	39.1(4)
C23	B15	B18	B17	-59.4(5)	B10	C22	B9	B12	140.8(4)
C23	B15	B16	B18	98.1(5)	B10	C22	B9	B17	102.5(5)
C23	B15	B16	B12	-3.9(6)	B10	C22	B12	B9	-37.0(4)
C23	B15	B16	B17	60.0(6)	B10	C22	B12	B16	63.0(6)
C23	B15	B16	B11	-40.3(4)	B10	C22	B12	B17	0.1(6)
C23	B15	B11	C22	33.6(4)	B10	C22	B12	B11	102.6(5)
C23	B15	B11	B16	132.6(5)	B10	C22	B11	C23	30.6(4)
C23	B15	B11	B12	95.4(5)	B10	C22	B11	B15	-3.1(6)
C23	B14	B18	B15	-39.7(4)	B10	C22	B11	B16	-66.0(5)
C23	B14	B18	B13	98.3(4)	B10	C22	B11	B12	-105.3(5)
C23	B14	B18	B16	-2.0(6)	B10	C23	B15	B14	35.5(4)
C23	B14	B18	B17	61.9(6)	B10	C23	B15	B18	-5.1(6)
C23	B14	B10	C22	-34.6(4)	B10	C23	B15	B16	-66.4(6)
C23	B14	B10	B9	-96.2(4)	B10	C23	B15	B11	-107.4(5)
C23	B14	B10	B13	-133.3(5)	B10	C23	B14	B15	-144.4(5)
C23	B14	B13	B9	1.9(5)	B10	C23	B14	B18	-103.6(5)
C23	B14	B13	B18	-98.3(5)	B10	C23	B14	B13	-40.6(4)
C23	B14	B13	B10	39.4(4)	B10	C23	B11	C22	-33.2(4)
C23	B14	B13	B17	-61.3(5)	B10	C23	B11	B15	108.1(5)
C23	B10	B13	B9	98.1(4)	B10	C23	B11	B16	67.0(6)
C23	B10	B13	B14	-40.1(4)	B10	C23	B11	B12	4.6(6)
C23	B10	B13	B18	-2.2(6)	B10	B9	B13	B14	37.6(4)
C23	B10	B13	B17	61.1(5)	B10	B9	B13	B18	101.2(5)
C39	N5	C40	C41	165.6(5)	B10	B9	B13	B17	138.4(5)
C39	C36	C35	C34	-178.9(5)	B10	B9	B12	C22	35.2(4)
C10	O1	C11	N1	-0.7(7)	B10	B9	B12	B16	-64.5(5)
C10	O1	C11	C12	178.2(5)	B10	B9	B12	B17	-101.8(5)
C10	C6	C7	C8	180.0(5)	B10	B9	B12	B11	-0.9(6)
C28	C27	C32	O4	109.9(5)	B10	B9	B17	B18	0.4(6)
C26	C25	C27	C32	-1.7(8)	B10	B9	B17	B13	-37.5(4)
C26	C25	C27	C28	177.2(5)	B10	B9	B17	B16	63.3(6)
C40	N5	C39	C36	-167.2(5)	B10	B9	B17	B12	100.4(5)
C5	C4	C3	C1	-2.9(8)	B10	B14	B18	B15	-100.0(5)
C5	C4	C3	C9	179.4(5)	B10	B14	B18	B13	38.1(4)
C5	C4	C6	C7	-179.8(5)	B10	B14	B18	B16	-62.2(6)

Table S7 Torsion Angles for cu_20231657_0m_4_sq.

A	B	C	D	Angle/°	A	B	C	D	Angle/°
C5	C4	C6	C10	1.0(7)	B10	B14	B18	B17	1.7(6)
C16	O2	C15	N1	-5.7(7)	B10	B14	B13	B9	-37.4(4)
C16	O2	C15	C14	175.5(5)	B10	B14	B13	B18	-137.7(5)
C41	N6	C42	O6	-2.6(10)	B10	B14	B13	B17	-100.6(5)
C41	N6	C42	C43	178.1(6)	B10	B13	B17	B9	37.2(4)
C42	N6	C41	C40	-77.1(7)	B10	B13	B17	B18	-101.0(5)
B3	C2	B4	C1	-139.4(4)	B10	B13	B17	B16	-63.7(5)
B3	C2	B4	B5	-101.2(4)	B10	B13	B17	B12	-0.9(6)
B3	C2	B4	B7	-39.7(4)	B6	B5	B7	B3	100.0(5)
B3	C2	B1	C1	104.2(4)	B6	B5	B7	B4	138.4(5)
B3	C2	B1	B01Q	66.3(5)	B6	B5	B7	B8	37.8(4)
B3	C2	B1	B01R	3.1(6)	B6	B5	B01Q	C1	-136.1(4)
B3	C2	B1	B2	-37.0(4)	B6	B5	B01Q	B1	-100.4(5)
B3	C2	B2	B1	141.9(4)	B6	B5	B01Q	B01R	-37.6(4)
B3	C2	B2	B01R	102.3(5)	B6	B7	B8	B3	137.4(4)
B3	C2	B2	B8	39.3(4)	B6	B7	B8	B01R	37.1(4)
B3	B4	B5	C1	-98.0(4)	B6	B7	B8	B2	100.2(5)
B3	B4	B5	B7	38.4(4)	B6	B01Q	B01R	B1	-139.6(4)
B3	B4	B5	B01Q	-63.8(5)	B6	B01Q	B01R	B8	-37.4(4)
B3	B4	B5	B6	0.7(5)	B6	B01Q	B01R	B2	-100.3(5)
B3	B4	B7	B5	-136.4(4)	B6	B01R	B8	B3	-99.8(5)
B3	B4	B7	B8	-36.6(4)	B6	B01R	B8	B7	-36.9(4)
B3	B4	B7	B6	-99.7(5)	B6	B01R	B8	B2	-137.0(5)
B3	B7	B8	B01R	-100.2(5)	B6	B01R	B2	C2	-60.9(5)
B3	B7	B8	B2	-37.1(4)	B6	B01R	B2	B3	0.7(6)
B3	B7	B8	B6	-137.4(4)	B6	B01R	B2	B1	-101.3(5)
B3	B7	B6	B5	-99.8(4)	B6	B01R	B2	B8	38.1(4)
B3	B7	B6	B01Q	-63.2(5)	B6	B8	B2	C2	61.0(5)
B3	B7	B6	B01R	0.5(6)	B6	B8	B2	B3	99.8(5)
B3	B7	B6	B8	37.5(4)	B6	B8	B2	B1	-2.4(6)
B3	B8	B2	C2	-38.8(4)	B6	B8	B2	B01R	-38.4(4)
B3	B8	B2	B1	-102.2(5)	B13	B9	B10	C22	-136.1(4)
B3	B8	B2	B01R	-138.2(5)	B13	B9	B10	C23	-98.4(4)
B3	B8	B6	B5	0.6(6)	B13	B9	B10	B14	-36.9(4)
B3	B8	B6	B7	-37.6(4)	B13	B9	B12	C22	99.0(5)
B3	B8	B6	B01Q	63.4(5)	B13	B9	B12	B16	-0.7(6)
B3	B8	B6	B01R	101.0(5)	B13	B9	B12	B17	-38.0(4)
B4	C1	C3	C4	95.0(6)	B13	B9	B12	B11	62.9(6)

Table S7 Torsion Angles for cu_20231657_0m_4_sq.

A	B	C	D	Angle/°	A	B	C	D	Angle/°
B4	C1	C3	C9	-87.1(6)	B13	B9	B17	B18	37.9(5)
B4	C1	C2	B3	38.9(4)	B13	B9	B17	B16	100.8(5)
B4	C1	C2	B1	144.8(4)	B13	B9	B17	B12	137.9(5)
B4	C1	C2	B2	107.9(5)	B13	B14	B18	B15	-138.0(5)
B4	C1	B1	C2	-32.7(4)	B13	B14	B18	B16	-100.3(5)
B4	C1	B1	B01Q	102.9(4)	B13	B14	B18	B17	-36.4(4)
B4	C1	B1	B01R	63.4(5)	B13	B14	B10	C22	98.6(5)
B4	C1	B1	B2	0.3(5)	B13	B14	B10	C23	133.3(5)
B4	C1	B5	B7	-38.8(4)	B13	B14	B10	B9	37.0(4)
B4	C1	B5	B01Q	-141.6(4)	B13	B18	B16	B15	-100.0(5)
B4	C1	B5	B6	-102.5(5)	B13	B18	B16	B12	0.3(6)
B4	C1	B01Q	B1	-104.8(4)	B13	B18	B16	B17	37.3(4)
B4	C1	B01Q	B5	35.9(4)	B13	B18	B16	B11	-62.6(6)
B4	C1	B01Q	B01R	-66.4(5)	B13	B18	B17	B9	-37.5(4)
B4	C1	B01Q	B6	-2.9(6)	B13	B18	B17	B16	-137.9(5)
B4	C2	B3	B7	40.5(4)	B13	B18	B17	B12	-101.8(5)
B4	C2	B3	B8	104.1(5)	B16	B15	B14	C23	95.8(5)
B4	C2	B3	B2	143.6(4)	B16	B15	B14	B18	-37.2(4)
B4	C2	B1	C1	34.5(4)	B16	B15	B14	B10	64.7(6)
B4	C2	B1	B01Q	-3.3(6)	B16	B15	B14	B13	1.0(6)
B4	C2	B1	B01R	-66.5(5)	B16	B15	B18	B14	137.9(5)
B4	C2	B1	B2	-106.6(5)	B16	B15	B18	B13	101.1(5)
B4	C2	B2	B3	-35.6(4)	B16	B15	B18	B17	38.5(4)
B4	C2	B2	B1	106.3(5)	B16	B15	B11	C22	-99.0(5)
B4	C2	B2	B01R	66.7(5)	B16	B15	B11	C23	-132.6(5)
B4	C2	B2	B8	3.7(6)	B16	B15	B11	B12	-37.1(4)
B4	B3	B7	B5	38.7(4)	B16	B18	B13	B9	-0.7(6)
B4	B3	B7	B8	138.6(4)	B16	B18	B13	B14	100.8(5)
B4	B3	B7	B6	100.8(4)	B16	B18	B13	B10	62.7(6)
B4	B3	B8	B7	-37.8(4)	B16	B18	B13	B17	-37.9(4)
B4	B3	B8	B01R	63.3(5)	B16	B18	B17	B9	100.4(5)
B4	B3	B8	B2	100.5(5)	B16	B18	B17	B13	137.9(5)
B4	B3	B8	B6	-0.1(6)	B16	B18	B17	B12	36.2(4)
B4	B3	B2	C2	32.4(4)	B16	B12	B17	B9	-138.1(5)
B4	B3	B2	B1	-2.3(6)	B16	B12	B17	B18	-36.2(4)
B4	B3	B2	B01R	-65.0(5)	B16	B12	B17	B13	-100.5(5)
B4	B3	B2	B8	-102.7(5)	B16	B12	B11	C22	135.4(5)
B4	B5	B7	B3	-38.4(4)	B16	B12	B11	C23	97.9(5)

Table S7 Torsion Angles for cu_20231657_0m_4_sq.

A	B	C	D	Angle/°	A	B	C	D	Angle/°
B4	B5	B7	B8	-100.7(4)	B16	B12	B11	B15	37.0(4)
B4	B5	B7	B6	-138.4(5)	B12	C22	C23	B15	-2.4(6)
B4	B5	B01Q	C1	-34.4(4)	B12	C22	C23	B14	67.7(5)
B4	B5	B01Q	B1	1.3(6)	B12	C22	C23	B10	106.5(5)
B4	B5	B01Q	B01R	64.1(5)	B12	C22	C23	B11	-40.0(4)
B4	B5	B01Q	B6	101.7(5)	B12	C22	B9	B10	-140.8(4)
B4	B5	B6	B7	37.5(4)	B12	C22	B9	B13	-101.7(5)
B4	B5	B6	B01Q	-101.2(4)	B12	C22	B9	B17	-38.3(4)
B4	B5	B6	B01R	-64.0(5)	B12	C22	B10	C23	-97.8(4)
B4	B5	B6	B8	-0.8(6)	B12	C22	B10	B9	37.8(4)
B4	B7	B8	B3	36.6(4)	B12	C22	B10	B14	-62.7(5)
B4	B7	B8	B01R	-63.6(5)	B12	C22	B10	B13	-0.6(6)
B4	B7	B8	B2	-0.5(6)	B12	C22	B11	C23	135.9(5)
B4	B7	B8	B6	-100.7(4)	B12	C22	B11	B15	102.1(5)
B4	B7	B6	B5	-37.2(4)	B12	C22	B11	B16	39.3(4)
B4	B7	B6	B01Q	-0.5(6)	B12	B9	B10	C22	-34.3(4)
B4	B7	B6	B01R	63.1(6)	B12	B9	B10	C23	3.4(5)
B4	B7	B6	B8	100.2(5)	B12	B9	B10	B14	64.9(5)
B1	C1	C3	C4	-47.8(7)	B12	B9	B10	B13	101.8(5)
B1	C1	C3	C9	130.1(5)	B12	B9	B13	B14	-62.7(6)
B1	C1	C2	B3	-105.9(4)	B12	B9	B13	B18	0.9(6)
B1	C1	C2	B4	-144.8(4)	B12	B9	B13	B10	-100.3(5)
B1	C1	C2	B2	-36.9(4)	B12	B9	B13	B17	38.1(4)
B1	C1	B4	C2	33.4(4)	B12	B9	B17	B18	-100.1(5)
B1	C1	B4	B3	-1.7(5)	B12	B9	B17	B13	-137.9(5)
B1	C1	B4	B5	-102.1(4)	B12	B9	B17	B16	-37.2(4)
B1	C1	B4	B7	-63.9(5)	B12	B16	B17	B9	37.8(4)
B1	C1	B5	B4	105.3(4)	B12	B16	B17	B18	139.1(5)
B1	C1	B5	B7	66.5(5)	B12	B16	B17	B13	101.2(5)
B1	C1	B5	B01Q	-36.3(4)	B12	B16	B11	C22	-38.5(4)
B1	C1	B5	B6	2.8(5)	B12	B16	B11	C23	-98.7(5)
B1	C1	B01Q	B5	140.7(4)	B12	B16	B11	B15	-138.9(5)
B1	C1	B01Q	B01R	38.3(4)	B17	B9	B10	C22	-98.6(5)
B1	C1	B01Q	B6	101.9(5)	B17	B9	B10	C23	-60.9(5)
B1	C2	B3	B4	-107.0(4)	B17	B9	B10	B14	0.6(6)
B1	C2	B3	B7	-66.5(5)	B17	B9	B10	B13	37.5(4)
B1	C2	B3	B8	-2.9(6)	B17	B9	B13	B14	-100.8(5)
B1	C2	B3	B2	36.6(4)	B17	B9	B13	B18	-37.2(4)

Table S7 Torsion Angles for cu_20231657_0m_4_sq.

A	B	C	D	Angle/°	A	B	C	D	Angle/°
B1	C2	B4	C1	-34.1(4)	B17	B9	B13	B10	-138.4(5)
B1	C2	B4	B3	105.3(5)	B17	B9	B12	C22	137.0(4)
B1	C2	B4	B5	4.1(6)	B17	B9	B12	B16	37.3(4)
B1	C2	B4	B7	65.6(5)	B17	B9	B12	B11	100.9(5)
B1	C2	B2	B3	-141.9(4)	B17	B18	B13	B9	37.1(4)
B1	C2	B2	B01R	-39.7(4)	B17	B18	B13	B14	138.6(5)
B1	C2	B2	B8	-102.6(5)	B17	B18	B13	B10	100.6(5)
B1	B01Q	B01R	B8	102.2(5)	B17	B18	B16	B15	-137.2(4)
B1	B01Q	B01R	B2	39.4(4)	B17	B18	B16	B12	-37.0(4)
B1	B01Q	B01R	B6	139.6(4)	B17	B18	B16	B11	-99.8(5)
B1	B01Q	B6	B5	102.6(4)	B17	B16	B12	C22	-98.2(5)
B1	B01Q	B6	B7	65.6(6)	B17	B16	B12	B9	-36.5(4)
B1	B01Q	B6	B01R	-35.8(4)	B17	B16	B12	B11	-137.6(5)
B1	B01Q	B6	B8	1.7(6)	B17	B16	B11	C22	-0.6(6)
B1	B01R	B8	B3	0.5(6)	B17	B16	B11	C23	-60.7(5)
B1	B01R	B8	B7	63.4(6)	B17	B16	B11	B15	-100.9(5)
B1	B01R	B8	B2	-36.7(4)	B17	B16	B11	B12	38.0(4)
B1	B01R	B8	B6	100.3(5)	B17	B12	B11	C22	97.4(5)
B1	B01R	B2	C2	40.4(4)	B17	B12	B11	C23	59.8(5)
B1	B01R	B2	B3	102.0(5)	B17	B12	B11	B15	-1.1(6)
B1	B01R	B2	B8	139.4(5)	B17	B12	B11	B16	-38.1(4)
B1	B01R	B6	B5	-0.9(6)	B11	C22	C23	B15	37.6(5)
B1	B01R	B6	B7	-64.5(5)	B11	C22	C23	B14	107.7(5)
B1	B01R	B6	B01Q	36.0(4)	B11	C22	C23	B10	146.4(5)
B1	B01R	B6	B8	-101.8(5)	B11	C22	B9	B10	-102.8(5)
B9	C22	C23	B15	-69.1(5)	B11	C22	B9	B13	-63.7(5)
B9	C22	C23	B14	1.0(5)	B11	C22	B9	B12	38.0(4)
B9	C22	C23	B10	39.8(4)	B11	C22	B9	B17	-0.3(6)
B9	C22	C23	B11	-106.6(4)	B11	C22	B10	C23	-30.9(4)
B9	C22	B10	C23	-135.6(4)	B11	C22	B10	B9	104.6(5)
B9	C22	B10	B14	-100.5(5)	B11	C22	B10	B14	4.2(6)
B9	C22	B10	B13	-38.4(4)	B11	C22	B10	B13	66.2(5)
B9	C22	B12	B16	99.9(5)	B11	C22	B12	B9	-139.6(5)
B9	C22	B12	B17	37.0(4)	B11	C22	B12	B16	-39.6(4)
B9	C22	B12	B11	139.6(5)	B11	C22	B12	B17	-102.5(5)
B9	C22	B11	C23	97.1(5)	B11	C23	B15	B14	142.9(5)
B9	C22	B11	B15	63.4(5)	B11	C23	B15	B18	102.3(5)
B9	C22	B11	B16	0.5(6)	B11	C23	B15	B16	40.9(4)

Table S7 Torsion Angles for cu_20231657_0m_4_sq.

A	B	C	D	Angle/°	A	B	C	D	Angle/°
B9	C22	B11	B12	-38.8(4)	B11	C23	B14	B15	-36.8(4)
B9	B10	B13	B14	-138.2(5)	B11	C23	B14	B18	4.0(6)
B9	B10	B13	B18	-100.3(5)	B11	C23	B14	B10	107.6(5)
B9	B10	B13	B17	-37.0(4)	B11	C23	B14	B13	67.1(5)
B9	B13	B17	B18	-138.2(5)	B11	C23	B10	C22	32.9(4)
B9	B13	B17	B16	-100.9(5)	B11	C23	B10	B9	-5.2(6)
B9	B13	B17	B12	-38.1(4)	B11	C23	B10	B14	-107.2(5)
B9	B12	B17	B18	101.9(5)	B11	C23	B10	B13	-66.9(5)
B9	B12	B17	B13	37.7(4)	B11	B15	B14	C23	32.7(4)
B9	B12	B17	B16	138.1(5)	B11	B15	B14	B18	-100.3(5)
B9	B12	B11	C22	35.6(4)	B11	B15	B14	B10	1.6(6)
B9	B12	B11	C23	-2.0(6)	B11	B15	B14	B13	-62.1(6)
B9	B12	B11	B15	-62.8(5)	B11	B15	B18	B14	100.8(5)
B9	B12	B11	B16	-99.8(5)	B11	B15	B18	B13	64.0(6)
B5	C1	C3	C4	167.8(5)	B11	B15	B18	B16	-37.0(4)
B5	C1	C3	C9	-14.4(7)	B11	B15	B18	B17	1.5(6)
B5	C1	C2	B3	-1.5(5)	B11	B15	B16	B18	138.4(5)
B5	C1	C2	B4	-40.4(4)	B11	B15	B16	B12	36.4(4)
B5	C1	C2	B1	104.5(4)	B11	B15	B16	B17	100.2(5)
B5	C1	C2	B2	67.5(5)	B11	B16	B12	C22	39.4(4)
B5	C1	B4	C2	135.4(4)	B11	B16	B12	B9	101.1(5)
B5	C1	B4	B3	100.4(4)	B11	B16	B12	B17	137.6(5)
B5	C1	B4	B7	38.1(4)	B11	B16	B17	B9	0.4(6)
B5	C1	B1	C2	-99.5(4)	B11	B16	B17	B18	101.7(5)
B5	C1	B1	B01Q	36.2(4)	B11	B16	B17	B13	63.8(6)
B5	C1	B1	B01R	-3.3(5)	B11	B16	B17	B12	-37.4(4)
B5	C1	B1	B2	-66.4(5)	B11	B12	B17	B9	-100.0(5)
B5	C1	B01Q	B1	-140.7(4)	B11	B12	B17	B18	2.0(6)
B5	C1	B01Q	B01R	-102.4(4)	B11	B12	B17	B13	-62.3(5)
B5	C1	B01Q	B6	-38.8(4)	B11	B12	B17	B16	38.2(4)

Table S8 Hydrogen Atom Coordinates (Å×10^4) and Isotropic Displacement Parameters (Å^2×10^3) for cu_20231657_0m_4_sq.

Atom	*x*	*y*	*z*	U(eq)
H2A	11604.13	4481.59	8628.09	51
H2B	10381.69	4152.32	8840.54	51

Table S8 Hydrogen Atom Coordinates (Å×10^4) and Isotropic Displacement Parameters (Å^2×10^3) for cu_20231657_0m_4_sq.

Atom	*x*	*y*	*z*	U(eq)
H5D	6706.91	4998.44	8344.48	57
H5E	5359.18	5331.39	8172.85	57
H18A	13814.42	4439.97	9209.08	55
H18B	12614.96	4051.44	9432.6	55
H13	13048.94	4127.87	7657.34	56
H12	11260.84	3929.01	6994.25	55
H34	6118.37	4110.71	6817.26	62
H9	3657.93	2927.46	5394.91	53
H38A	2598.26	5813.78	6885.79	80
H38B	2353.28	6076.46	7354.92	80
H38C	1458.57	5595.59	7294.17	80
H17A	12654.29	3608.31	8731.58	57
H17B	13868.51	3970.54	8469.59	57
H7	3618.97	3449.6	6659.54	64
H32A	1698.95	4930.54	6204.6	63
H32B	464.31	4625.31	6533.07	63
H2	8792.27	2021.92	5827.66	53
H35	8016.61	4460.91	7384.37	62
H30	-1488.27	3770.18	4891.96	61
H8	2159.18	3357.14	5932.07	66
H19A	12088.38	4743.87	9800.03	60
H19B	11229.68	4933.49	9328.04	60
H23	-244.52	3471.52	4409.56	58
H39A	8822.48	5135.09	7803.26	63
H39B	7701.52	5573.62	7658.87	63
H10A	5762.96	3294.81	7212.06	64
H10B	7141.34	2876.57	7163.93	64
H28	-1580.67	4130.41	6215.75	65
H26A	3245.26	4919.16	5643.83	80
H26B	3450.16	4783.1	5118.68	80
H26C	4466.13	4495.96	5512.55	80
H40A	8674.51	5493.68	8670.13	69
H40B	7620.4	5883.76	8396.56	69
H5A	8525.99	2305.03	6467.01	85
H5B	9269.49	2776.94	6634.94	85
H5C	9622.68	2618.26	6122.24	85
H29	-2942.91	3749.94	5593.75	65

Table S8 Hydrogen Atom Coordinates (Å×10^4) and Isotropic Displacement Parameters (Å^2×10^3) for cu_20231657_0m_4_sq.

Atom	*x*	*y*	*z*	U(eq)
H21A	7255.99	3848.72	10261.68	91
H21B	6724.45	4031.48	9761.49	91
H21C	6384.63	4331.93	10201.33	91
H16A	7555.94	2994.01	8865.4	89
H16B	7780.81	2842.37	8345.47	89
H16C	6637.92	3282.16	8458.93	89
H41A	5880.62	5373.45	9086.66	71
H41B	6747.55	5853.84	9174.9	71
H3A	8377.58	1340.82	5155.15	52
H4	5051.2	1784.69	5528.04	53
H1	10059.31	2849.1	5417.56	55
H9A	5188.97	4253.04	4779.78	58
H5	3873.65	2474.78	4855.45	52
H7A	5261.86	1617.16	4525.56	55
H01Q	6893.93	3117.35	4809.76	52
H01R	10317.09	2673.28	4442.63	58
H15	-275.93	3716.4	3464.71	64
H8A	9297.71	1743.17	4270.44	61
H14	2650.47	3107.44	3841.06	59
H2C	11453.34	1985.09	5117.05	61
H18	3783.7	3838.6	3229.82	63
H10	3425.34	3370.95	4799.97	59
H6	6453.57	2445.82	4085.39	62
H13A	6093.31	3609.51	4068.87	60
H16	1470.83	4609.83	3439.72	68
H12A	2265.12	4876.64	4397.74	60
H17	5412.63	4537.93	3827.01	63
H11	-1157.89	4363.58	4181.74	63
H43A	1902.28	6715.72	8663.62	144
H43B	1250.21	6213.02	8618.26	144
H43C	2184.87	6456.18	8194.06	144
H3	8550(60)	4460(20)	9456(19)	70(20)
H6A	3410(70)	5673(17)	8720(20)	67(19)

Table S9 Solvent masks information for cu_20231657_0m_4_sq.

Number	X	Y	Z	Volume	Electron count	Content
1	0.500	0.500	0.000	29	1	
2	0.710	0.790	0.227	252	42	
3	0.810	0.290	0.273	251	42	
4	0.000	0.000	0.500	29	1	
5	0.222	0.710	0.727	253	42	
6	0.313	0.210	0.773	252	42	
